# Ligand Profiling as a Diagnostic Tool to Differentiate Patient-Derived α-Synuclein Polymorphs

**DOI:** 10.1021/acschemneuro.4c00178

**Published:** 2024-05-01

**Authors:** Timothy S. Chisholm, Ronald Melki, Christopher A. Hunter

**Affiliations:** † Yusuf Hamied Department of Chemistry, University of Cambridge, Lensfield Road, Cambridge CB2 1EW, U.K.; ‡ Institut François Jacob (MIRCen), CEA, CNRS, University Paris-Saclay, 18 Route du Panorama, 92260 Fontenay-aux-Roses, France

**Keywords:** α-synuclein, ligand binding site, fluorescence binding assay, polymorphs, protein misfolding cyclic amplification, Parkinson’s disease

## Abstract

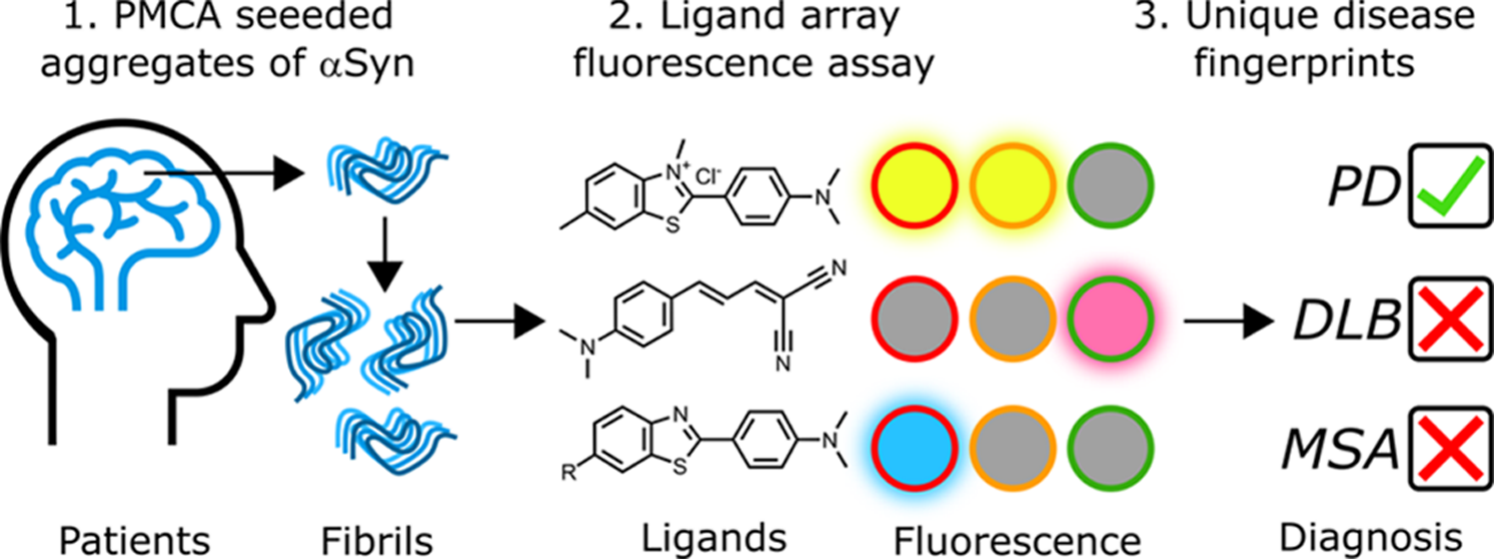

Amyloid fibrils are characteristic of many neurodegenerative diseases, including Alzheimer’s and Parkinson’s diseases. While different diseases may have fibrils formed of the same protein, the supramolecular morphology of these fibrils is disease-specific. Here, a method is reported to distinguish eight morphologically distinct amyloid fibrils based on differences in ligand binding properties. Eight fibrillar polymorphs of α-synuclein (αSyn) were investigated: five generated de novo using recombinant αSyn and three generated using protein misfolding cyclic amplification (PMCA) of recombinant αSyn seeded with brain homogenates from deceased patients diagnosed with Parkinson’s disease (PD), multiple system atrophy (MSA), and dementia with Lewy bodies (DLB). Fluorescence binding assays were carried out for each fibril using a toolkit of six different ligands. The fibril samples were separated into five categories based on a binary classification of whether they bound specific ligands or not. Quantitative binding measurements then allowed every fibrillar polymorph to be uniquely identified, and the PMCA fibrils derived from PD, MSA, and DLB patients could be unambiguously distinguished. This approach constitutes a novel and operationally simple method to differentiate amyloid fibril morphologies and to identify disease states using PMCA fibrils obtained by seeding with patient samples.

## Introduction

Parkinson’s disease (PD) is the most common neurodegenerative movement disorder and the most common synucleinopathy.^1−4^ Synucleinopathies are a diverse class of neurodegenerative diseases that also include multiple system atrophy (MSA) and dementia with Lewy bodies (DLB).^5−8^ These diseases are characterized by the formation of fibrillar aggregates of the protein α-synuclein (αSyn) in the brain. αSyn is a 140-residue intrinsically disordered protein with a native function that has not been fully elucidated.^9−11^ All synucleinopathies are characterized by two types of αSyn-rich insoluble protein deposits in neurons called Lewy bodies and Lewy neurites.^12−16^ What distinguishes MSA from PD and DLB are the oligodendroglia as glial cytoplasmic αSyn-rich inclusions.^15,17^ The precise role of αSyn and these insoluble deposits in the pathology of synucleinopathies is not yet fully understood, although mutations in the *SNCA* gene which encodes αSyn are associated with early onset familial forms of synucleinopathies.^18−21^

There is increasing evidence that morphologically distinct αSyn fibrils are involved in different synucleinopathies.^22−33^ Fibrils with different intrinsic structures and morphologies that have been assembled in vitro also have different chemical and biological properties.^34−36^ Inoculating different recombinant αSyn fibrils into rat substantia nigra produced distinct symptoms and αSyn pathologies.^29^ Recently, cryo-electron microscopy studies have reported structures for αSyn fibrils and protofilaments derived from PD, DLB, and MSA brain homogenates. Yang et al. reported that αSyn fibrils isolated from PD, PD dementia, and DLB patients share a common protofilament fold that is distinct to MSA fibrils.^37,38^

It is also possible to use brain homogenates from patient samples to seed the aggregation of recombinant αSyn using protein misfolding cyclic amplification (PMCA).^30^ Although the PMCA fibrils obtained in this process may not be a precise replica of the seeds, the morphologies of PMCA fibrils have been shown to depend on the nature of the seeds used. The morphology of PMCA fibrils of αSyn seeded with brain homogenates from PD patients was found to be different from PMCA fibrils seeded with brain homogenates from MSA patients.^39^ This result suggests that the PMCA seeding process can be used to convert small amounts of fibril present in patient samples into large amounts of synthetic fibrils and that the structures of the resulting PMCA fibrils can report on disease states.

PMCA fibrils of αSyn seeded from PD, MSA, and DLB brain homogenates all have distinct chemical and biological properties to one another.^30,31,39^ Moreover, PMCA fibrils prepared using brain homogenates from different patients with the same disease are found to exhibit the same characteristic patterns of limited proteolysis and structural features observed by transmission electron microscopy (TEM).^30,31,39^ The fact that the structures and properties of PMCA fibrils are uniquely determined by the nature of the seeds suggests that the pathological origins of brain homogenates used for PMCA seeding could be determined by identifying the morphologies of the resulting fibrils. However, straightforward methods to distinguish different fibril morphologies are currently limited.

**Figure 1:**
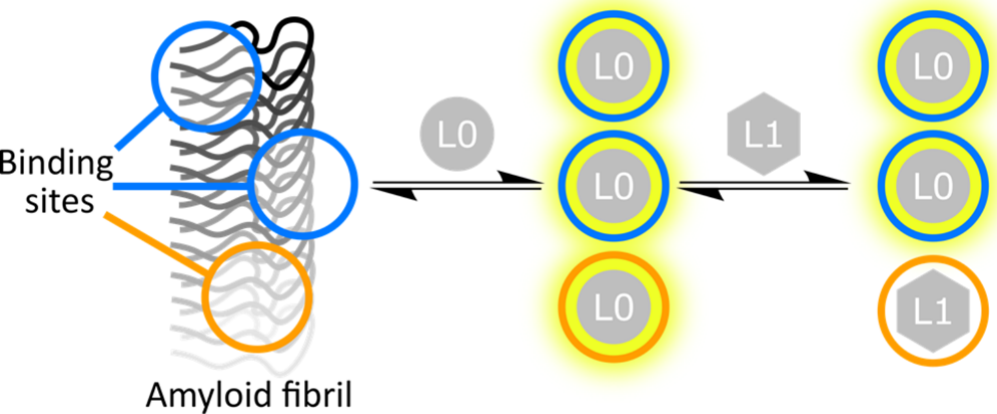
Competitive ligand binding assay performed on an amyloid fibril. The blue and orange circles denote different binding sites on the fibril. A solvatochromic ligand, L0, binds to both the blue and orange sites leading to an increase in fluorescence. Addition of a nonfluorescent second ligand, L1, that only binds to orange sites leads to a partial decrease in fluorescence.

We have previously shown that amyloid fibrils contain a collection of binding sites which are morphology-dependent.^40^ Different ligands and different binding assays report on different ligand binding sites.^41−53^ These binding sites, and the fibril morphology, can therefore be characterized by ligand binding assays. Figure 1 shows a schematic illustration of the experiment for a fibril that has two different types of binding site (blue and orange). Solvatochromic ligand L0 binds to all of the sites with an increase in fluorescence emission intensity in the bound state. A competing nonfluorescent ligand L1 that only binds to orange sites will displace L0 from these sites, leading to a partial decrease of the measured fluorescence. Fluorescence binding assays with various combinations of L0 and L1 can therefore inform on different binding sites present on the fibril.

Using a diverse array of ligands allows a wide range of fibril structural features to be probed by targeting a diverse set of binding sites. Identification of unique ligand profiles can therefore be used to develop binding assays that distinguish different fibril morphologies. Here, the presence of morphology-dependent binding sites was used to distinguish eight different fibril morphologies of αSyn. Five αSyn fibrillar polymorphs were generated de novo.^34−36,39,54,55^ These de novo polymorphs possess different chemical and biological properties that are distinct from the fibrils that we used previously to develop the approach,^40^ and therefore provide an independent test of the scope of the methodology. Three types of αSyn fibrils were derived from the brain homogenates of patients suffering from PD, MSA, and DLB using the PMCA process.^30,31,39^ Although these fibrils are largely composed of recombinant αSyn, the morphologies of the PMCA fibrils have been shown to be determined by the seeding capabilities of the pathogenic αSyn aggregates present in the brain homogenates of the synucleinopathy patients. Six different ligands were used in fluorescence assays to identify differences in binding sites present on these fibrils. The results of these assays were used to construct decision trees that allow all eight fibril morphologies to be uniquely identified using just four ligand binding assays.

## Results and Discussion

### Amyloid Ligands

The six ligands shown in Figure 2 were selected based on structural diversity to allow a wide range of binding sites to be sampled. Thioflavin T (ThT) and AAR are solvatochromic fluorescent ligands, allowing changes in fluorescence intensity to be used to monitor binding.^56−58^ BTA is a nonsolvatochromic fluorescent ligand, so changes in fluorescence anisotropy can be used to monitor binding. OXI, S5H, and thiazine red (ThR) do not fluoresce strongly and were used as competing ligands (L1) in the fluorescence displacement assay shown in Figure 1.^59−61^ S5H was obtained and used as a mixture of the 3,5-isoxazole and 5,3-isoxazole isomers as previously reported.^60^ The characterization of all synthesized/purified ligands is given in Supporting Figures S1−S23, and their spectroscopic properties are given in Supporting Figures S27−S43 and Supporting Tables S1 and S2.

**Figure 2:**
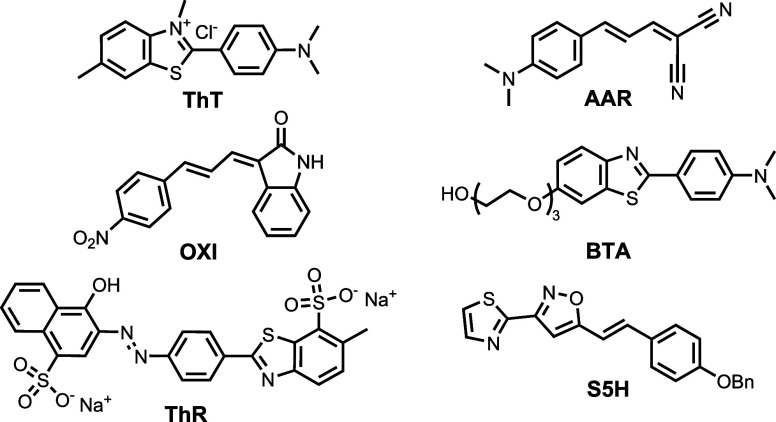
Structures of the ligands used in binding assays.

### Preparation of αSyn Fibrils

Five de novo and three PMCA fibrillar polymorphs were prepared according to previously reported procedures. Recombinant wild-type full-length human αSyn and wild-type human C-terminally truncated (1−110) αSyn were expressed in *Escherichia coli* and purified.^62^ Pure αSyn and αSyn 1−110 were filtered through sterile 0.22 μm filters, and the concentration was determined spectrophotometrically before storage in 50 mM Tris·HCl, pH 7.5, 150 mM KCl at −80 °C until use.

Structurally distinct de novo fibrillar polymorphs were obtained by incubating αSyn at 37 °C with shaking (600 rpm) in the storage buffer or after dialysis against different buffers. The buffer conditions used to prepare the de novo fibril strains are shown in Table 1. Fibrils (F) were aggregated without buffer exchange under neutral conditions at physiological ionic strength. Ribbons (R) were prepared under neutral and low salt conditions in 5 mM Tris·HCl, pH 7.5. Fibrils-65 (f65) were prepared with acidic conditions in 20 mM MES, pH 6.5, and fibrils-91 (f91) were prepared with basic conditions in 20 mM KPO_4_, pH 9.1. Fibrils-110 (f110) were assembled from truncated (1−110) αSyn without buffer exchange. Aggregation was monitored by ThT binding (λ_ex_ = 440 nm, λ_em_ = 480 nm). These fibrils have been extensively characterized in prior work and are known to have different morphologies, providing an excellent test system for establishing the utility of the ligand binding assay to distinguish different fibrils of the same protein.^34−36,39,54,55^

**Table 1:** Conditions Used to Prepare the Five De Novo Fibrillar Polymorphs

de novo polymorph	conditions
fibril (F)	50 mM tris·HCl, 150 mM KCl, pH 7.5, 37 °C
ribbon (R)	5 mM tris·HCl, pH 7.5, 37 °C
fibril-65 (f65)	20 mM MES, pH 6.5, 37 °C
fibril-91 (f91)	20 mM KPO4, pH 9.1, 37 °C
fibril-110 (f110)	50 mM Tris·HCl, 150 mM KCl, pH 7.5, 37 °C (using truncated (1−110) αSyn)

The PMCA αSyn fibrillar polymorphs were obtained by performing PMCA on brain homogenates from PD, DLB, and MSA patients. Briefly, brain homogenates were diluted (2 vol %) into fresh recombinant αSyn (100 μM) and submitted to 15 s of sonication followed by a 5 min pause sequences at 30 °C. A 5 μL aliquot was withdrawn every hour and added to ThT to monitor aggregation. After 430 min, the reaction product was diluted (2 vol %) into fresh αSyn (100 μM). Two additional 200 min amplification cycles were performed, followed by a final dilution (5 vol %) into fresh αSyn (100 μM) and overnight incubation at 30 °C to allow the fibrils to elongate.

Circular dichroism showed all eight fibril samples had a minimum at 220 nm, characteristic of β-sheets (Figure S24). Fibrils were characterized by TEM, demonstrating differences in length, curvature, and lateral association (Figure S25). Limited proteolysis patterns were in agreement with previous preparations of these fibrils (Figure S26).

### Binding Assays

Direct binding assays were first performed with ThT and AAR by monitoring fluorescence intensity, and with BTA by monitoring fluorescence anisotropy (Figures S44−S46, Table S5). Competition binding assays were performed with ThT as L0 and S5H, OXI, BTA, or ThR as L1, and with AAR as L0 and ThT as L1 (Figures 1 and S47−S54, Tables S6−S11). The fluorescence intensity of L0 was monitored for all of these assays. Representative titration data are shown in Figure 3.

**Figure 3:**
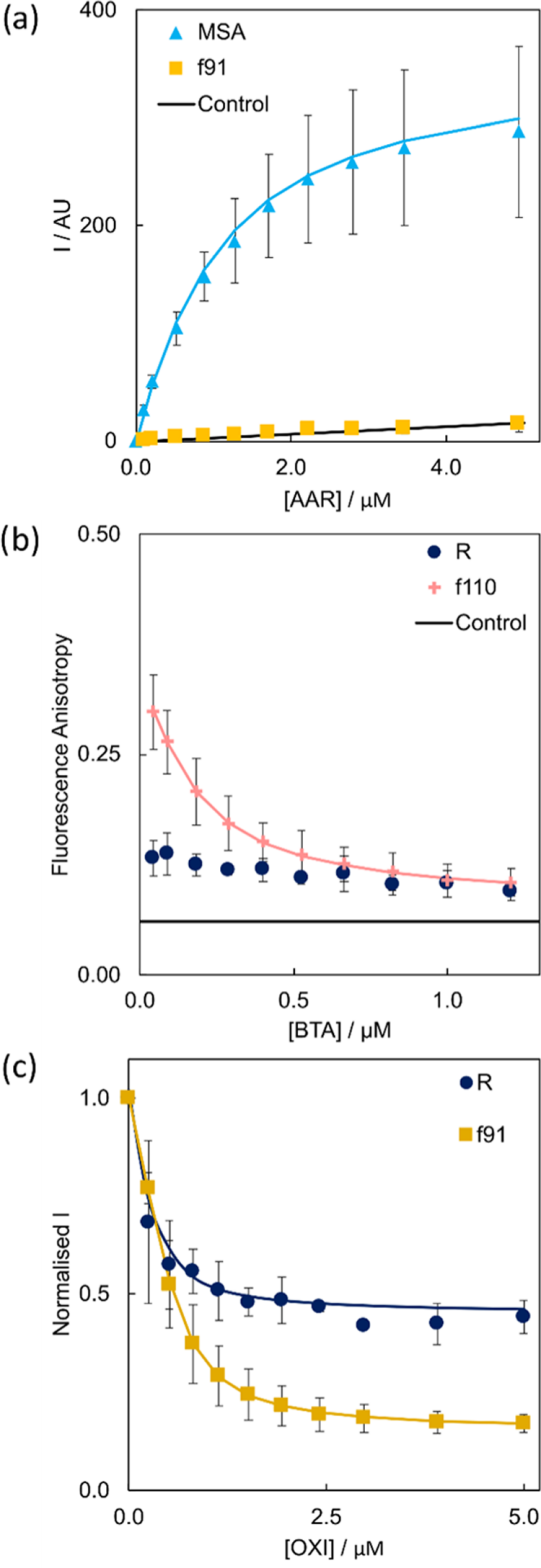
Titration data from (a) a direct fluorescence assay of AAR (λ_ex_ = 522 nm, λ_em_ = 573 nm) with MSA and f91 fibrils, (b) a direct fluorescence anisotropy assay of BTA (λ_ex_ = 360 nm, λ_em_ = 443 nm) with R and f110 fibrils, and (c) a fluorescence competition assay using ThT as L0 (1.0 μM) and OXI as L1 (λ_ex_ = 440 nm, λ_em_ = 483 nm) with F and R fibrils. All assays were performed in aqueous 1 × PBS (pH 7.4, 25 °C) with 500 nM αSyn fibrils. Data points are the average of at least three experimental measurements with 95% confidence intervals shown, and lines are the best fit to a 1:1 binding isotherm.

These titrations demonstrated clear differences in the binding of ligands to the eight fibril morphologies. The direct fluorescence titration of AAR, in Figure 3a, showed that AAR binds to MSA fibrils with an increase in fluorescence intensity. In contrast, no binding of AAR was observed for f91 fibrils. The direct fluorescence anisotropy assay in Figure 3b showed that BTA binds to f110 fibrils with a change in fluorescence anisotropy, but no change was observed for R fibrils.

Fluorescence competition assays showed differences not only in the ability of a ligand to bind to different fibrils but also in the amount of reporting ligand displaced by the competing ligand. The fluorescence competition assay in Figure 3c used ThT as L0 and OXI as L1. OXI displaced ThT from both f91 and R fibrils, but the difference in the final fluorescence intensity measured in these two experiments indicates that OXI accesses a greater proportion of the ThT binding sites on f91 fibrils than on R fibrils.

In addition to the qualitative observations in Figure 3, the titration experiments can be used to derive two quantitative parameters for the ligand–fibril interactions: the dissociation constant (*K*_d_) and the percentage of L0 binding sites accessible to L1 in a competition assay (%BS1). Competition assay data were analyzed in terms of two different binding sites: BS1 is accessible to both L0 and L1, and BS2 is only accessible to L0. In Figure 3c, the fluorescence observed at the end of the titrations is due to ThT bound to BS2, and the change in fluorescence observed on addition of L1 is due to the displacement of ThT from BS1. Thus, the relative concentrations of the two binding sites can be calculated from the fluorescence intensities at the start and end of the titration. In all cases, the titration data were fitted to 1:1 binding isotherms to measure the dissociation constant (*K*_d_) and the limiting value of %BS1 for fully bound L1 (Figures S44−S54, Tables S5−S11).

**Figure 4:**
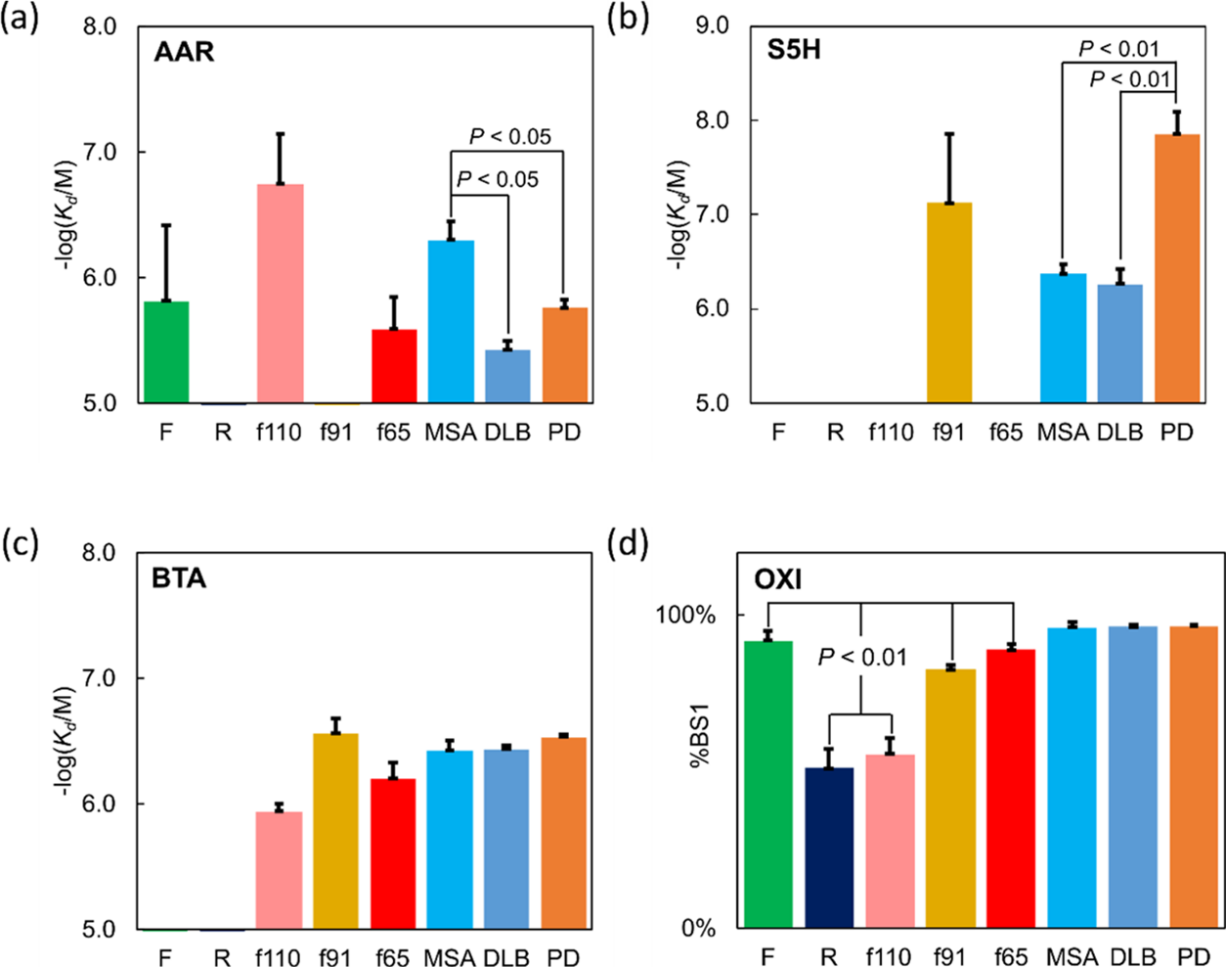
Comparisons of the binding constants for (a) a direct binding assay using AAR (L0), (b) a ThT (L0) competition binding assay with S5H (L1), and (c) a ThT (L0) competition binding assay with BTA (L1). (d) Comparison of the %BS1 values from a ThT (L0) competition binding assay with OXI (L1). The absence of a bar indicates no binding was observed. Data points are the average of at least three experimental measurements with 95% confidence intervals shown, and *P* values were calculated using a two-sided paired *t*-test.

Qualitative observations of the titrations performed, such as those shown in Figure 3, were used to separate the eight fibril morphologies into five distinguishable groups. Quantitative analysis of the titration data was then used to distinguish all eight fibrils based on differences in interactions with five of the ligands shown in Figure 2. The key results that allow the eight fibrils to be distinguished are summarized in Figure 4 and explained in more detail below.

Four different experiments are required. First, direct titration of AAR identified R and f91 which do not bind AAR (Figure 4a). Differences in the *K*_d_ values from this experiment also allowed MSA to be distinguished from DLB and PD fibrils (*P* < 0.05). Second, a competition assay using ThT (L0) and S5H (L1) identified f91 as the only de novo fibril that binds S5H (Figure 4b). Differences in the *K*_d_ values from this experiment also allowed PD to be distinguished from DLB and MSA (*P* < 0.01). Third, a competition assay using ThT (L0) and BTA (L1) identified F and R, as neither fibril shows displacement of ThT (Figure 4c). Finally, a competition assay using ThT (L0) and OXI (L1) identified R and f110, as these two fibrils have significantly lower values of %BS1 than the other morphologies that showed almost complete displacement of L0 (Figure 4d).

### Protocol for Differentiating Fibril Morphologies

**Figure 5:**
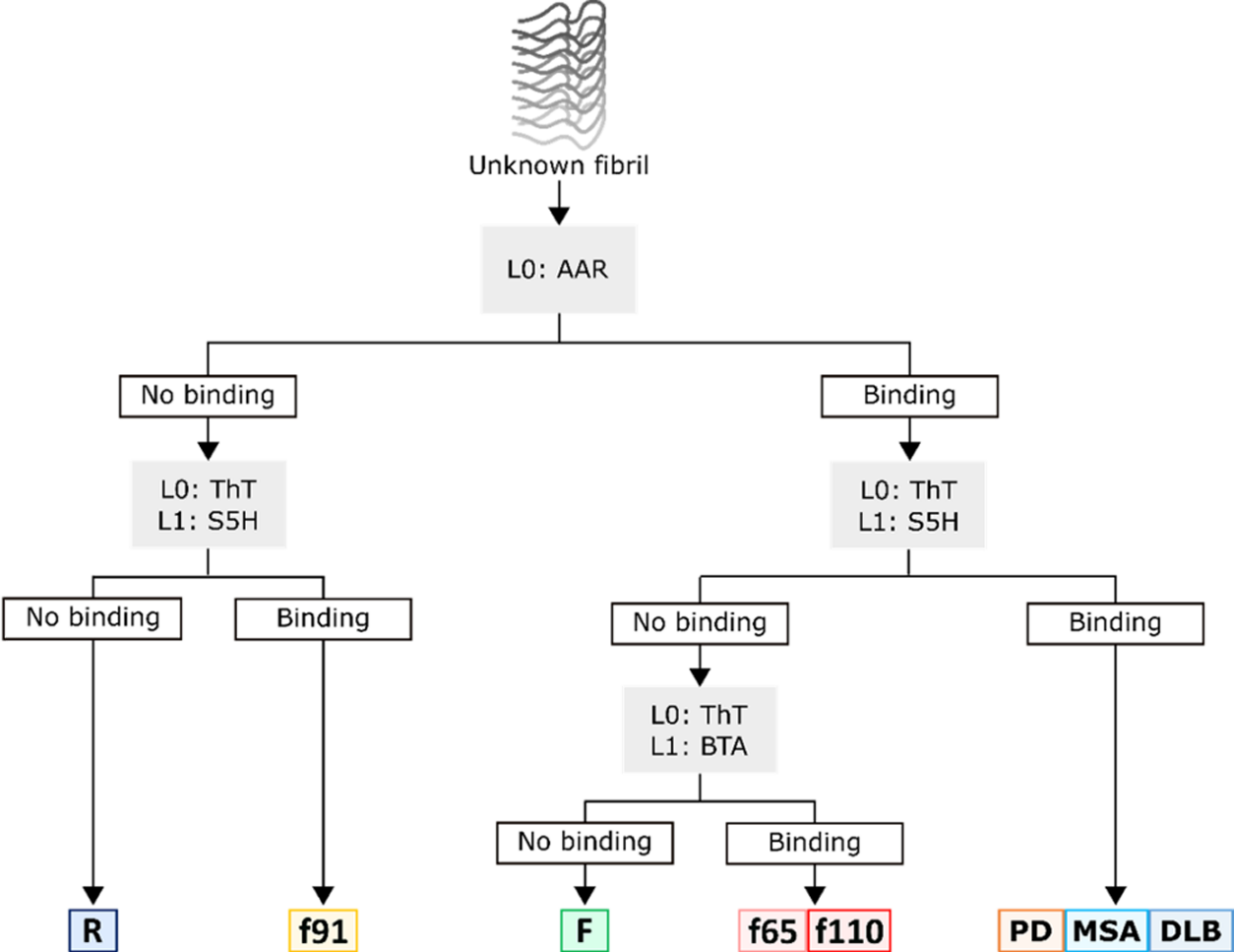
Decision tree for identifying αSyn fibrillar polymorphs based on a binary classification of whether ligand binding is observed or not. The assays used are shown in gray boxes, and L0 and L1 indicate the role of the ligand in direct and competition assays as outlined in Figure 1.

**Figure 6:**
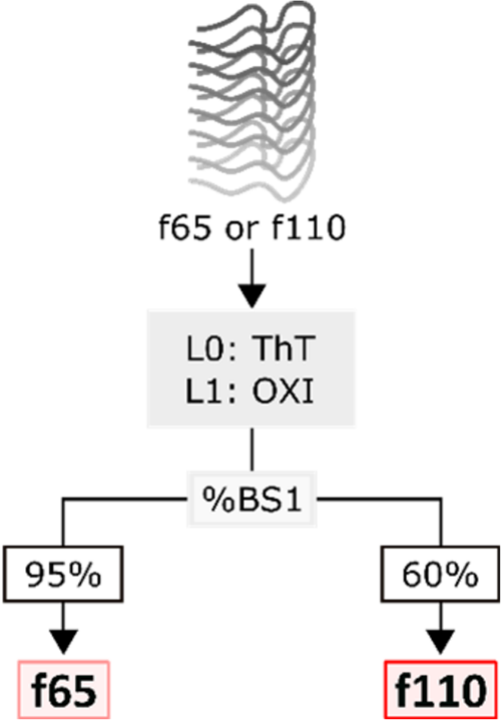
Decision tree for distinguishing f65 and f110 fibrils based on the proportion of ThT binding sites shared by OXI (%BS1). The competition assay used is shown in the gray box, and L0 and L1 indicate the role of the ligand as outlined in Figure 1.

**Figure 7:**
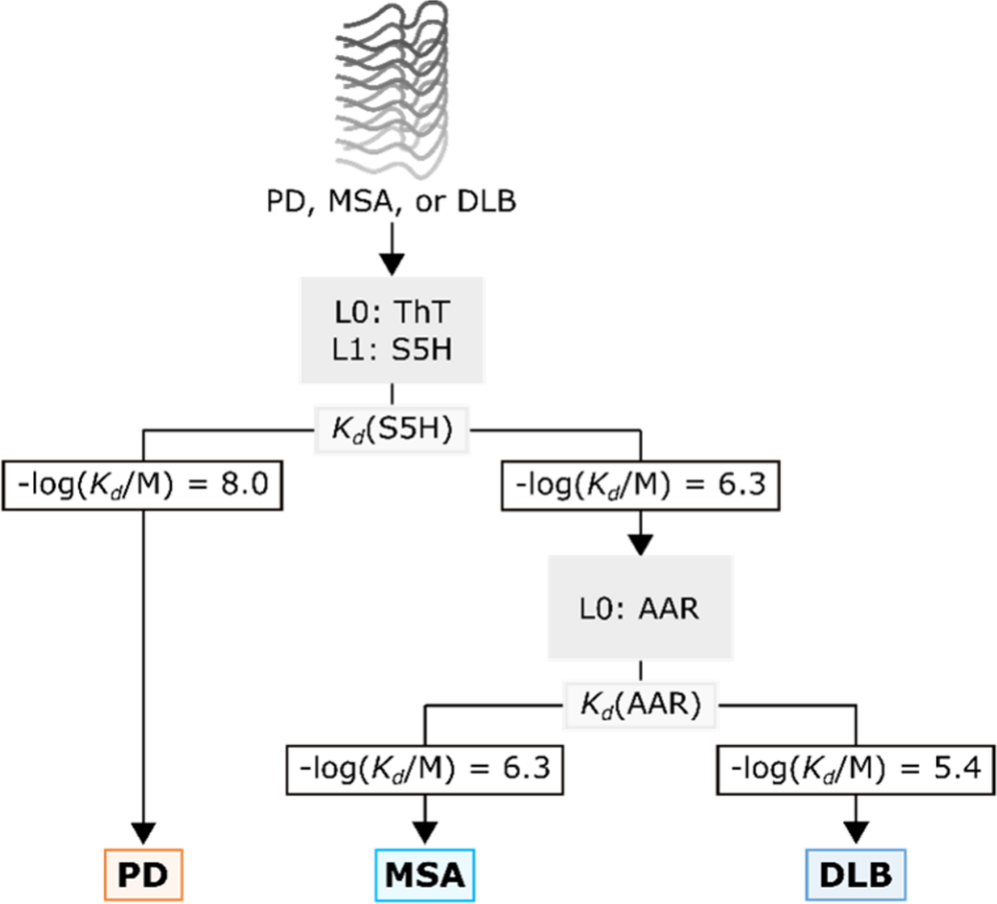
Decision tree for distinguishing PD, MSA, and DLB fibrils based on ligand dissociation constants (*K*_d_). The assays used are shown in gray boxes, and L0 and L1 indicate the role of the ligand in direct and competition binding assays as outlined in Figure 1.

The above assays can be used to construct decision trees that allow the eight different fibrils to be distinguished using just four experiments (Figures 5–7). The simplest method to differentiate fibrils is a qualitative binary classification of whether a ligand binds to that fibril or not. For a direct assay, binding is detected by a change in fluorescence properties of the reporter ligand (L0) upon addition to the fibril. For a competition assay, binding is detected by a change in fluorescence of the reporter ligand (L0) upon addition of a competing ligand (L1).

The decision tree outlined in Figure 5 allowed for five groups of fibrils to be identified using this binary classification methodology. Morphologies R and f91 can be identified by their inability to bind AAR. These two fibrillar polymorphs can then be distinguished as R does not bind S5H, whereas f91 does. Of the remaining fibrils that bind AAR, only the PMCA fibrils bind S5H, distinguishing them from the remaining de novo fibrils. Of these de novo fibrils, f65 and f110 bind BTA, but F does not. A binary classification using three different experiments therefore allows PMCA fibrils to be distinguished from de novo fibrils and allows all de novo fibrils to be uniquely identified except for f65 and f110.

Quantitative measurements can then be used to distinguish the remaining fibrils (Figure 6). Morphologies f65 and f110 are distinguished by the %BS1 measurements from an OXI competition assay, where more ThT was displaced from f65 than f110. For the PMCA fibrils, PD can be identified from the significantly lower value of *K*_d_ measured in an S5H competition assay (Figure 7). MSA and DLB fibrils can then be distinguished using the value of *K*_d_ measured in the AAR direct binding assay.

All eight fibril preparations can therefore be distinguished based on their unique ligand binding profiles in four different experiments. Additional assays using the other ligands in Figure 2 could also be used to provide further evidence in support of the conclusions from this minimal set of experiments (Figures S44−S54, Tables S5−S11). Notably, this result allows the pathological origin of the seeds used for each PMCA fibril morphology to be determined and the underlying disease to be identified.

## Conclusions

In summary, a selection of operationally simple binding assays have allowed eight αSyn fibril morphologies to be distinguished. A toolkit of six ligands (designated ThT, ThR, S5H, AAR, BTA, OXI) was prepared to target a range of fibril binding sites. These ligands were used in binding assays with different αSyn fibrils that had eight distinct structures and morphologies. Five of the fibrillar polymorphs were generated de novo (designated R, F, f91, f65, and f110). Three of the fibrillar polymorphs were generated using the PMCA process to aggregate recombinant αSyn seeded with brain homogenates of patients suffering from Parkinson’s disease, multiple system atrophy, and dementia with Lewy bodies (designated PD, MSA, and DLB).^30,31,39^ The morphologies of these PMCA fibrils have been shown to be determined by the seeding capabilities of the pathogenic αSyn aggregates present in the brain homogenates of the synucleinopathy patients, so the resulting fibril morphology reports on the disease state.

Marked differences in ligand selectivity, binding affinity, and population of binding sites were observed for different fibril morphologies. A binary classification based on whether specific ligands bound to a fibril or not was used to differentiate the fibrils into five groups: R bound neither S5H nor AAR; f91 bound S5H but not AAR; F bound AAR but neither S5H nor BTA; f65 and f110 both bound AAR and BTA but not S5H, and the PMCA fibrils PD, MSA, and DLB all bound AAR and S5H. Quantitative binding measurements were then used to differentiate the remaining fibrils. A competition assay with OXI displaced more ThT from f65 than f110. S5H was found to bind more strongly to PD fibrils than MSA or DLB fibrils, and AAR was shown to bind more strongly to MSA fibrils than DLB fibrils. In total, all eight fibril morphologies could be distinguished using just four binding assays and five different ligands. The ligand binding assay was capable of distinguishing the three different PMCA fibril morphologies obtained by seeding with brain homogenates from patients suffering from different synucleinopathies, which suggests that this approach could be used to identify different disease states.

The results demonstrate that screening a diverse toolkit of ligands can identify simple binding assays that differentiate fibril morphologies by reporting on structural differences. This approach is a generalizable method to identify experiments to differentiate any amyloid fibril based on differences in the structures and distribution of ligand binding sites. A key advantage here is that no direct structural information on the fibrils or their binding sites is required because the ligand binding profile is used to provide a characteristic fingerprint of the fibril morphology. Future work will focus on optimizing this methodology to rapidly screen binding assays that could be applied to fibril samples for the diagnosis of neurodegenerative diseases.

## Supporting Information Available

Preparation and characterization of compounds and protein aggregates; general procedures for binding assays; procedure for data fitting; binding assay data and results of fitting to binding isotherms; experimental details on the chemical synthesis of probes; preparation and characterization of αSyn fibrils; binding assay methods used; and titration data including quantitative binding measurements (PDF)
