# Supplementary material for: Ligand Profiling as a Diagnostic Tool to Differentiate Patient-Derived α-Synuclein Polymorphs
Source: ACS Chem Neurosci. 2024 May 1;15(10):2080–8. doi: 10.1021/acschemneuro.4c00178 (PMC11099917; doi:10.1021/acschemneuro.4c00178)
Supplement: Supplementary file 1 — cn4c00178_si_001.pdf [file cn4c00178_si_001.pdf]

Ligand Profiling as a Diagnostic Tool to Differentiate Patient-Derived α-Synuclein Polymorphs

Timothy S. Chisholm [a], Ronald Melki [b], Christopher A. Hunter [a,*]

[a] Yusuf Hamied Department of Chemistry, University of Cambridge, Lensfield Road, Cambridge CB2 1EW, UK.

[b] Institut François Jacob (MIRCen), CEA, CNRS, University Paris-Saclay, 18 Route du Panorama, 92260 Fontenay-aux-Roses, France.

* Email: herchelsmith.orgchem@ch.cam.ac.uk

Supporting Information

Contents

Materials and Instrumentation 4

Chemical Synthesis..5

Preparation of α-Synuclein Fibrils 29

F, R, f65, f91, and f110 *In Vitro* α-Synuclein Fibrils 29

PD, MSA, and DLB PMCA α-Synuclein Fibrils...30

Biophysical Characterisation of α-Synuclein Fibrils 31

Circular Dichroism Spectra 31

Transmission Electron Microscopy 32

Limited Proteolysis...33

UV-Visible Characterisation 35

Fluorescence Characterisation 39

Dilution Series 46

General Procedures for Binding Assays 51

Fluorescence Titrations...51

Saturation Binding Assays...51

Fluorescence Anisotropy Binding Assays...51

One-Step Competition Binding Assays...51

Two-Step Competition Binding Assays 51

Binding Assays Performed 52

Data Fitting 53

Saturation Binding Assays...54

ThT (L0) 54

AAR (L0)...55

BTA (L0) 56

Dissociation Constants..57

One-Step Competition Binding Assays 58

BTA (L1) into ThT (L0) 58

OXI (L1) into ThT (L0) 59

ThR (L1) into ThT (L0) 60

S5H (L1) into ThT (L0) 61

S5H (L1) into ThT (L0), then ThR (L2) 62

Dissociation Constants and %BS1 65

One-Step Competition Binding Assays with Two Fluorescent Ligands 67

ThT (L1) into AAR (L0) 67

Dissociation Constants and %BS1 68

References 69

Materials and Instrumentation

All solvents and chemicals were obtained from commercial sources and used without further purification unless otherwise stated. The ligands screened in Chapter 3 were purchased from Enamine, Ukraine. Reactions were monitored by TLC or LCMS. TLC analyses were performed on Merck TLC Silica gel 60 F_{254} glass plates (0.2 mm). LCMS analyses of samples were performed using a Waters Acquity H-class UPLC coupled with a single quadrupole Waters SQD2. An Acquity UPLC CSH C18 Column, 130Å, 1.7 µm, 2.1 mm x 50 mm was used as the UPLC column.

Purification of compounds by silica column chromatography were performed using an automated system (Combiflash® Rf+ or Combiflash® Rf+ Lumen) with prepackaged silica cartridges (25 µm or 50 µm PuriFlash® columns). ^{1}H and ^{13}C NMR spectra were recorded using a Bruker 600 MHz Avance 600 BBI spectrometer, a 500 MHZ Acance III Smart Probe spectrometer, or a 400 MHz Avance III HD Smart Probe spectrometer at 290.0 ± 0.1 K. Residual solvent peaks were used as an internal standard for calibration. All chemical shifts are quoted in ppm on the δ scale and the coupling constants are expressed in Hz. Signal splitting patterns are described as a singlet (s), broad singlet (br s), doublet (d), triplet (t), quartet (q), or multiplet (m).

HPLC-MS and HPLC-MS/MS analysis was performed on an Agilent 1100 Series LC system equipped with a G1310A isocratic pump, G1314A variable wavelength detector, G1316A thermostatted column compartment, and an Agilent 6300 Series Ion Trap. UV-vis spectra were collected on an Agilent Cary 60 UV-vis spectrophotometer controlled by Cary WinUV software. Fluorescence spectroscopic data were recorded using an Agilent Cary Eclipse Fluorescence Spectrophotometer controlled by Cary WinUV software, and equipped with a Cary Eclipse Automated Polarizer for anisotropy measurements. FT-IR spectra were collected with an ALPHA FT-IR Spectrometer from Bruker. Melting points were recorded with a Mettler Toledo MP90 melting point apparatus.

Protein LoBind (Eppendorf) microtubes were used for preparing and storing all solutions containing protein. Low retention pipette tips were used for all aqueous fluid handling.

Chemical Synthesis

The preparation of ThT, OXI, S5H, and BTA has been previously reported.[1]

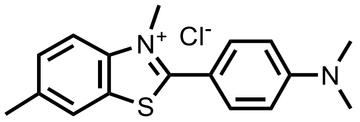

2-(4-(dimethylamino)phenyl)-3,6-dimethylbenzo[d]thiazol-3-ium chloride (Thioflavin T, ThT)

Thioflavin T (1.13 g, 3.54 mmol) was purchased from Sigma Aldrich with dye content ≥65%. Thioflavin T was recrystallised twice from hot water prior to use (0.53 g, 1.7 mmol, 47%).

Aspect: yellow crystalline solid. ***Yield***: 0.53 g (47%).

***^{1}H NMR (400 MHz, $CDCl_3$), δ(ppm)**:* 8.05 – 7.97 (m, 2H), 7.86 (d, *J* = 8.5 Hz, 2H), 7.53 (d, *J* = 8.5 Hz, 1H), 6.82 (d, *J* = 8.4 Hz, 2H), 4.55 (s, 3H), 3.12 (s, 6H), 2.49 (s, 3H).

^{13}C NMR (101 MHz, $CDCl_3$) δ(ppm): 172.57, 163.88, 154.15, 141.00, 139.09, 132.78, 131.27, 128.25, 123.44, 116.73, 112.40, 111.00, 77.48, 77.16, 76.84, 40.28, 39.65, 21.64.

HRMS (ESI+): 283.1265 m/z: Calculated for $C_{17}H_{19}N_2S^+$ = 283.1269 $[M]^+$.

IR (ATR, cm^{-1}): 3403, 1604, 1501, 1480, 1441, 1387, 1350, 1233, 1212, 1158, 827.

MP: 195.5-196.5 °C

Characterisation data is in agreement with that reported by Chisholm *et al.*[1]

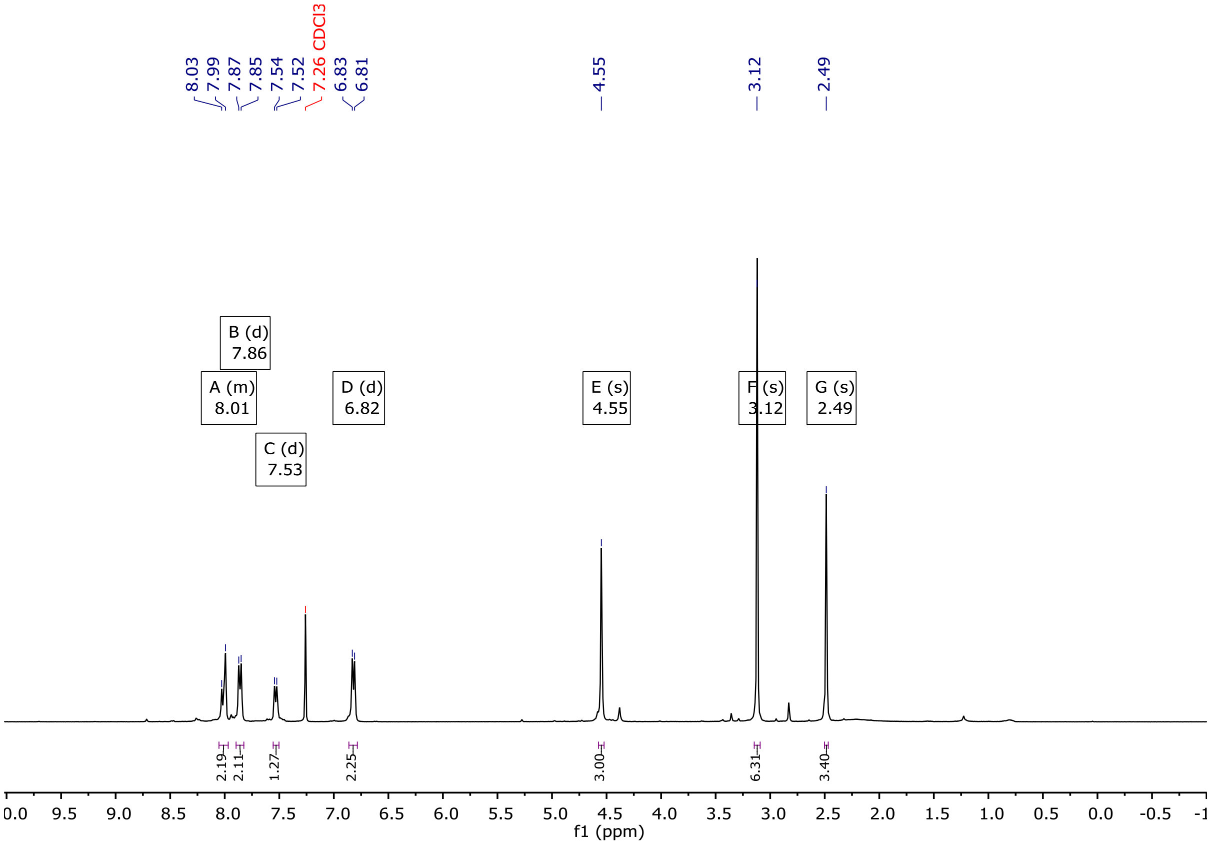

Figure S1. ^{1}H NMR (400 MHz, $CDCl_3$) spectra of ThT.

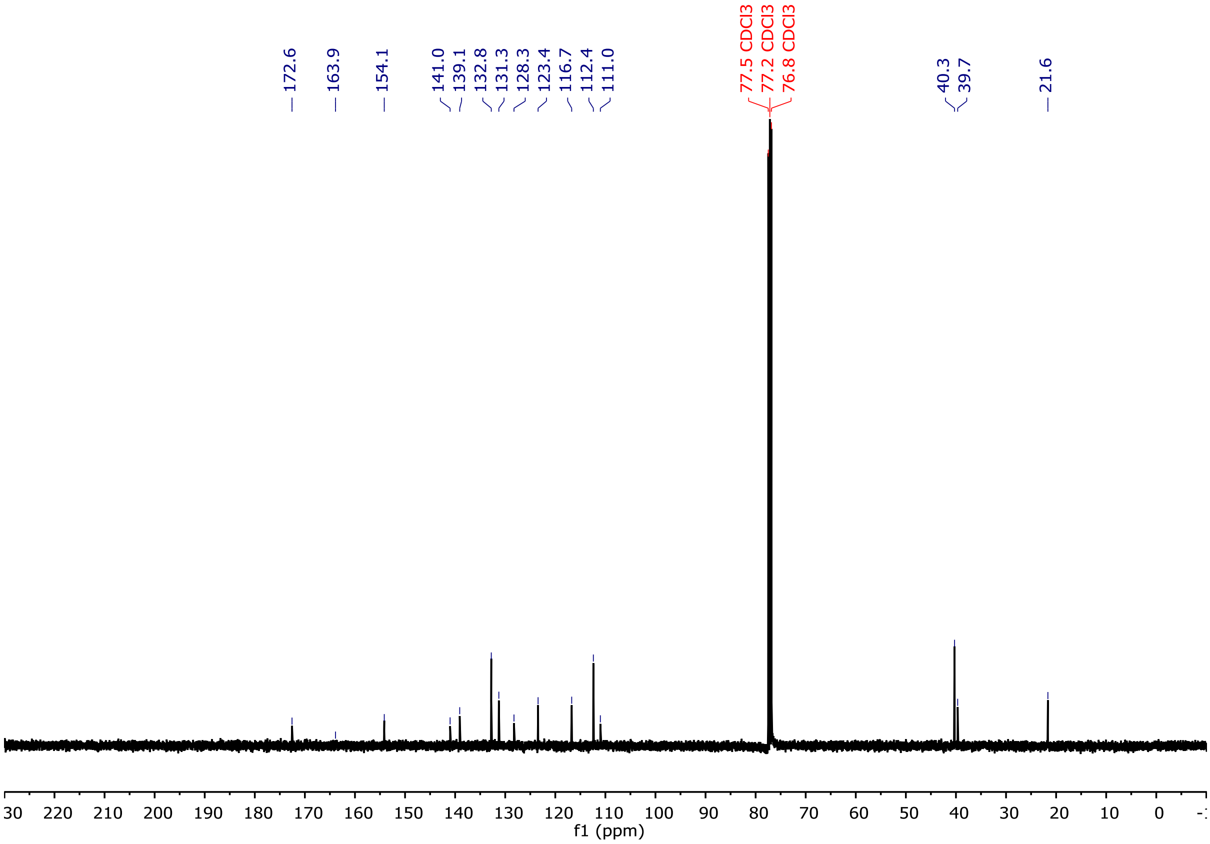

Figure S2. ^{13}C (101 MHz, $CDCl_3$) NMR spectra of ThT.

(Z)-3-((E)-3-(4-nitrophenyl)allylidene)indolin-2-one (OXI)

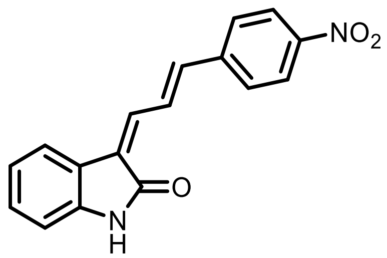

OXI was synthesised following a previously reported procedure.[1] A solution of 2-oxindole (3.61 g, 10 mmol) and 4-nitrocinnamaldehyde (1.77 g, 10 mmol, 1.0 equiv.) in acetic acid (25 mL, 2.5 mL/mmol) and 37% HCl (0.5 mL, 0.05 mL/mmol) was heated to reflux for 3 h. The resultant red solution was cooled to room temperature and diluted with distilled water (25 mL). The red precipitate was isolated by filtration and purified using silica column chromatography (CH_2Cl_2 to CH_2Cl_2:EtOAc 95:5) to afford OXI as a red crystalline solid (1.36 g, 4.65 mmol, 47%).

Aspect: red solid. ***Yield***: 1.36 g (47%).

^{1}H NMR (400 MHz, CDCl$_3$), δ(ppm): 10.60 (s, 1H), 8.63 (dd, J = 15.8, 11.5 Hz, 1H), 8.27 (d, J = 8.8 Hz, 2H), 7.82 (d, J = 8.8 Hz, 2H), 7.61 (s, 1H), 7.59 (d, J = 5.2 Hz, 1H), 7.28 (d, J = 15.7 Hz, 1H), 7.22 (t, J = 7.2 Hz, 1H), 6.97 (t, J = 7.6 Hz, 1H), 6.82 (d, J = 7.7 Hz, 1H).

^{13}C NMR (101 MHz, CDCl$_3$) δ(ppm): 168.13, 147.04, 142.70, 139.09, 134.14, 129.71, 128.16, 127.56, 124.29, 123.31, 121.29, 120.46, 109.67, 40.15, 39.94, 39.73, 39.52, 39.31, 39.10, 38.89.

HRMS (ESI+): 293.0921 m/z: Calculated for $C_{17}H_{13}N_2O_3^+$ = 293.0926 $[M+H]^+$.

IR (ATR, cm^{-1}): 2924, 2849, 2361, 2334, 1691, 1603, 1585, 1548, 1510, 1468, 1339, 1214, 1180, 1109, 978, 869, 838.

Decomposition point: decolorisation at 254 °C, melting at 260.2-261.2 °C with effervescence.

Characterisation data is in agreement with that reported by Chu *et al.*[2]

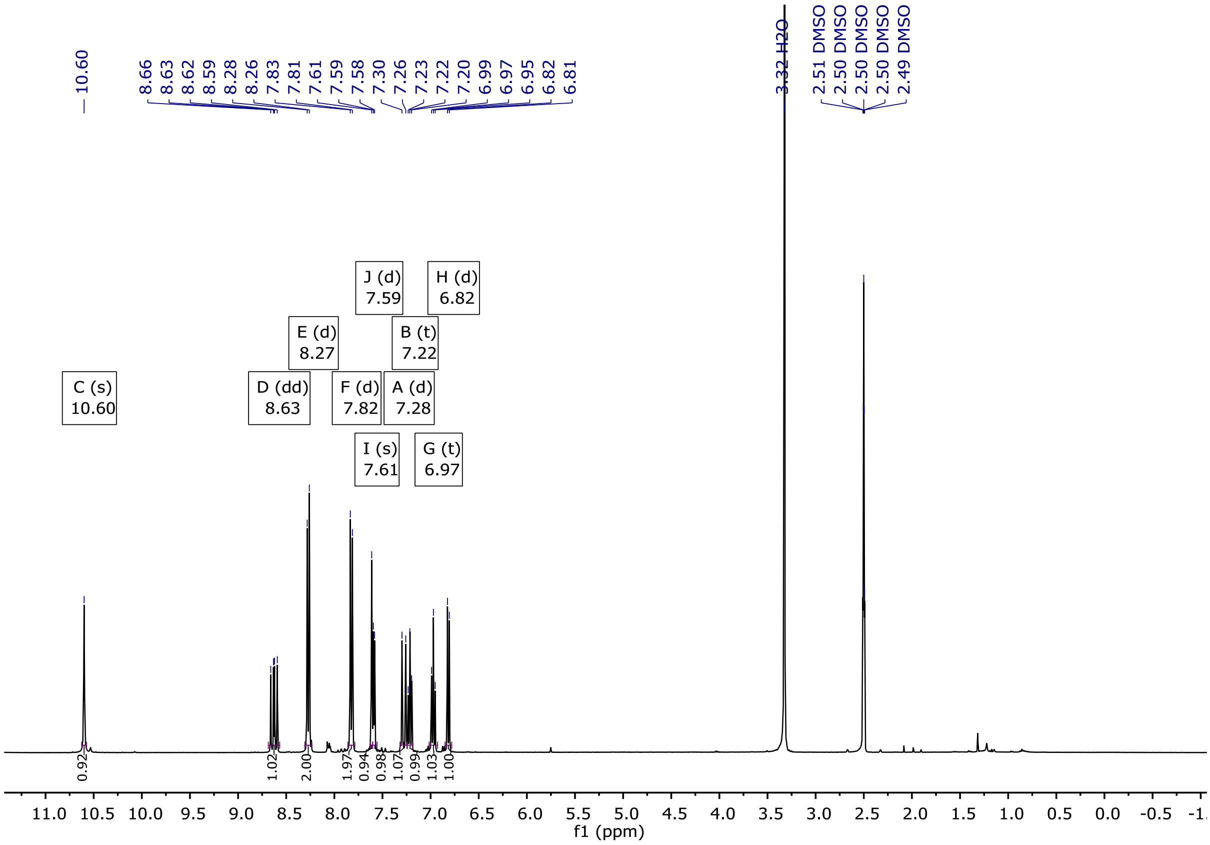

Figure S3. ^{1}H NMR (400 MHz, $CDCl_3$) spectra of OXI.

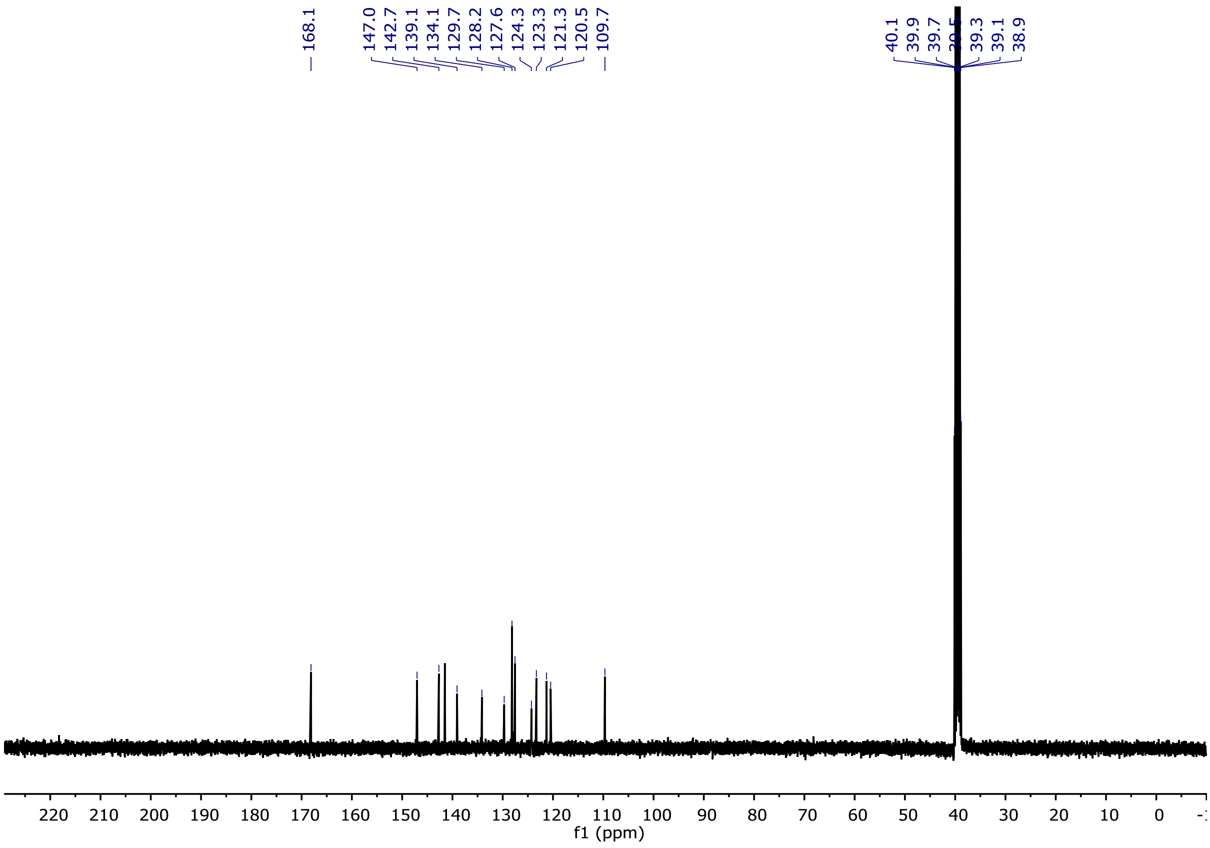

Figure S4. ^{13}C NMR (101 MHz, $CDCl_3$) spectra of OXI.

(*E*)-3-(4-(benzyloxy)phenyl)acrylic acid (S01)

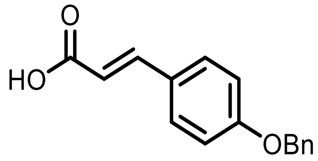

To coumaric acid (1.31 g, 7.98 mmol) in absolute ethanol (8.0 mL) was added benzyl bromide (1.96 mL, 16.5 mmol) then sodium hydroxide (40 g, 39.8 mmol) in distilled water (8.0 mL). The resultant yellow suspension was stirred for 5 min until clear, then refluxed for 36 h. The reaction was then cooled in an ice bath and poured into ice cold water (50 mL) and quenched with 5 M HCl. The resultant precipitate was isolated by filtration, washed with ice cold water (3 x 10 mL) then dried *in vacuo* to afford (*E*)-3-(4-(benzyloxy)phenyl)acrylic acid **S01** as a white solid (2.03 g, 7.98 mmol, 100%).

Aspect: yellow solid. ***Yield***: 2.03 g (100%).

^{1}H NMR (400 MHz, $CDCl_3$), δ(ppm): 7.57 (d, J = 8.3 Hz, 2H), 7.48 – 7.31 (m, 6H), 7.02 (d, J = 8.2 Hz, 2H), 6.36 (d, J = 16.0 Hz, 1H), 5.15 (s, 2H).

HRMS (ESI+): 255.1010 m/z: Calculated for $C_{16}H_{15}O_3^+$ = 255.1016 $[M+H]^+$.

IR (ATR, cm^{-1}): 2911, 1669, 1601, 1544, 1510, 1427, 1413, 1304, 1289, 1241, 1172, 1014, 981, 968, 920, 825.

MP: 205.9-206.9 °C

Characterisation data is in agreement with that reported by Lee *et al.*[3]

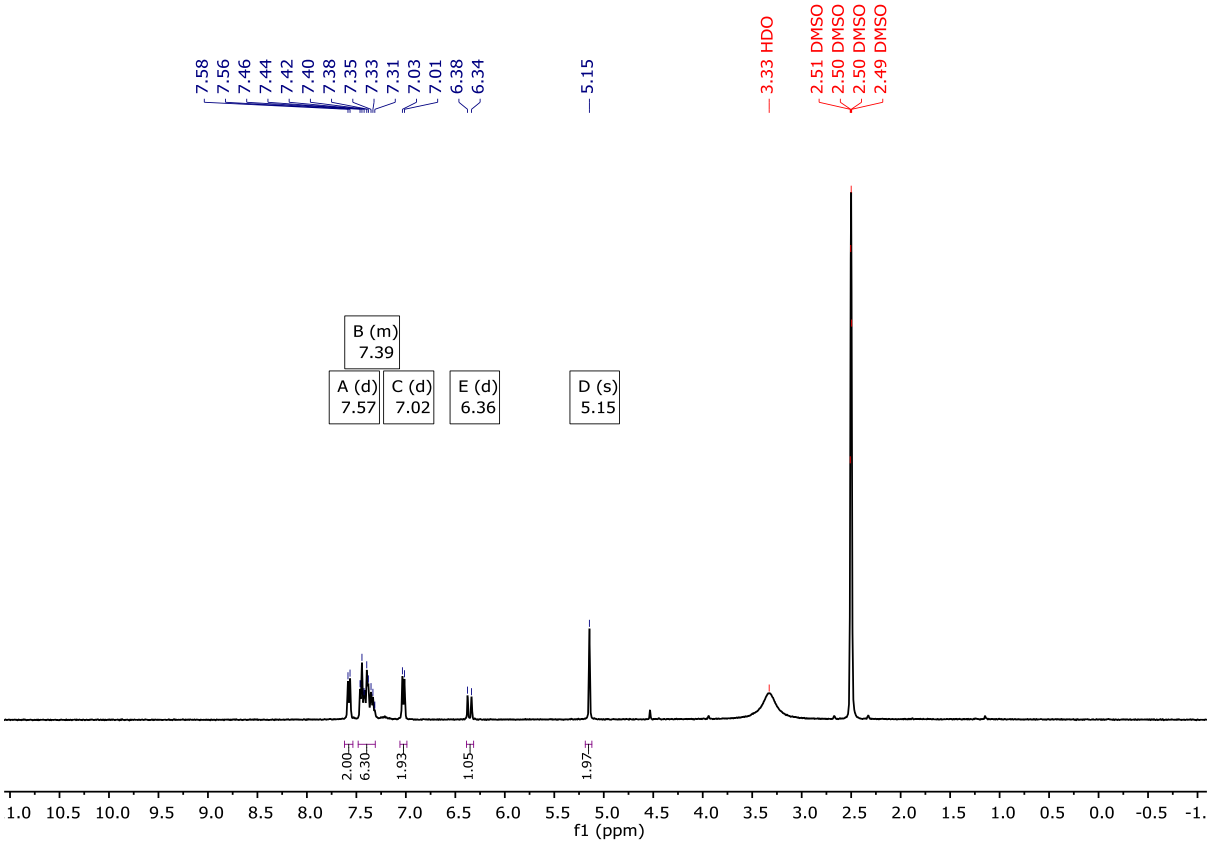

Figure S5. ^{1}H NMR (400 MHz, $CDCl_3$) spectra of **S01**.

(E)-3-(4-(benzyloxy)phenyl)acryloyl chloride (S02)

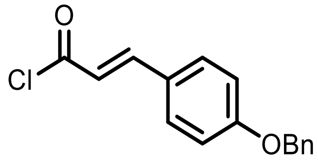

S01 (2.03 g, 7.98 mmol) was solvated in oxalyl chloride (70 mL, 8.8 mL/mmol) at 0 °C, then warmed to room temperature and stirred for 2 h. The excess oxalyl chloride was removed *in vacuo* to afford **S02** as a white solid (2.02 g, 7.41 mmol, 93%).

Aspect: yellow solid. ***Yield***: 2.02 g (93%).

^{1}H NMR (400 MHz, CDCl$_3$), δ(ppm): 7.79 (d, *J* = 15.4 Hz, 1H), 7.54 (d, *J* = 8.4 Hz, 2H), 7.45 – 7.34 (m, 5H), 7.02 (d, *J* = 8.4 Hz, 2H), 6.51 (d, *J* = 15.5 Hz, 1H), 5.13 (s, 2H).

^{13}C NMR (101 MHz, CDCl$_3$) δ(ppm): 166.3, 162.1, 150.6, 146.7, 136.2, 131.3, 130.2, 128.9, 128.8, 128.5, 128.5, 127.6, 126.2, 119.9, 115.7, 77.5, 77.2, 76.8, 70.4.

HRMS (ESI+): 273.0674 m/z: Calculated for $C_{16}H_{14}ClO_2^+$ = 273.0677 $[M+H]^+$.

Characterisation data is in agreement with that reported by Lee *et al.*[3]

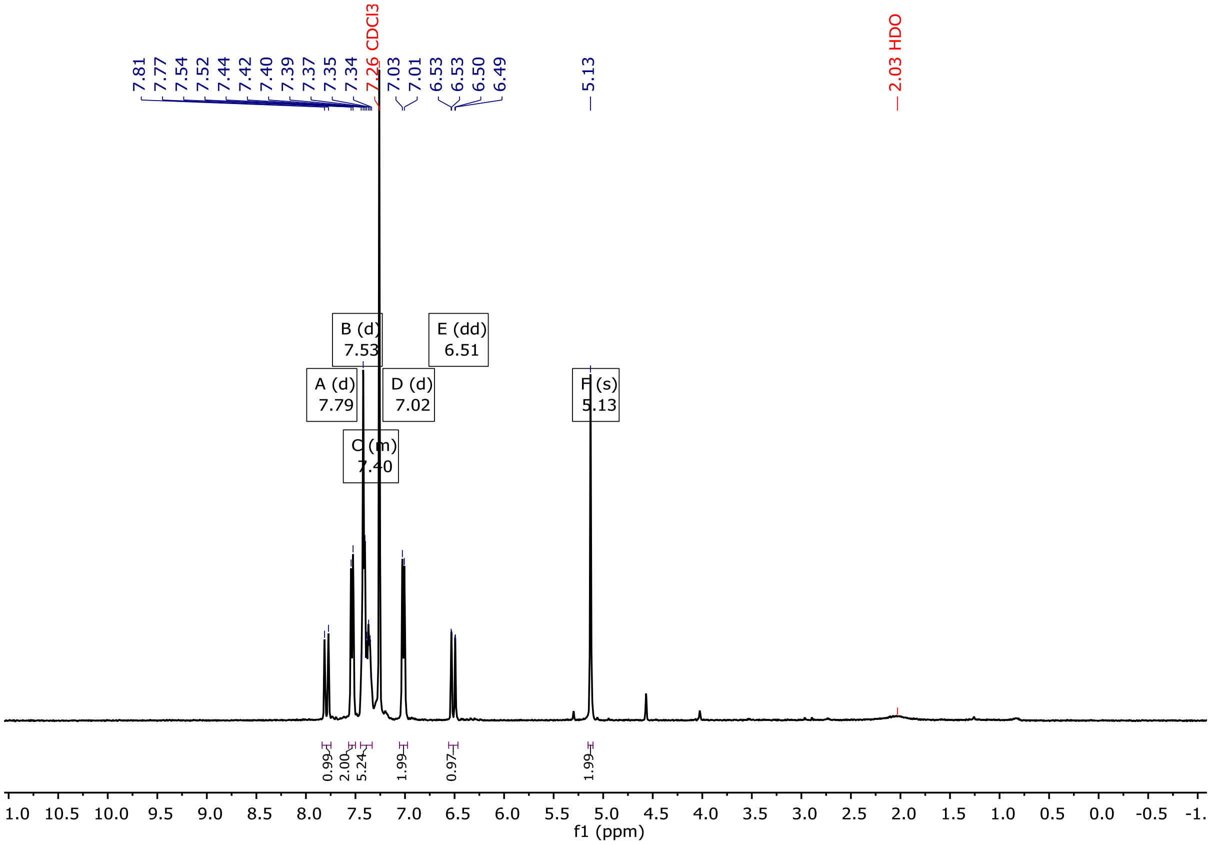

Figure S6. ^{1}H NMR (400 MHz, $CDCl_3$) spectra of **S02**.

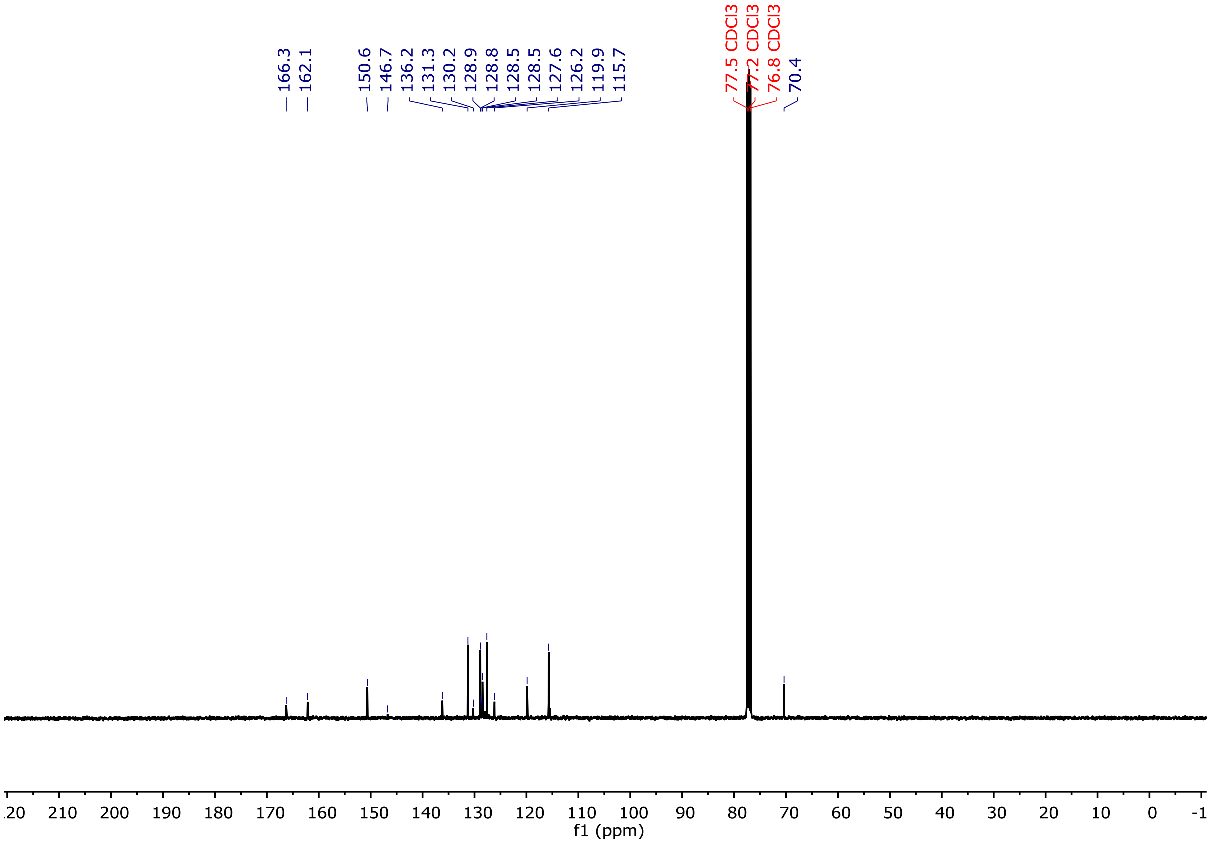

Figure S7. ^{13}C NMR (101 MHz, $CDCl_3$) spectra of **S02**.

(2Z,4E)-5-(4-(benzyloxy)phenyl)-3-hydroxy-1-(thiazol-2-yl)penta-2,4-dien-1-one (S03)

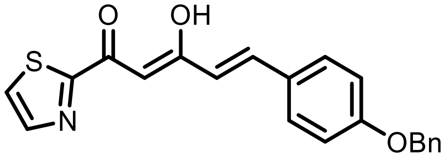

To a solution of 1-(thiazol-2-yl)ethan-1-one (340 µL, 3.3 mmol, 1.0 equiv.) in THF (10 mL) at -78 °C was added 0.94 M lithium bis(trimethylsilyl)amide in THF (LiHMDS, 3.9 mL, 3.63 mmol, 1.1 eq.) dropwise. The resultant solution was stirred at -78 °C for 1 h. To the reaction mixture was then added **S02** (990 mg, 3.63 mmol, 1.2 equiv.) in THF (10 mL) dropwise, and the reaction was stirred for a further 3 h at -78 °C. The reaction mixture was then warmed to room temperature and diluted with saturated NH_4Cl (20 mL) then extracted with EtOAc (2 x 20 mL). The combined organic extracts were washed with distilled H_2O (20 mL), dried over anhydrous sodium sulfate, and concentrated *in vacuo*. The crude product was purified using silica column chromatography (PE to PE:EtOAc 3:1) to afford **S03** as a yellow solid (350 mg, 0.96 mmol, 30%).

Aspect: yellow solid. ***Yield***: 0.35 g (30%).

^{1}H NMR (400 MHz, CDCl$_3$), δ(ppm): 15.03 (s, 1H), 8.00 (d, *J* = 3.1 Hz, 1H), 7.66 – 7.60 (m, 2H), 7.50 (d, *J* = 8.7 Hz, 2H), 7.40 (td, *J* = 16.3, 15.1, 7.4 Hz, 7H), 6.99 (d, *J* = 8.7 Hz, 2H), 6.74 (s, 1H), 6.51 (d, *J* = 15.8 Hz, 1H), 5.09 (s, 2H).

^{13}C NMR (101 MHz, CDCl$_3$) δ(ppm): 182.4, 178.2, 167.3, 160.7, 144.8, 140.6, 136.4, 132.5, 129.9, 128.7, 128.2, 127.8, 127.8, 127.5, 127.5, 125.3, 120.0, 115.4, 114.3, 96.9, 77.4, 77.1, 76.8, 70.1.

HRMS (ESI+): 361.1023 m/z: Calculated for $C_{17}H_{13}N_2OS^+$ = 293.0926 $[M+H]^+$.

IR (ATR, cm^{-1}): 3110, 3086, 3065, 3033, 2923, 2853, 1628, 1562, 1509, 1481, 1437, 1422, 1383, 1330, 1311, 1283, 1244, 1164, 1114, 1079, 1060, 1036, 1026, 972, 958, 872, 825.

MP: 112.3-117.8 °C

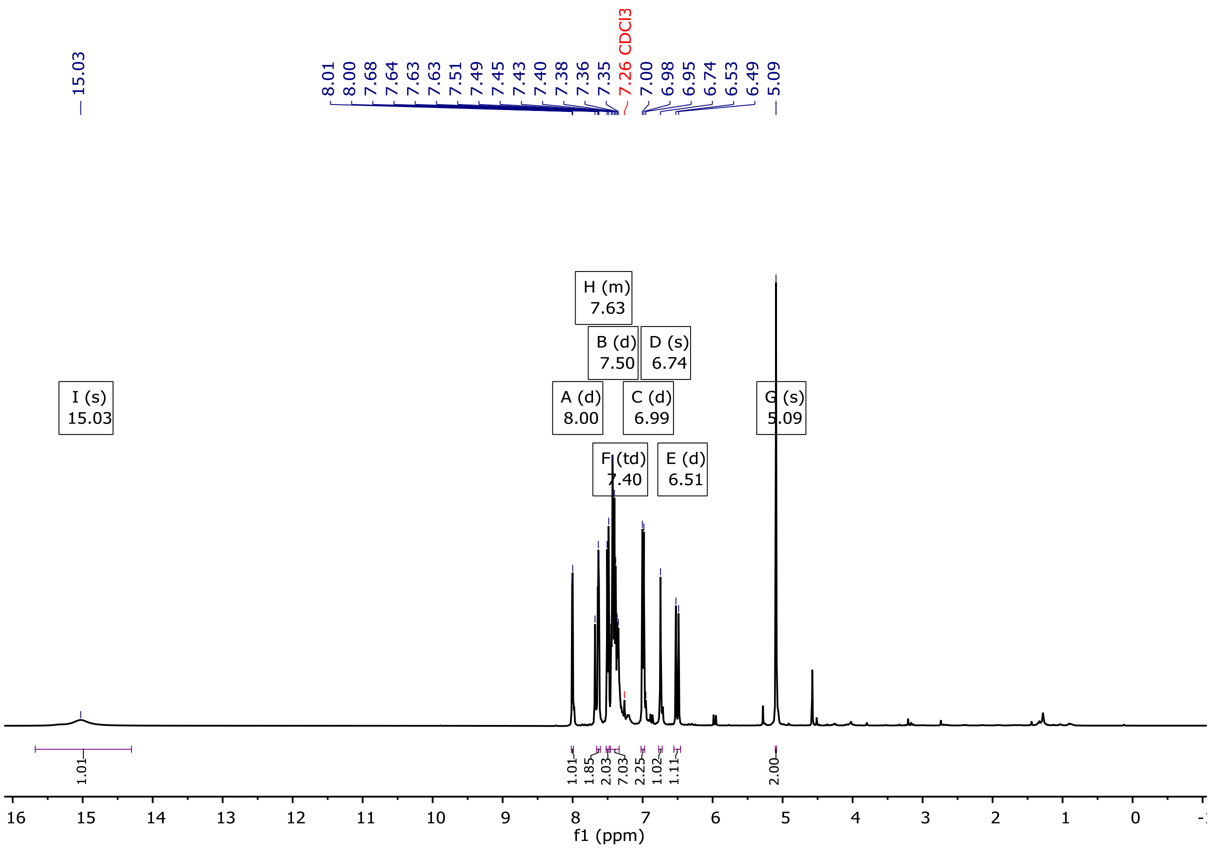

Figure S8. ^{1}H NMR (400 MHz, $CDCl_3$) spectra of **S03**.

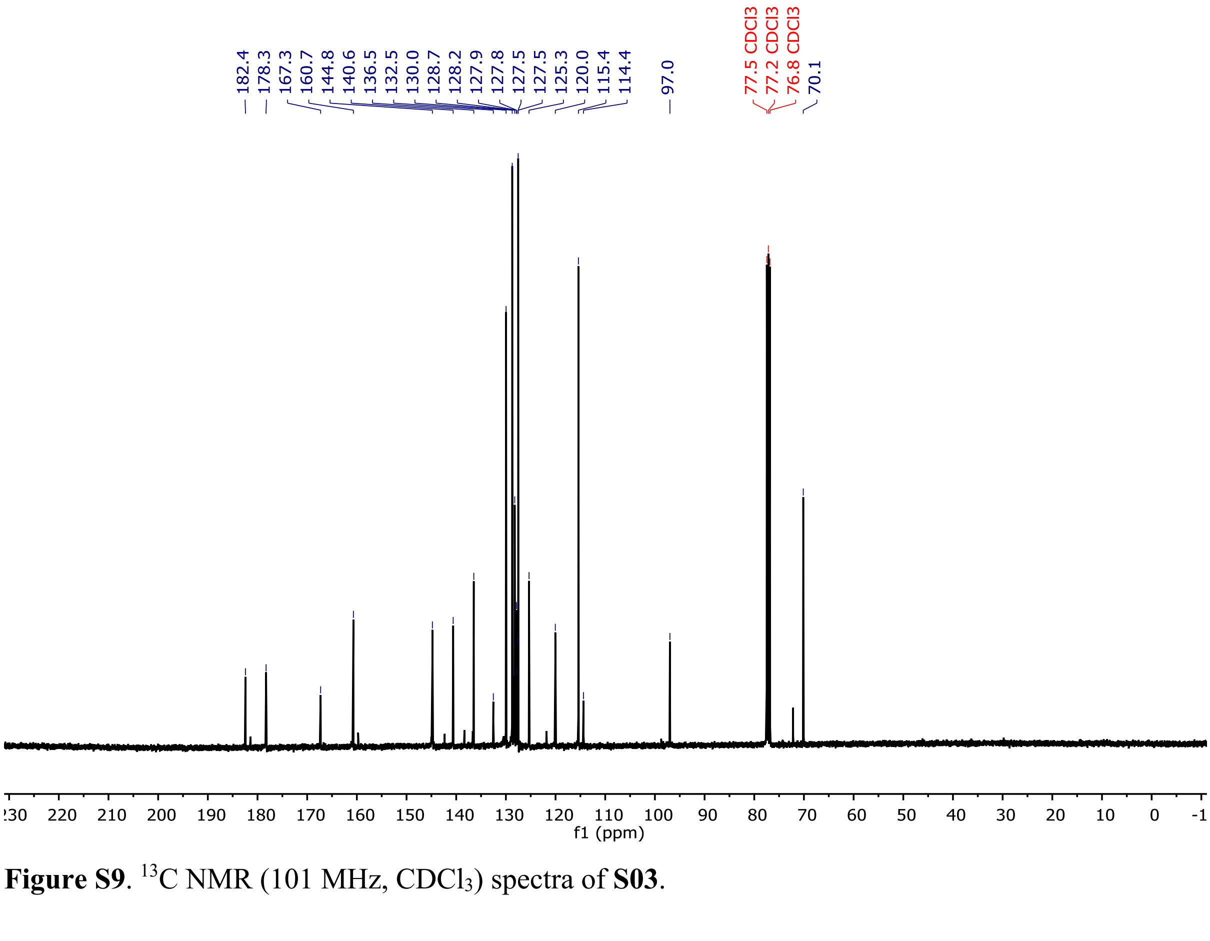

Figure S9. ^{13}C NMR (101 MHz, $CDCl_3$) spectra of **S03**.

(E)-5-(4-(benzyloxy)styryl)-3-(thiazol-2-yl)isoxazole (S5H)

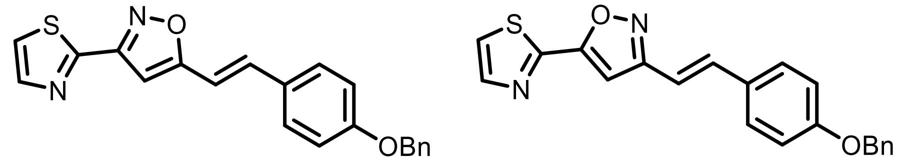

S5H was synthesised based on a previously reported procedure.[3] To a suspension of **S03** (255 mg, 0.700 mmol) in EtOH (4 mL, 5.7 mmol/mL) was added $NH_2OH{\cdot}HCl$ (116 mg, 196 µL, 2.80 mmol, 4.0 equiv.). The mixture was stirred at 80 °C for 6 h, then cooled and the solvent removed *in vacuo*. The crude product was purified using silica column chromatography (PE to PE:EtOAc 3:1) to afford an approximately 1:1 mixture of two isomers S5H as a crystalline yellow solid (181 mg, 0.502 mmol, 72%). This mixture was used without further purification as previously reported.[1,4]

Aspect: yellow oil. ***Yield***: 181 mg (72%).

^{1}H NMR (400 MHz, $CDCl_3$), δ(ppm): 7.98 (dd, J = 12.6, 3.2 Hz, 2H), 7.56 – 7.30 (m, 17H), 7.20 (d, J = 16.4 Hz, 1H), 7.03 (dd, J = 26.8, 8.6 Hz, 6H), 6.88 (d, J = 16.4 Hz, 1H), 6.80 (s, 1H), 5.11 (s, 4H).

^{13}C NMR (101 MHz, $CDCl_3$), δ(ppm) 170.2, 163.8, 162.9, 160.0, 159.8, 158.8, 156.9, 154.5, 144.6, 143.8, 136.7, 136.7, 136.5, 135.4, 128.9, 128.8, 128.7, 128.4, 128.3, 128.2, 127.6, 121.5, 121.0, 115.4, 115.4, 113.3, 110.7, 99.0, 98.9, 77.5, 77.2, 76.8, 70.2.

HRMS (ESI+): 361.1023 m/z: Calculated for $C_{21}H_{17}N_2O_2S^+$ = 361.1011 $[M+H]^+$.

IR (ATR, cm^{-1}): 3107, 2921, 2853, 1643, 1603, 1579, 1514, 1454, 1430, 1383, 1316, 1301, 1254, 1173, 1157, 1140, 1110, 1079, 1060, 1037, 1028, 987, 962, 917, 872, 818.

MP: 142.2-143.7 °C

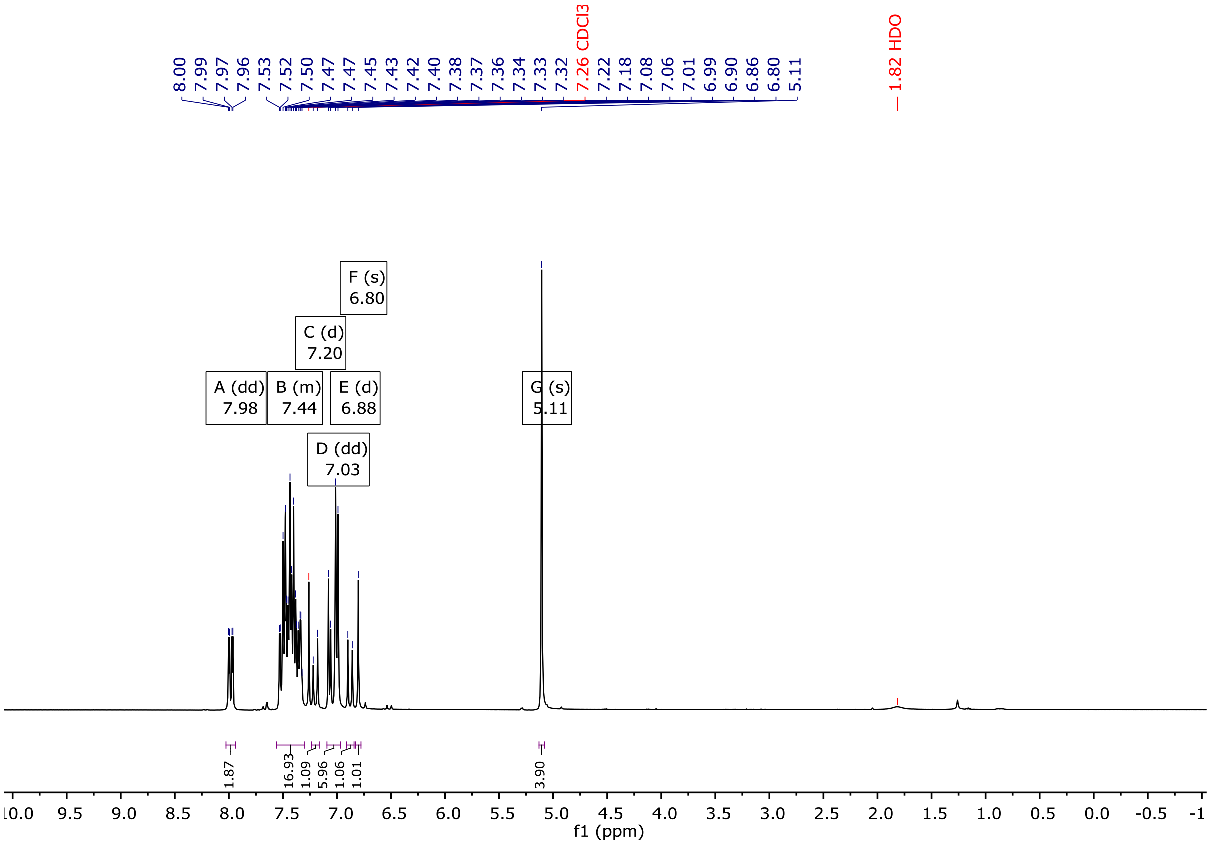

Figure S10. ^{1}H NMR (400 MHz, $CDCl_3$) spectra of S5H.

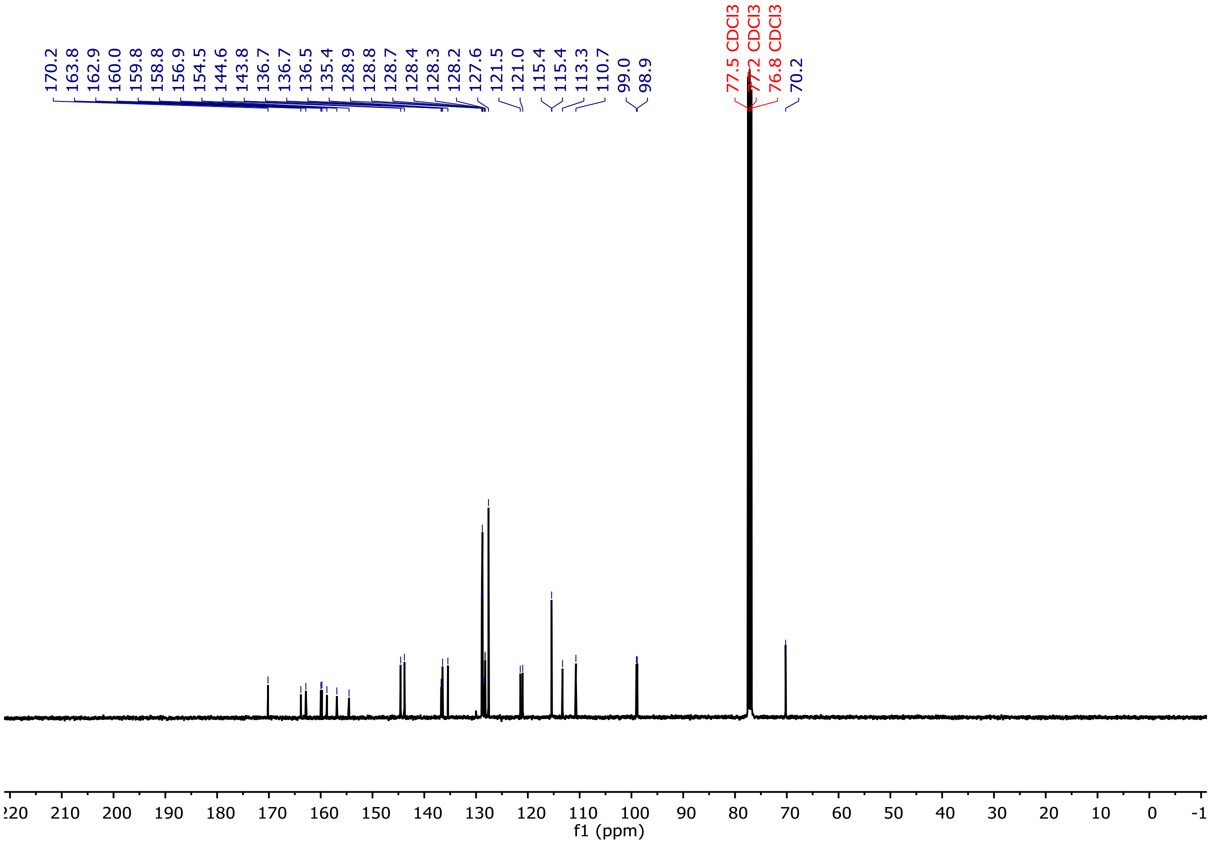

Figure S11. ^{13}C NMR (101 MHz, $CDCl_3$) spectra of S5H.

2-amino-5-methoxybenzenethiol (S04)

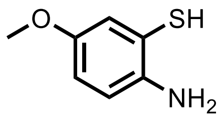

S04 was synthesised following a previously reported procedure.[4] To a suspension of 2-amino-6-methoxybenzothiazole (20.2 g, 112 mmol) and potassium hydroxide (62.3 g, 1.11 mol) in degassed water (250 mL) was added ethyleneglycol (27.9 mL). This clear solution was refluxed for 21 h, and the resultant dark green solution was cooled to room temperature and neutralised with acetic acid. The formed precipitate was collected using vacuum filtration and washed with cold water (200 mL) then dried *in vacuo* to afford **S04** as a brown solid (17.4 g, 112 mmol, 100%).

Aspect: brown solid. ***Yield***: 17.4 g (100%).

^{1}H NMR (400 MHz, DMSO-d^{6}), δ(ppm): 6.83 (dd, *J* = 8.7, 3.0 Hz, 1H), 6.71 (d, *J* = 8.7 Hz, 2H), 3.63 (s, 3H).

^{13}C NMR (101 MHz, DMSO-d^{6}), δ(ppm): 152.0, 142.7, 120.2, 119.5, 119.4, 116.8, 55.90.

HRMS (ESI+): 155.0407, 309.0718 m/z: Calculated for $C_{14}H_{18}N_2O_2S_2^{2+}$ = 155.0400 [disulfide+2H]$^{2+}$, $C_{14}H_{17}N_2O_2S_2^{+}$ = 309.0726 [disulfide+H]$^{+}$

FT-IR (ATR): 3444, 3351, 2927, 2831, 1595, 1490, 1268, 1035, 816.

Decomposition point: decolorisation at 57 °C, turned black at 75 °C, melted at 78.0-79.0 °C.

Characterisation data is in agreement with that reported by Qin *et al.*[5]

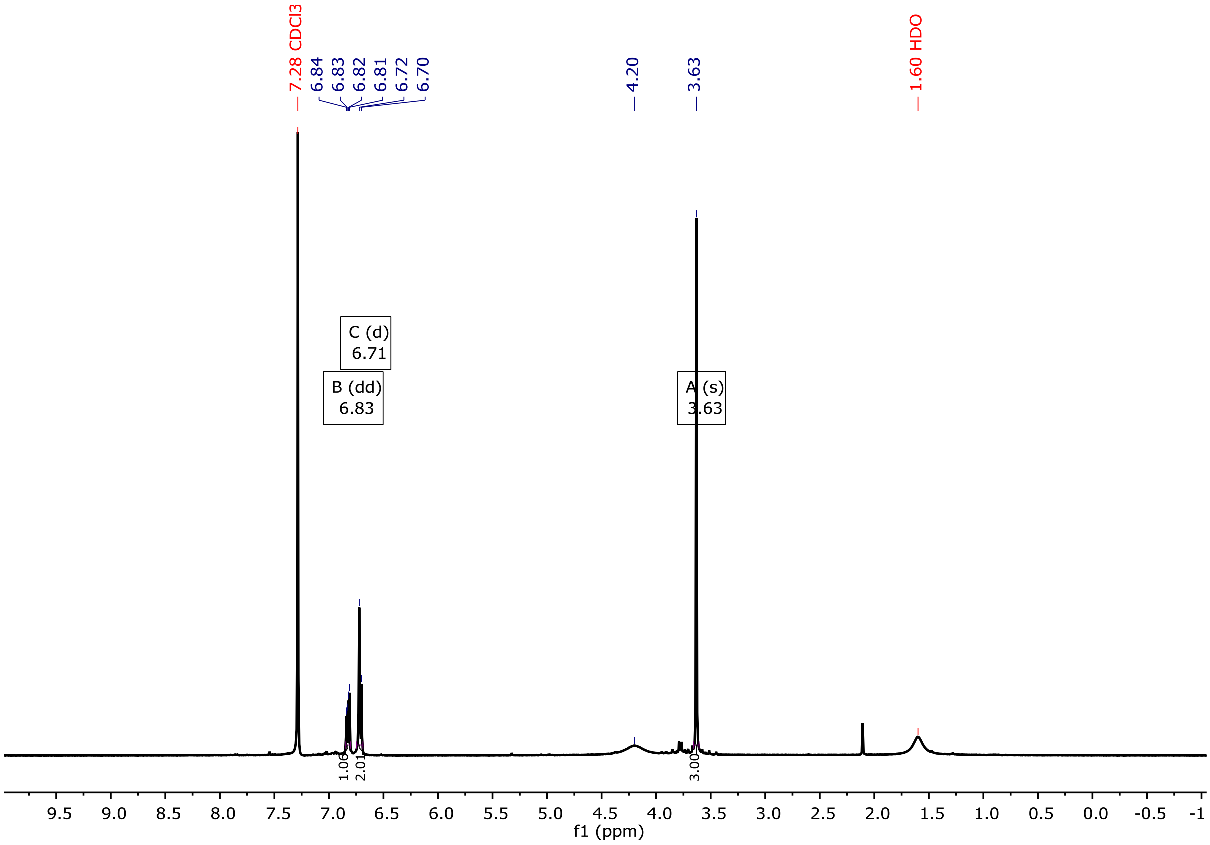

Figure S12. ^{1}H NMR (400 MHz, DMSO-d^{6}) spectra of **S04**.

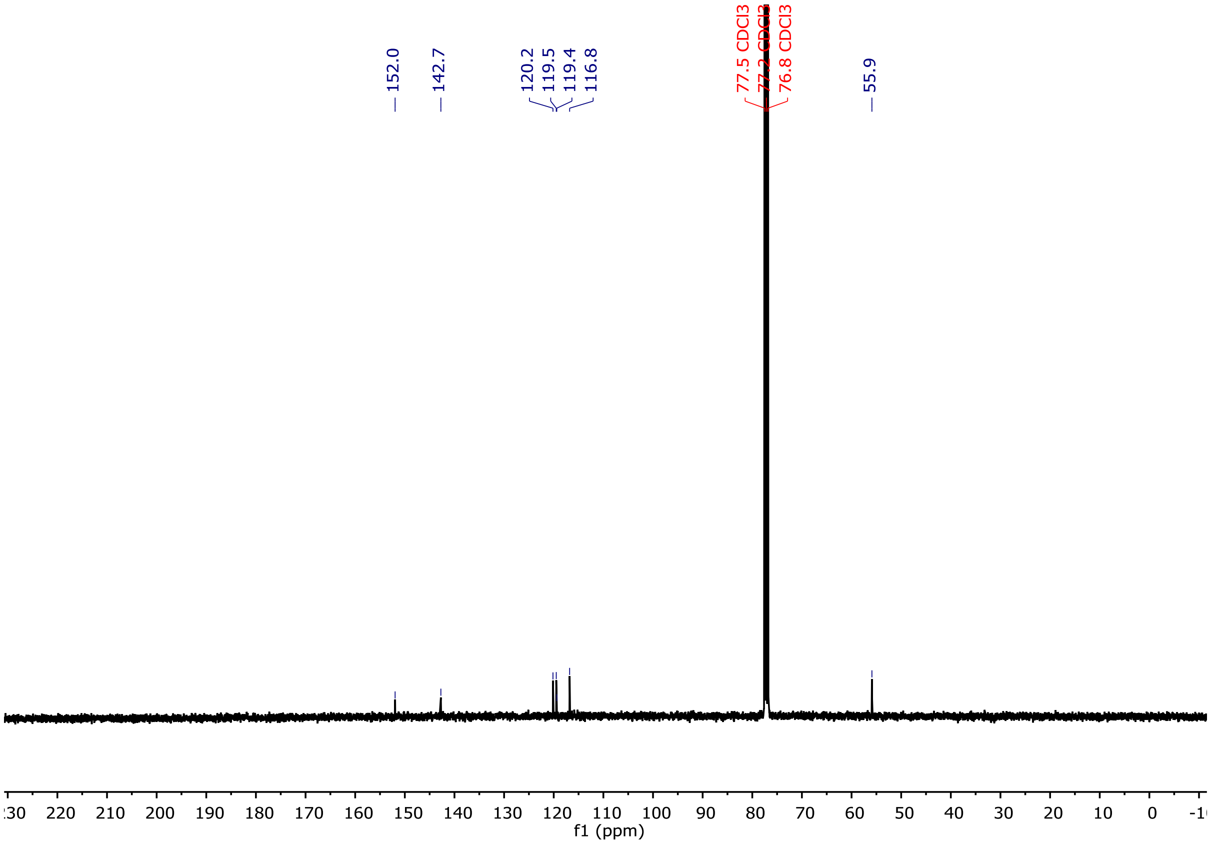

Figure S13. ^{13}C NMR (101 MHz, DMSO-d^{6}) spectra of **S04**.

4-(6-methoxybenzo[*d*]thiazol-2-yl)-N,N-dimethylaniline (S05)

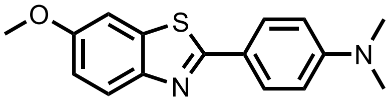

S05 was synthesised following a previously reported procedure.[4] To a solution of **S04** (17.3 g, 111 mmol) in DMSO (200 mL) was added 4-(dimethylamino)-benzaldehyde (82.8 g, 555 mmol) and the dark green solution stirred at 180 °C for 20 min. The reaction mixture was cooled to room temperature and a mixture of ethylacetate:water (1:1, 200 mL) was added. The resultant precipitate was collected by vacuum filtration and washed with water (350 mL) then ethanol (450 mL) then dried *in vacuo* to afford **S05** as a yellow solid (20.8 g, 73.3 mmol, 64%).

Aspect: yellow solid. ***Yield***: 20.8 g (64%).

^{1}H NMR (400 MHz, DMSO-d^6), δ(ppm)*:* 7.82 (t, *J* = 7.9 Hz, 3H), 7.62 (s, 1H), 7.06 (d, *J* = 8.6 Hz, 1H), 6.81 (d, *J* = 8.1 Hz, 2H), 3.83 (s, 3H), 3.01 (s, 6H).

^{13}C NMR (101 MHz, DMSO-d^6), δ(ppm)*:* 165.8, 157.3, 152.4, 148.8, 135.7, 128.5, 122.8, 120.9, 116.2, 115.63, 112.3, 105.4, 56.2.

HRMS (ESI+): 285.1057 m/z: Calculated for $C_{16}H_{17}N_2OS^+$ = 285.1062 $[M+H]^+$.

IR (ATR): 1606, 1462, 1431, 1368, 1222, 1166, 1058, 1023, 946, 832.

Decomposition point: decolorisation at 234.5 °C, melted at 242.3-243.3 °C, generated a dark vapour at 243.4 °C.

Characterisation data is in agreement with that reported by Qin *et al.*[5]

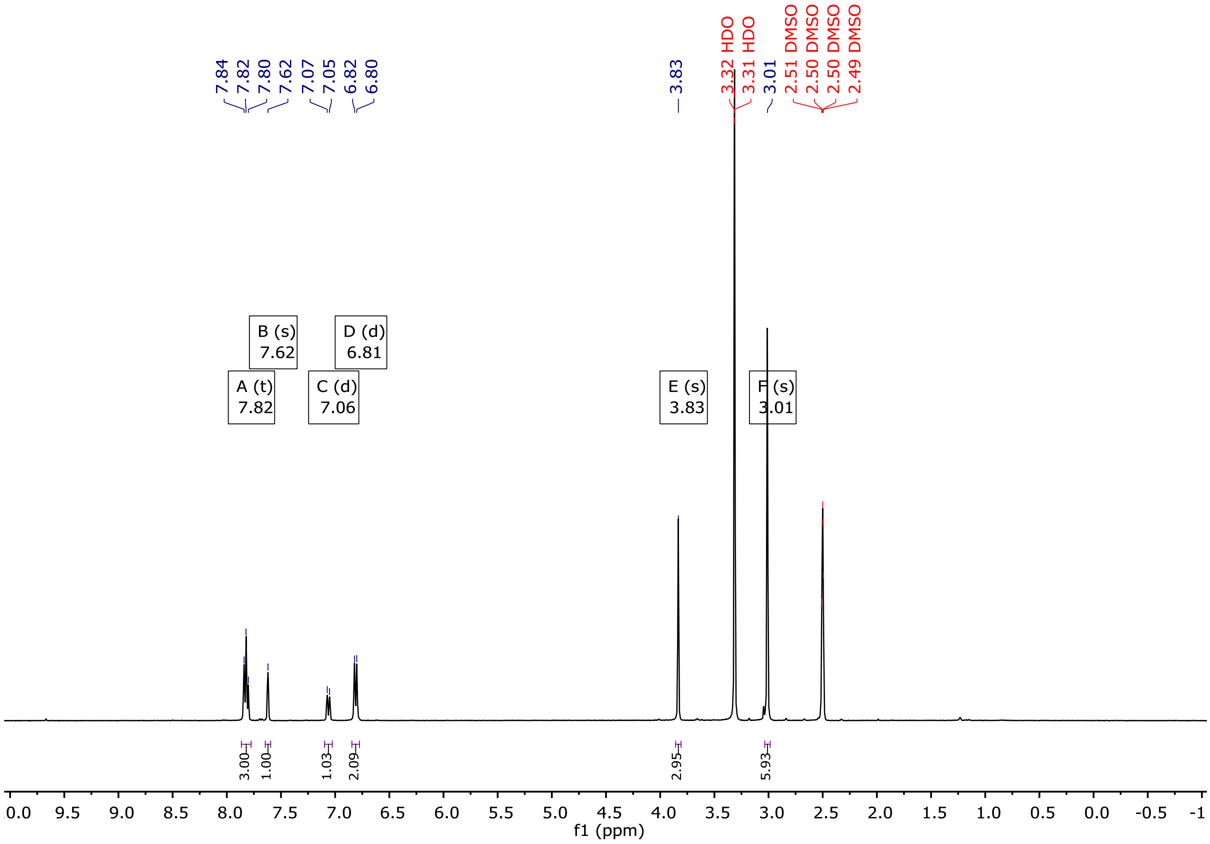

Figure S14. ^{1}H NMR (400 MHz, DMSO-d^{6}) spectra of **S05**.

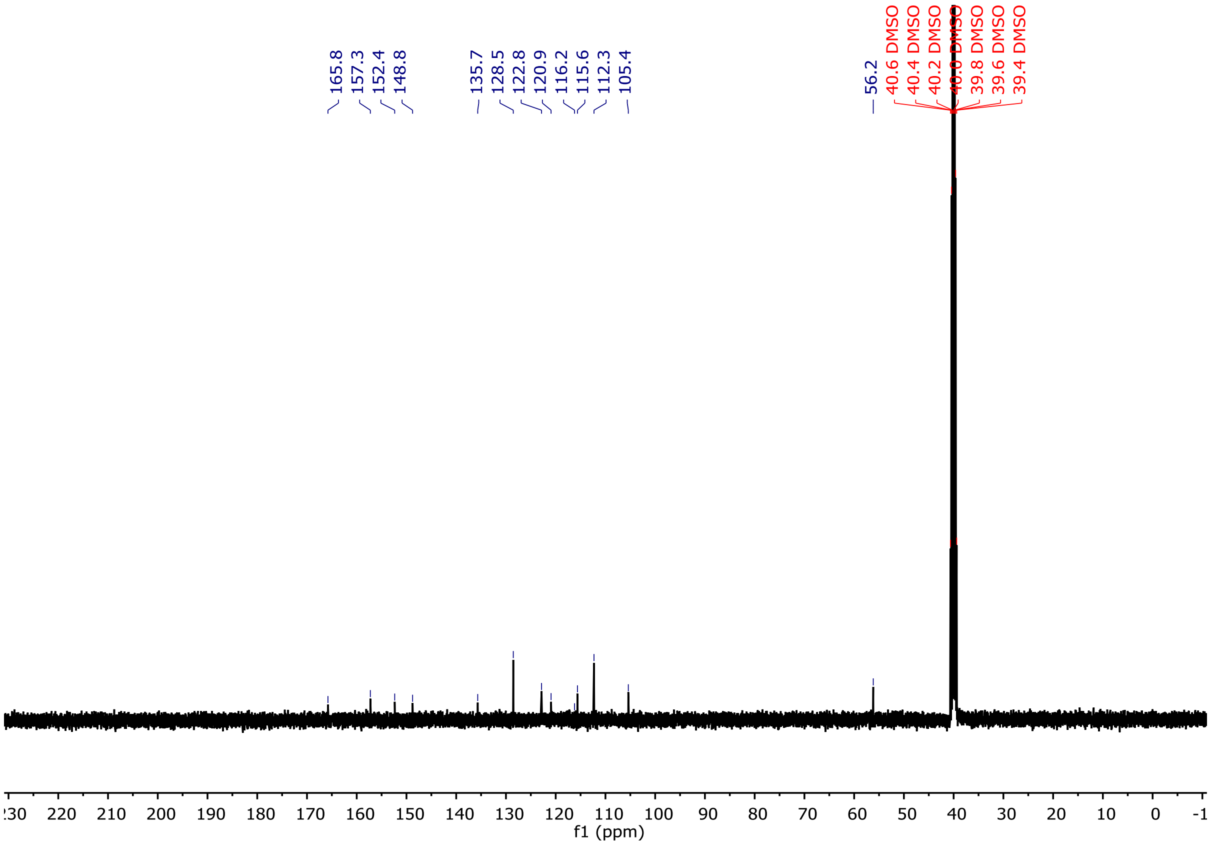

Figure S15. ^{13}C NMR (101 MHz, DMSO-d^{6}) spectra of **S05**.

2-(4-(dimethylamino)phenyl)benzo[*d*]thiazol-6-ol (S06)

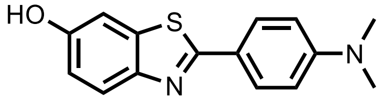

S06 was synthesised following a previously reported procedure.[4] To a solution of **S05** (21.0 g, 73.8 mmol) in anhydrous dichloromethane (800 mL) was added boron tribromide (242 mL, 1 M in dichloromethane) dropwise at 0 °C. The reaction mixture was warmed to room temperature and stirred for 17 h, then quenched by the addition of water (200 mL) then saturated sodium bicarbonate solution (200 mL). The precipitate was collected by vacuum filtration and washed with water (200 mL) then dichloromethane (200 mL) and dried *in vacuo* to afford **S06** as an orange solid (19.7 g, 72.9 mmol, 99%).

Aspect: orange solid. ***Yield***: 19.7 g (99%).

^{1}H NMR (400 MHz, DMSO-d^{6}), δ(ppm): 7.80 (d, *J* = 8.9 Hz, 2H), 7.72 (d, *J* = 8.7 Hz, 1H), 7.33 (d, *J* = 2.4 Hz, 1H), 6.92 (dd, *J* = 8.8, 2.4 Hz, 1H), 6.81 (d, *J* = 9.0 Hz, 2H), 3.01 (s, 6H).

^{13}C NMR (101 MHz, DMSO), δ(ppm): 164.4, 155.2, 151.5, 146.2, 134.8, 128.1, 122.1, 115.8, 112.6, 106.9.

HRMS (ESI+): 271.0905 m/z: Calculated for $C_{15}H_{15}N_2OS^+$ = 271.0900 $[M+H]^+$.

IR (ATR): 2956, 2920, 2851, 1606, 1475, 1427, 1371, 1339, 1278, 1195, 810.

Decomposition point: decolorisation at 230 °C, turned black at 240 °C, melted at 241.9-243.1 °C.

Characterisation data is in agreement with that reported by Qin *et al.*[5]

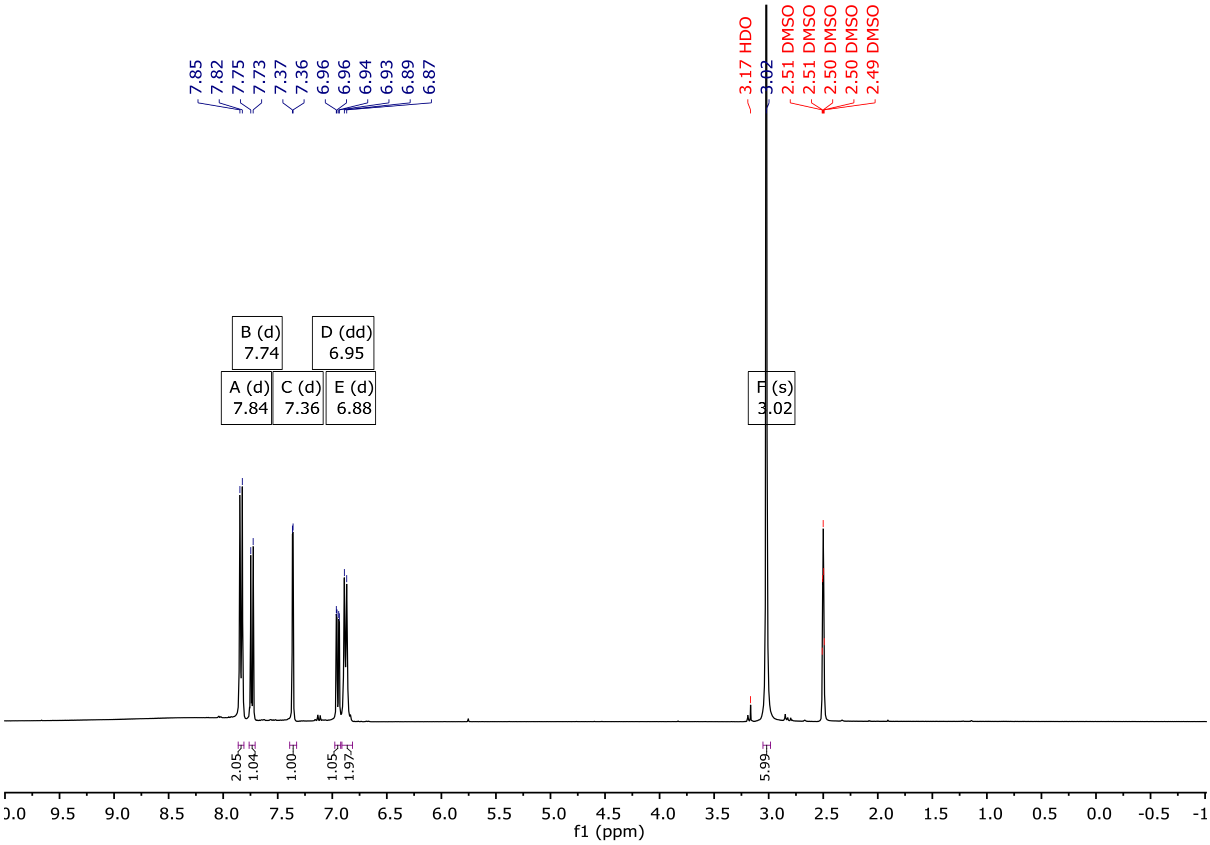

Figure S16. ^{1}H NMR (400 MHz, DMSO-d^{6}) spectra of **S06**.

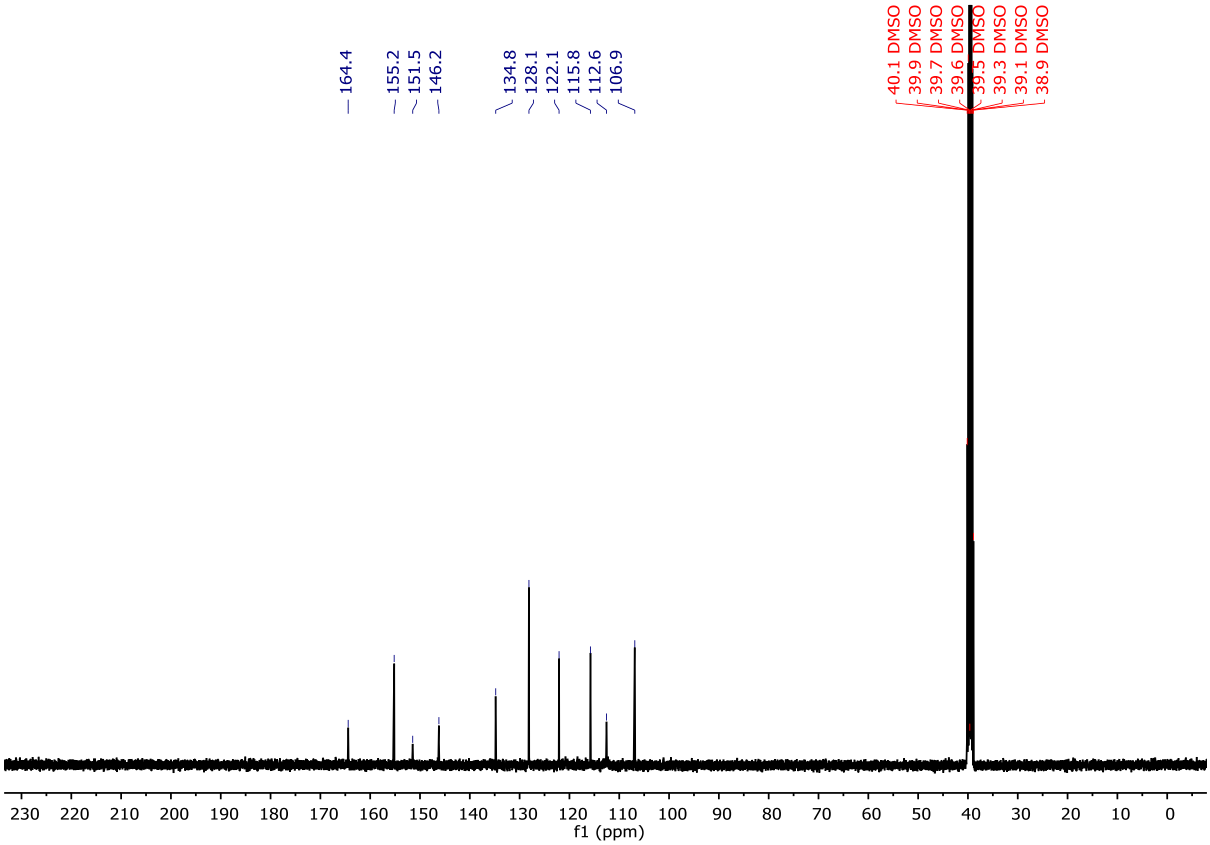

Figure S17. ^{13}C NMR (101 MHz, DMSO-d^{6}) spectra of **S06**.

2-(2-(2-((2-(4-(dimethylamino)phenyl)benzo[*d*]thiazol-6-yl)oxy)ethoxy)ethoxy)ethan-1-ol (BTA)

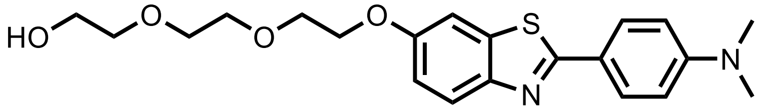

To a suspension of **S06** (503 mg, 1.86 mmol) and triphenylphosphine (1.21 g, 4.61 mmol) in anhydrous THF (100 mL) was added triethylene glycol (618 µL, 4.63 mmol) then DIAD (911 µL, 4.63 mmol). The reaction mixture was stirred at room temperature for 23 h. The solvent was evaporated under reduced pressure and the residue purified by flash chromatography (EtOAc:MeOH, 10:0 to 9:1) to afford BTA as a white solid (495 mg, 1.23 mmol, 67%).

Aspect: white solid. ***Yield***: 495 mg (67%).

^{1}H NMR (400 MHz, CDCl$_3$) δ 7.86 (d, *J* = 8.6 Hz, 2H), 7.83 (d, *J* = 8.9 Hz, 1H), 7.29 (d, *J* = 2.5 Hz, 1H), 7.03 (dd, *J* = 8.9, 2.5 Hz, 1H), 6.68 (d, *J* = 8.6 Hz, 2H), 4.13 (t, *J* = 4.8 Hz, 2H), 3.83 (t, *J* = 4.7 Hz, 2H), 3.73 – 3.68 (m, 4H), 3.68 – 3.64 (m, 2H), 3.58 (t, *J* = 4.6 Hz, 2H), 2.98 (s, 6H).

^{13}C NMR (101 MHz, CDCl$_3$) 166.8, 156.3, 152.1, 149.3, 135.9, 128.7, 122.9, 121.7, 115.6, 111.9, 105.6, 77.5, 77.2, 76.8, 72.6, 71.0, 70.6, 69.9, 68.2, 61.9, 40.3.

HRMS (ESI+): m/z: Calculated for $C_{21}H_{27}N_2O_4S^+$ = 403.1686 $[M+H]^+$.

IR (ATR, cm^{-1}): 2922, 2884, 1606, 1559, 1492, 1449, 1365, 1349, 1285, 1261,1223, 1188, 1138, 1126, 1101, 1068, 1043, 955, 942, 819.

MP: 188.3-189.3 °C.

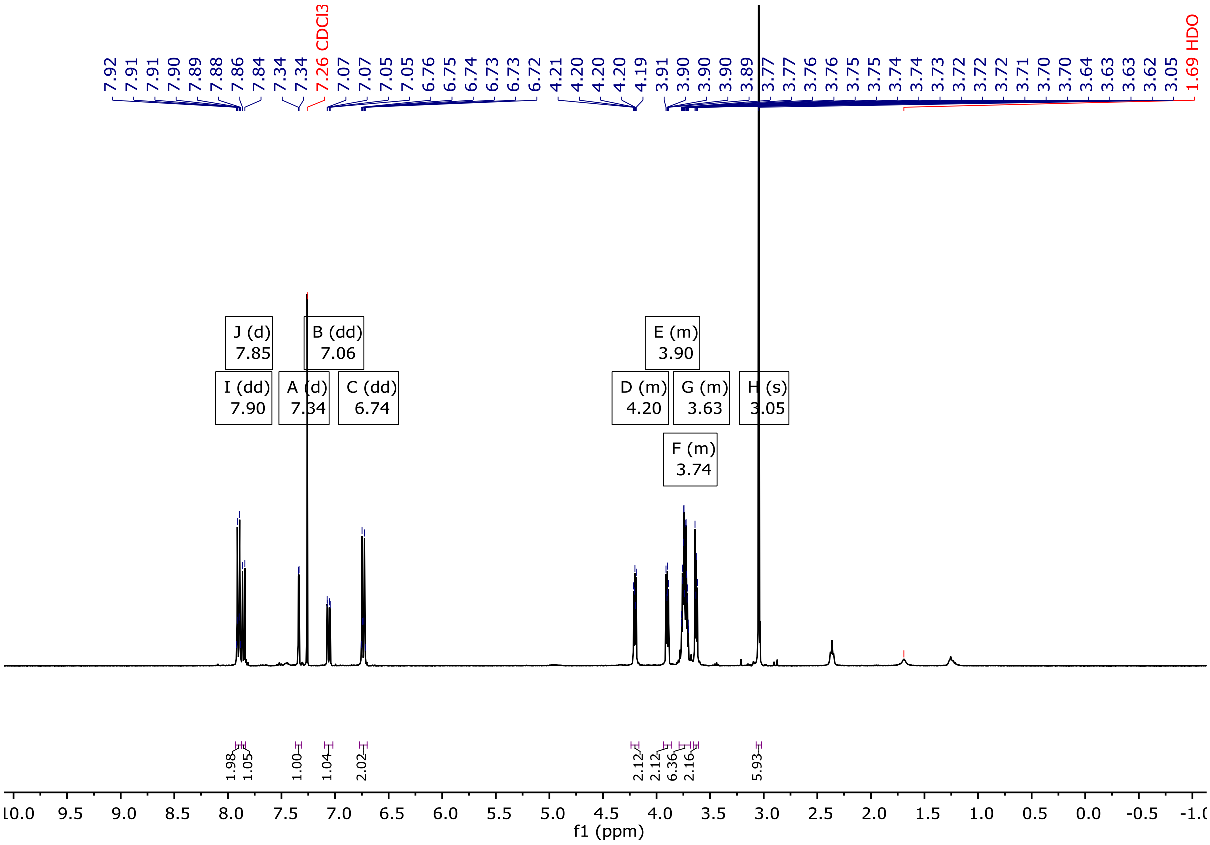

Figure S18. ^{1}H NMR (400 MHz, $CDCl_3$) spectra of BTA.

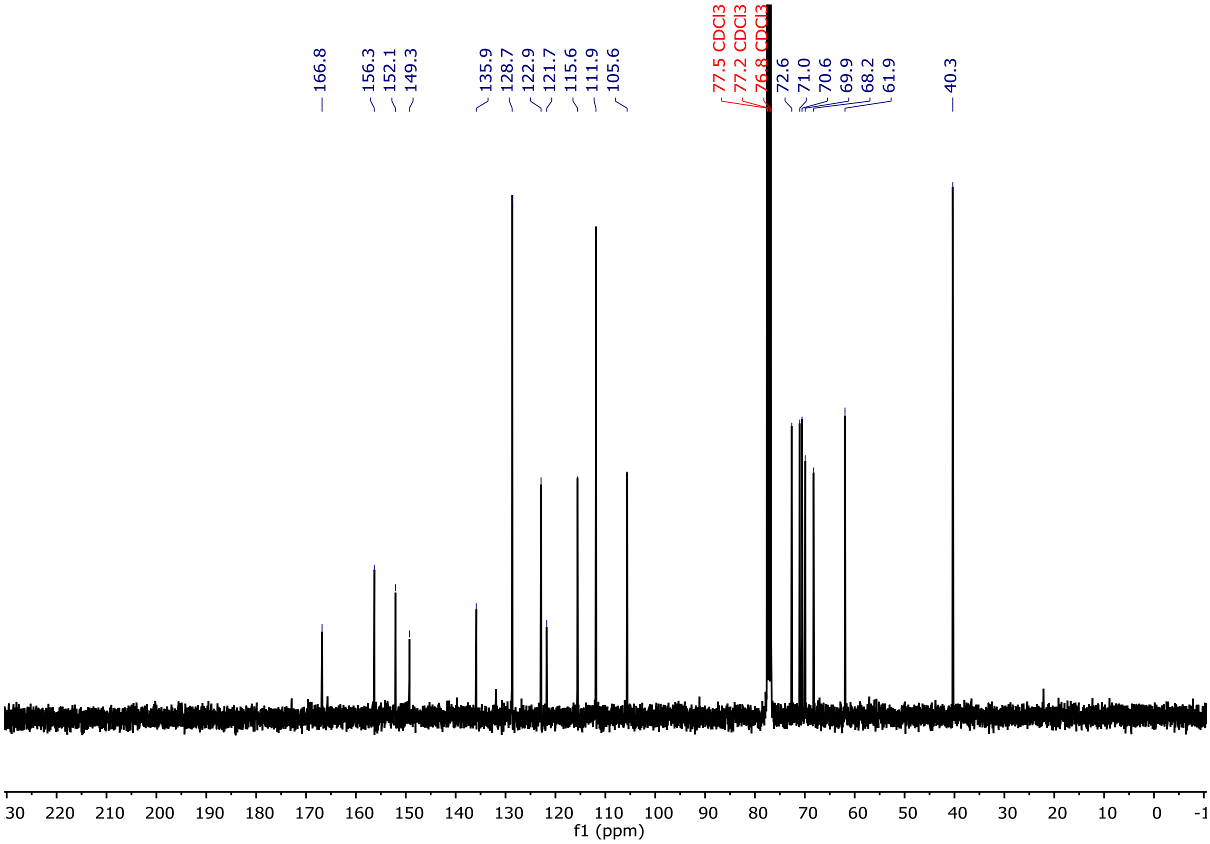

Figure S19. ^{13}C NMR (101 MHz, $CDCl_3$) spectra of BTA.

1-(4-nitrophenyl)-4-(pyridin-2-yl)piperazine (S07)

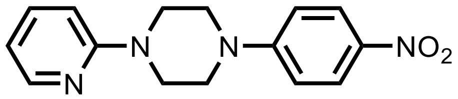

S07 was synthesised according to a previously reported procedure.[5] To 1-chloro-4-nitrobenzene (500 mg, 3.17 mmol, 1.0 equiv.), potassium *tert*butoxide (711 mg, 6.34 mmol, 2 equiv.), *tris*(dibenzylideneacetone)dipalladium (72 mg, 0.079 mmol, 0.02 equiv.), and RuPhos (74 mg, 0.05 equiv., 0.158 mmol) was added 1-(pyridin-2-yl)piperazine (1.03 g, 6.34 mmol, 2.0 equiv.) then 1,4-dioxane (10 mL) under an inert atmosphere. The reaction mixture was heated to 100 °C and stirred vigorously for 30 min. Upon completion by LCMS, the reaction mixture was cooled to rt and filtered through celite then the solvent removed *in vacuo*. The crude product was then purified using silica column chromatography (CH_2Cl_2 to CH_2Cl_2:MeOH 95:5) to afford **S07** as a brown solid (556 mg, 1.95 mmol, 62%).

Aspect: brown solid. ***Yield***: 556 mg (62%).

^{1}H NMR (400 MHz, $CDCl_3$) δ 8.22 (ddd, *J* = 5.0, 2.0, 1.0 Hz, 1H), 8.19 – 8.10 (m, 2H), 7.54 (ddd, *J* = 8.9, 7.2, 2.0 Hz, 1H), 6.87 – 6.82 (m, 2H), 6.71 – 6.67 (m, 2H), 3.80 – 3.73 (m, 4H), 3.63 – 3.56 (m, 4H).

^{13}C NMR (101 MHz, $CDCl_3$) δ 158.7, 154.7, 147.9, 138.7, 138.0, 126.2, 114.0, 112.6, 107.3, 46.7, 44.7.

HRMS (ESI+): 285.1356 m/z: Calculated for $C_{15}H_{17}N_4O_2^+$ = 285.1352 $[M+H]^+$.

IR (ATR, cm^{-1}): 2850, 1591, 1478, 1437, 1388, 1317, 1234, 1158, 1116, 1090, 1037, 979, 951, 906, 825.

Decomposition point: decolorisation at 131 °C, melted at 172-184 °C.

Characterisation data is in agreement with that reported by Ferrie *et al.*[6]

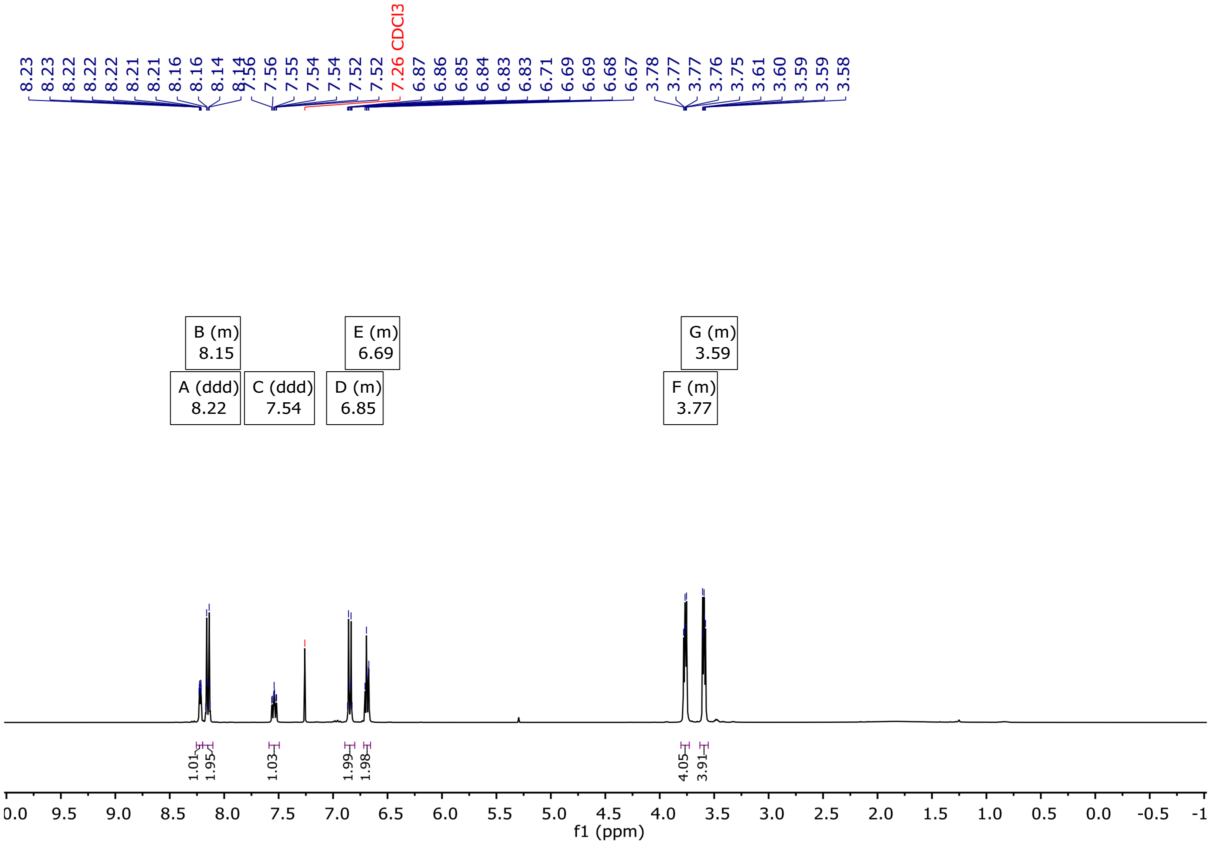

Figure S20. ^{1}H NMR (400 MHz, $CDCl_3$) spectra of **S07**.

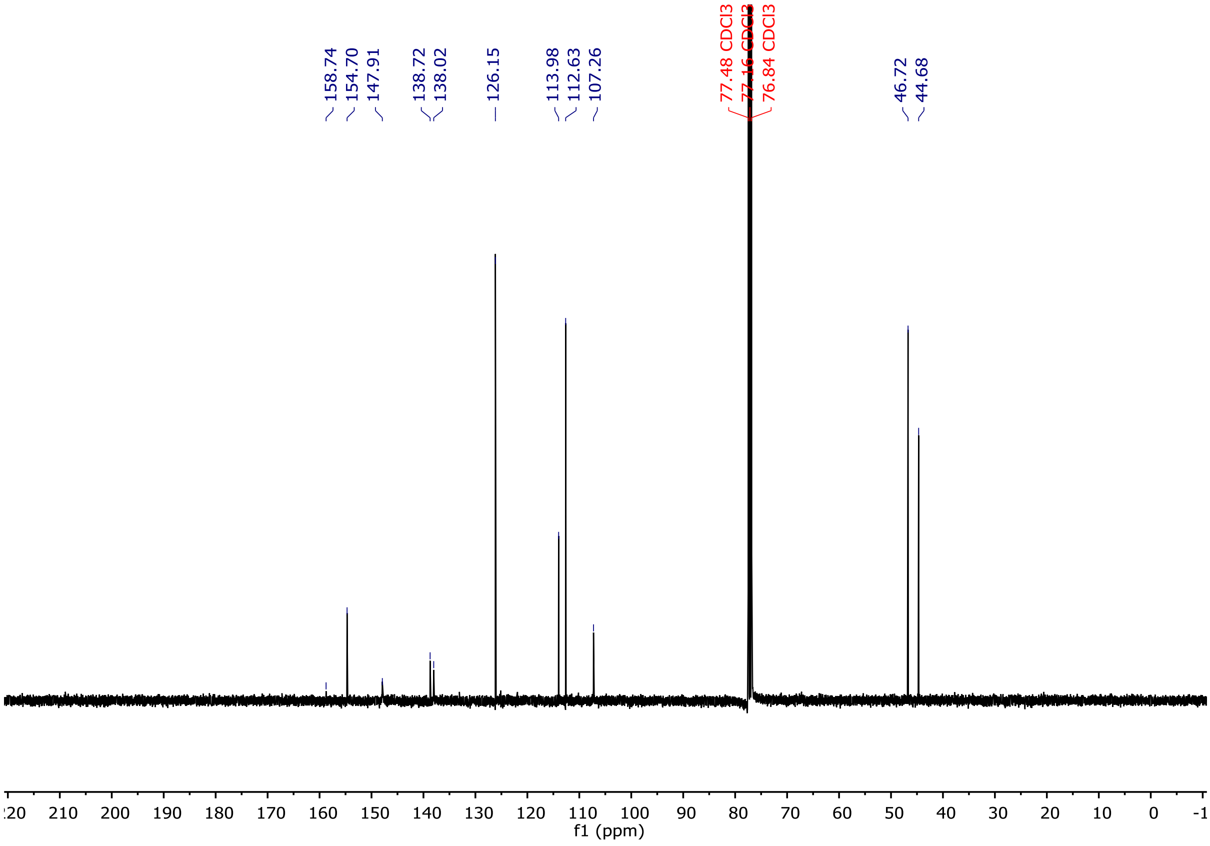

Figure S21. ^{13}C NMR (101 MHz, $CDCl_3$) spectra of **S06**.

(E)-2-(3-(4-(dimethylamino)phenyl)allylidene)malononitrile (AAR)

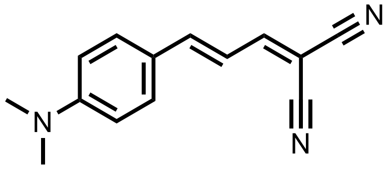

AAR was synthesised according to a previously reported procedure.[6] To a solution of 4-dimethylaminocinnamaldehyde (350 mg, 2.00 mmol, 1.00 eq.) and malonitrile (132 mg, 2.00 mmol, 1.00 eq.) in ethanol (20 mL, 0.1 M) was added piperidine (207 µL, 2.1 mmol, 1.05 eq.). The reaction mixture was stirred for 10 min at room temperature resulting in precipitation of red crystals. The crystals were filtered, washed with cold hexane, and then dried to afford AAR as a red crystalline solid (359 mg, 1.61 mmol, 81%).

Aspect: red crystals. ***Yield***: 359 mg (81%).

^{1}H NMR (500 MHz, CDCl$_3$) δ 7.52 – 7.45 (m, 3H), 7.17 (d, J = 14.8 Hz, 1H), 7.01 (dd, J = 14.8, 11.7 Hz, 1H), 6.68 (d, J = 9.1 Hz, 2H), 3.10 (s, 6H).

^{13}C NMR (126 MHz, CDCl$_3$) δ 160.6, 153.2, 151.7, 131.7, 122.0, 117.4, 115.1, 113.2, 112.1, 77.4, 77.2, 76.9, 76.3, 40.2.

HRMS (ESI+): 224.1191 m/z: Calculated for $C_{14}H_{14}N_3^+$ = 224.1182 $[M+H]^+$.

IR (ATR, cm^{-1}): 2918, 2863, 2812, 2216, 1584, 1540, 1440, 1374, 1320, 1288, 1241, 1164, 981, 944, 865, 814.

MP: 145.4-145.9 °C.

Characterisation data is in agreement with that reported by Cui *et al.*[7]

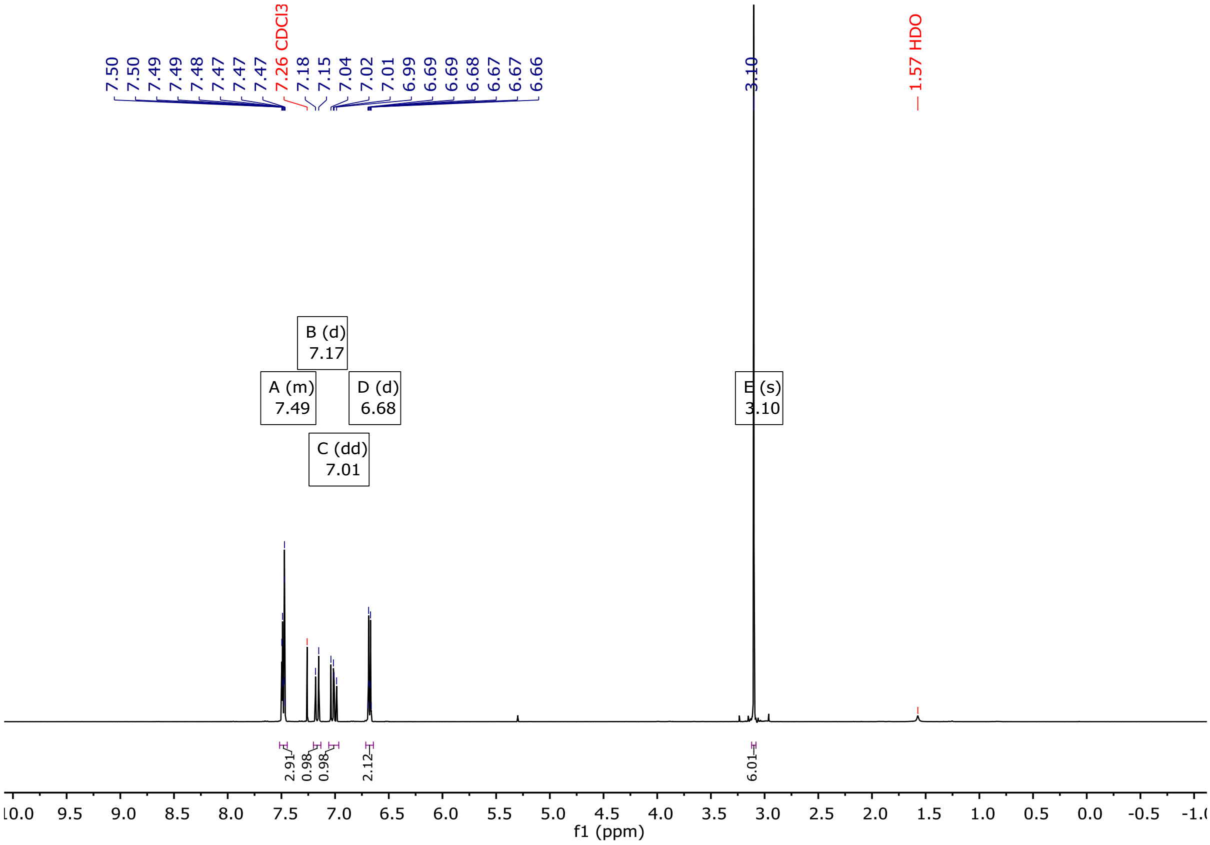

Figure S22. ^{1}H NMR (500 MHz, $CDCl_3$) spectra of AAR.

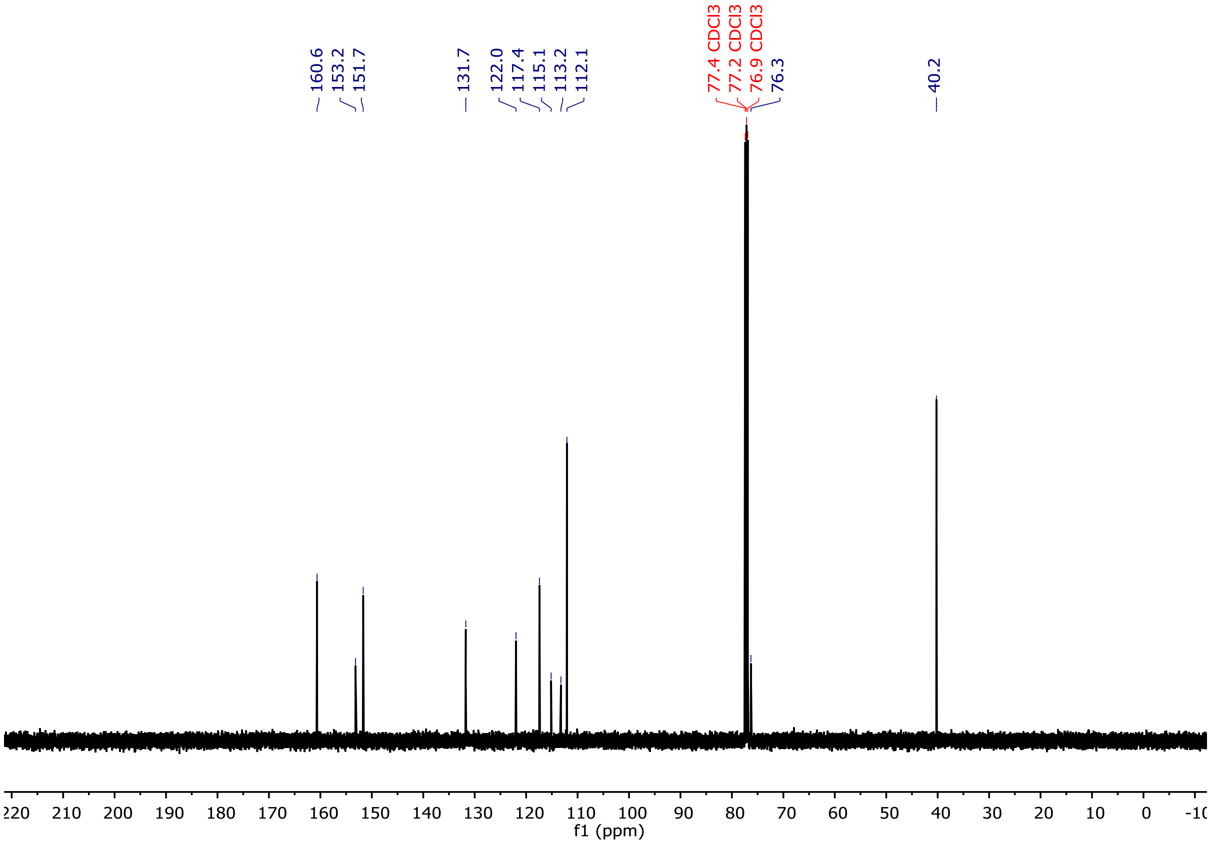

Figure S23. ^{13}C NMR (126 MHz, $CDCl_3$) spectra of AAR.

Preparation of α-Synuclein Fibrils

F, R, f65, f91, and f110 *In Vitro* α-Synuclein Fibrils

Human wild-type monomeric αSyn was expressed in *E. coli* strain BL21DE3 CodonPlus cells (Agilent Technologies), transformed with the expression vector pET3a (Novagen) bearing the *SNCA* gene, and purified from the bacterial lysate by ammonium sulfate precipitation, followed by PEI nucleic acid precipitation and passage through a DEAE sepharose column.[8] Fractions containing αSyn were heated to 80°C for 10 minutes and spun at 10,000g. The pure αSyn in the supernatant was filtered through sterile 0.22 µm filters. The concentration of αSyn was determined spectrophotometrically using an extinction coefficient of 5,960 M^{-1} cm^{-1} at 280 nm and it was stored at -80 °C in 50 mM Tris-HCl, pH 7.5, 150 mM KCl until use.

Monomeric αSyn concentration was adjusted to 350 µM in 50 mM Tris-HCl, pH 7.5, 150 mM KCl and the protein was either incubated as is or after buffer exchange to 5 mM Tris·HCl, pH 7.5; 20 mM KPO_4, pH 9.1 or 20 mM MES, pH 6.5 at 37 °C under continuous shaking (600 rpm) in an Eppendorf Thermomixer to generate the fibrillar polymorphs Fibrils, Ribbons, Fibrils 91 or Fibrils 65, respectively.[9,10] The polymorph Fibrils 110 was aggregated from monomeric αSyn lacking 30 C-terminal amino acid residues without buffer exchange.[11] Assembly under the different experimental conditions was monitored by thioflavin T binding using a Cary Eclipse Fluorescence Spectrophotometer (Varian Medical Systems Inc, λ_{ex} = 440 nm, λ_{em} = 480 nm).

PD, MSA, and DLB PMCA α-Synuclein Fibrils

Monomeric αSyn was prepared as above. Frozen brain tissues, obtained in compliance with the ethics committee guidelines of the University of Paris (Neuro-CEB Brain Bank), from female individuals with either PD (68 years old), DLB (71 years old) or MSA (64 years old) were homogenized (10%, weight:volume) in 150 mM KCl, 50 mM Tris·HCl, pH 7.5 in BSL-3. Tissue homogenates were diluted in PMCA buffer (150 mM KCl, 50 mM Tris·HCl, pH 7.5) containing monomeric αSyn (100 µM) to a final concentration of 2% (weight:volume). The samples were split in two tubes of PCR strips (BIOplastics, Landgraaf, The Netherlands). PMCA amplification was performed in duplicates for each patient using the Q700 generator and a 431MPX horn (Qsonica, Fisher Scientific, Illkirch, France). The power of the horn was set to 30% of maximal amplitude. The program of amplification consisted in 15 s of sonication and a 5-min pause at 30°C as previously described.[12] Every hour, 5 µL were withdrawn from each tube and diluted in 300 µL of 10 µM thioflavin T and the amplification was monitored by measuring fluorescence as for the *de novo* assembled polymorphs. At time 430 min, the reaction product was diluted (2% volume:volume) into a fresh solution of αSyn (100 µM) and two additional 200 min amplification cycles were performed. The resulting fibrils were diluted (5% volume:volume) into a fresh solution of αSyn (100 µM) and the fibrils were allowed to elongate at 30°C overnight.

Biophysical Characterisation of α-Synuclein Fibrils

Circular Dichroism Spectra

Circular dichroism (CD) spectra of αSyn fibrils (0.1 μM for *PMCA* fibrils, 1.0 μM for *de novo* fibrils) and Aβ(1-42) fibrils (1.0 μM) in 1xPBS (pH 7.4) were recorded with a Chirascan CD1 Spectrometer (Applied Photonics Ltd.) equipped with a Series 800 Temperature Controller (Alpha Omega Instruments). Far-ultraviolet measurements (190-250 nm) were recorded at 25 °C with a 1.0 cm optical pathlength, a time-per-point of 1.0 s, a 1.0 nm bandwidth, and a wavelength step of 0.1 nm. CD spectra were averaged over six scans. Data were baseline corrected by subtracting the complete buffer spectrum of 1xPBS (pH 7.4) averaged over six scans. Applied Photophysics Pro-Data Chirascan software was used to smooth the data using Savitsky-Golay smoothing and a window size of eight, and the data was converted to molar ellipticity.

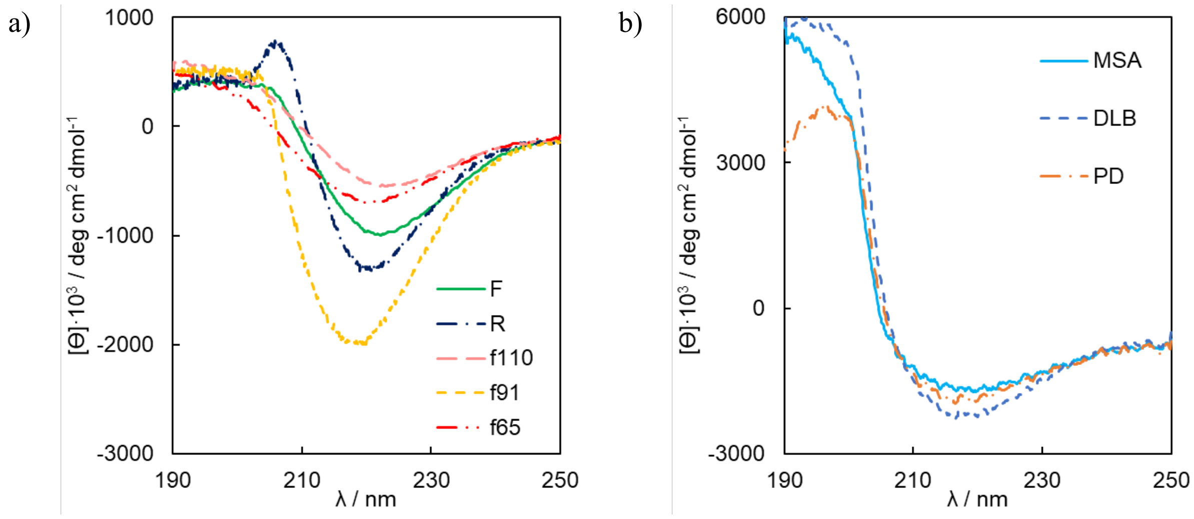

Figure S24. Circular dichroism (CD) spectra of a) *de novo* αSyn fibrils (1.0 μM), and b) *PMCA* αSyn fibrils (0.1 μM) recorded in aqueous 1xPBS buffer (pH 7.4, 25 °C).

Transmission Electron Microscopy

Fibrils were imaged by transmission electron microscopy after adsorption onto carbon-coated 200 mesh grids and negative staining with 1% uranyl acetate using a Jeol 1400 transmission electron microscope and the images were recorded with a Gatan Orius CCD camera (Gatan, Pleasanton).

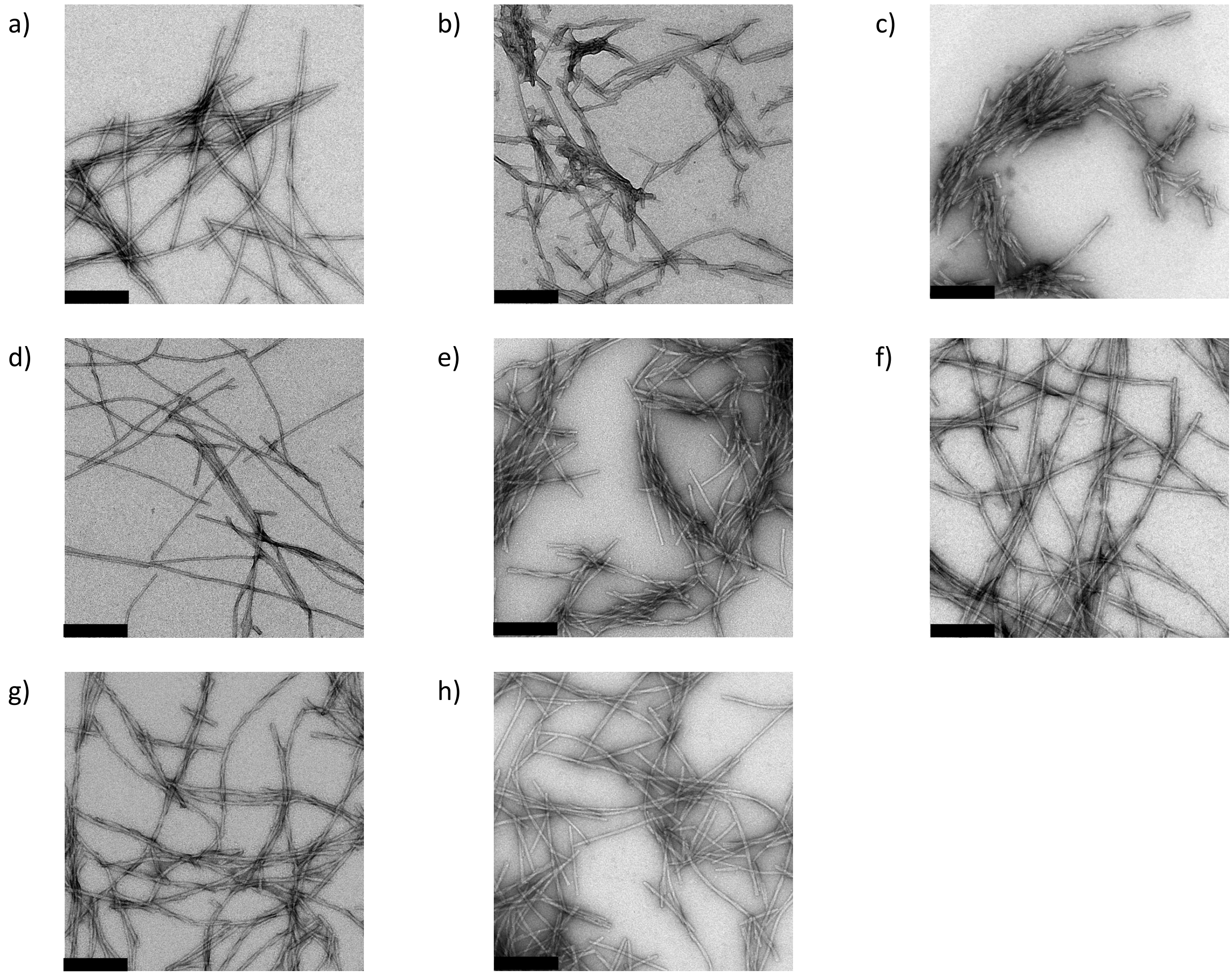

Figure S25. TEM images of the different αSyn fibril morphologies studied: a) F, b) R, c) f110, d) f91, e) f65, f) PD, g) MSA, and h) DLB. The scale bar displayed is 100 nm.

Limited Proteolysis

Limited proteolysis of the αSyn fibrils F, R, f65, f91, f110, PD, MSA, and DLB was performed by Professor Ronald Melki. Fibrils were treated at 37 °C by Proteinase K (3.8 µg/ml) (Roche). Aliquots were withdrawn at 1, 5, 15, 30 and 60 minutes following addition of the protease and transferred into Eppendorf tubes containing denaturing sample buffer (50 mM Tris·HCl, pH 6.8, 4% SDS, 2% β-mercaptoethanol, 12% glycerol and 0.01% bromophenol blue) maintained at 90°C to arrest immediately the cleavage reaction. After incubation for 5 min at 90°C, the samples were processed to monitor the time course of αSyn cleavage by polyacrylamide gel electrophoresis (PAGE) (15%) after staining with Coomassie blue.[12]

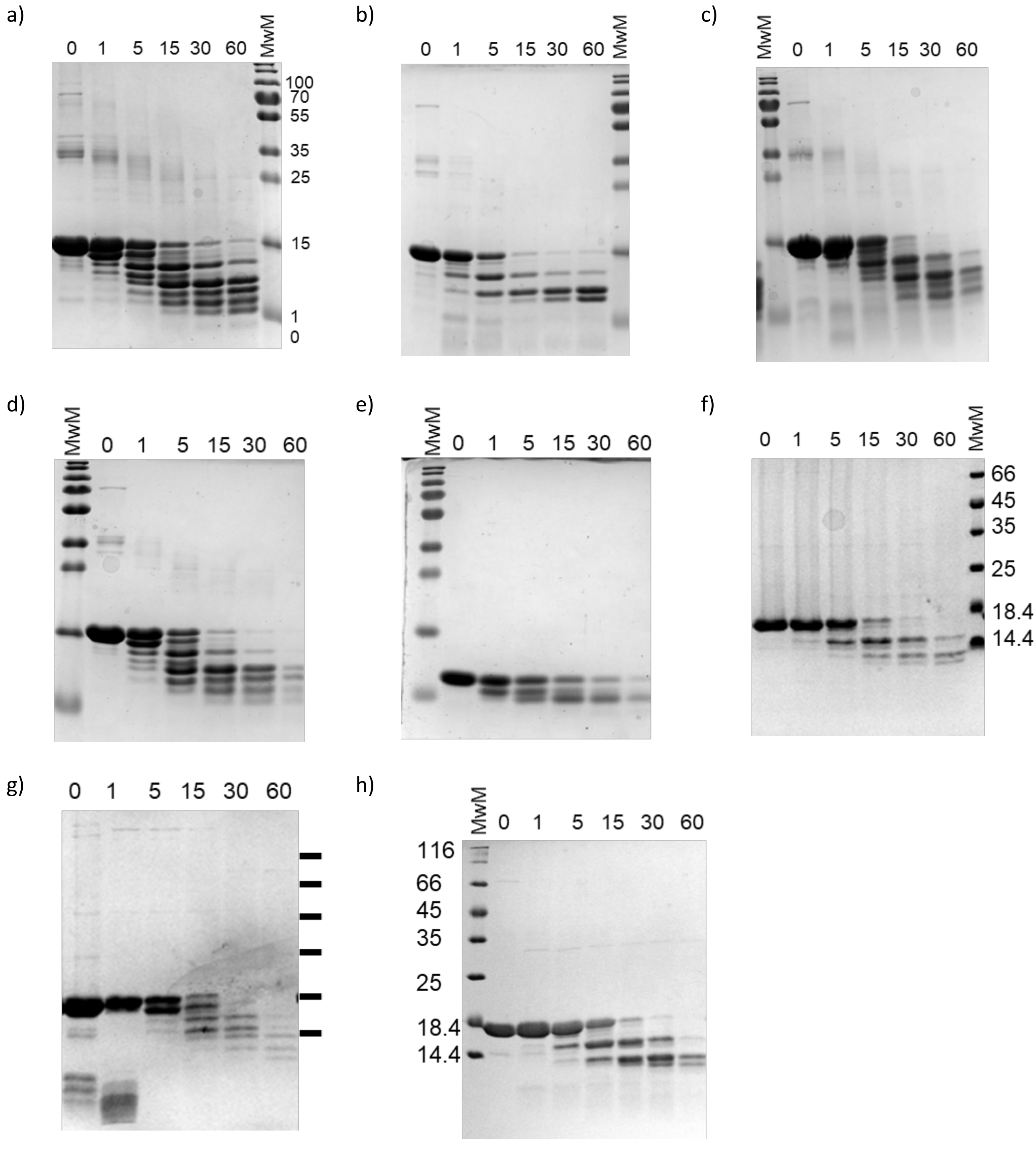

Figure S26. Proteinase K proteolytic profiles of the αSyn fibrillar polymorphs (a) F, (b) R, (c) f91, (d) f65, (e) f110, (f) PD, (g) DLB, and (h) MSA. Molecular weight markets are in kDa.

UV-Visible Characterisation

The UV-visible characterisation of ThT, OXI, ThR, BTA, and S5H has been previously reported.[1]

Stock solutions of ligand in DMSO (10 mM) were diluted into ethanol to obtain a 50 µM solution and placed into a quartz fluorescence cuvette (Hellma Analytics) with a 1 cm pathlength. UV-visible spectra were obtained with an Agilent Cary 60 UV-vis spectrophotometer controlled by Cary WinUV software using a scan rate of 600 nm/min, a data interval of 1.0 nm and an averaging time of 0.10 at 25°C.

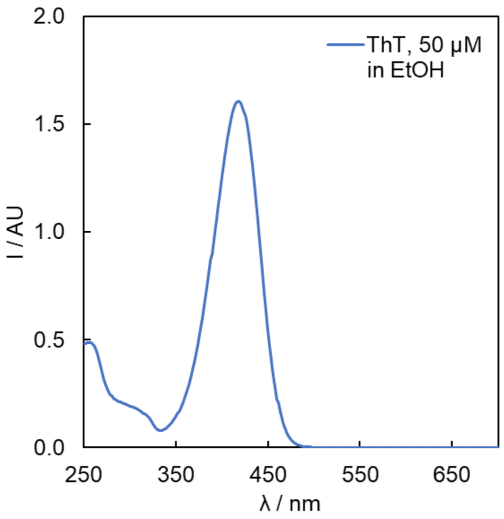

Figure S27. UV-vis spectrum of ThT (50 µM) in EtOH at 298 K, with λ_{max} = 418 nm.

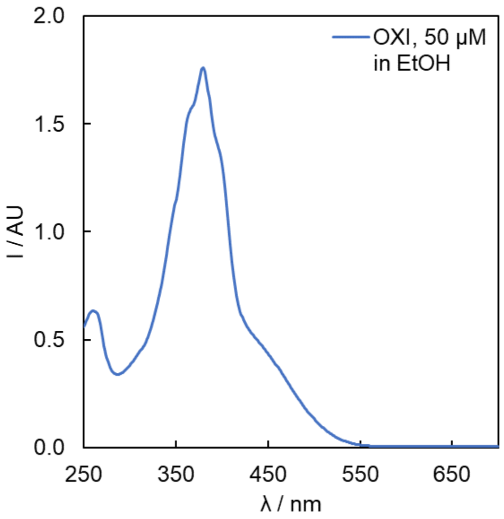

Figure S28. UV-vis spectrum of OXI (50 µM) in EtOH at 298 K, with λ_{max} = 380 nm.

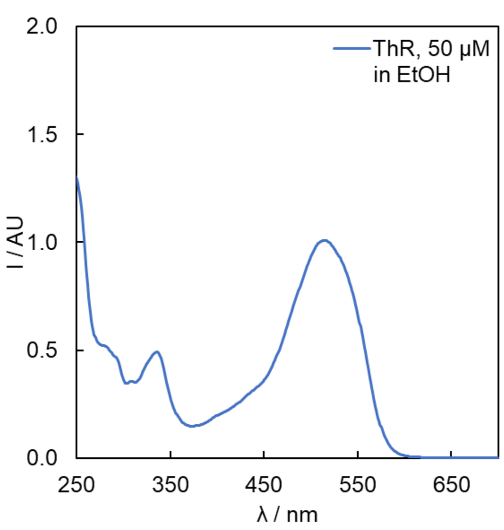

Figure S29. UV-vis spectrum of ThR (50 μM) in EtOH at 298 K, with λ_{max} = 515 nm.

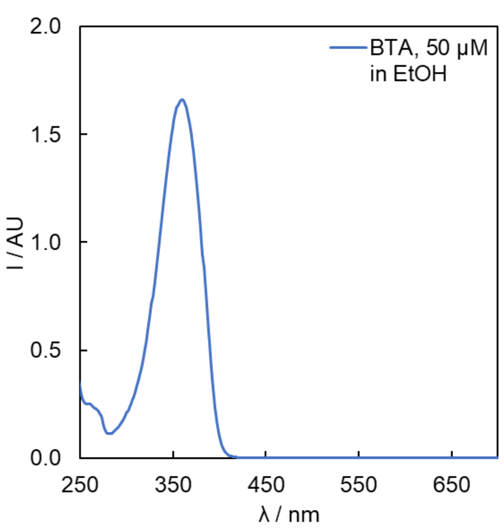

Figure S30. UV-vis spectrum of BTA (50 μM) in EtOH at 298 K, with λ_{max} = 359 nm.

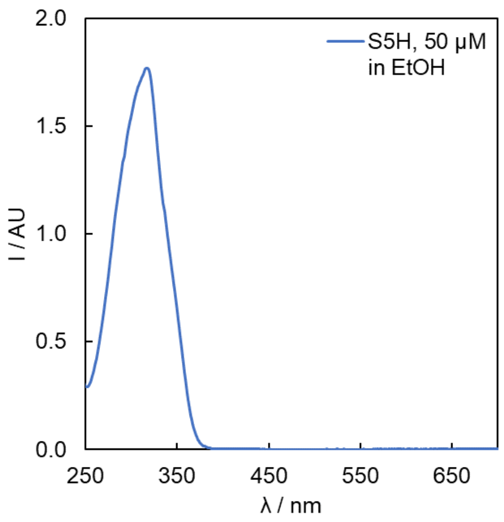

Figure S31. UV-vis spectrum of S5H (50 μM) in EtOH at 298 K, with λ_{max} = 318 nm.

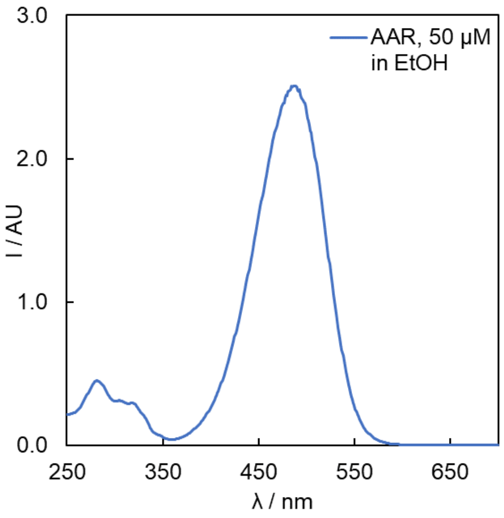

Figure S32. UV-vis spectrum of AAR (50 μM) in EtOH at 298 K, with λ_{max} = 487 nm.

Table S1. Absorbance maxima of ligands (50 μM) in EtOH at 298 K.

Ligand	λ_{max} / nm
ThT	418
OXI	380
ThR	515
BTA	359
S5H	318
AAR	487

Fluorescence Characterisation

The fluorescence characterisation of ThT, OXI, ThR, BTA, and S5H has been previously reported.[1]

Fluorescence spectral readings were performed on an Agilent Cary Eclipse Fluorescence Spectrophotometer using a scan rate of 600 nm/min, a data interval of 1.0 nm and an averaging time of 0.10 at 25°C. Fluorescence experiments used 20 nm excitation and emission slits and medium PMT voltage, except when specified otherwise. Fluorescence spectra of **BTA** were recorded with 10 nm excitation and emission slits, and low PMT voltage.

Spectral experiments detecting **ThT** used λ_{ex} = 440 nm, and measured emissions from λ_{em}= 470 – 600 nm. Spectral experiments detecting **BTA** used λ_{ex} = 360 nm, and measured λ_{em}= 380 – 600 nm. Spectral experiments detecting **AAR** used λ_{ex} = 522 nm, and measured λ_{em}= 562 – 700 nm.

Fluorescence anisotropy experiments were performed using 10 nm excitation and emission slits, a scan rate of 120 nm/min, a data interval of 1.0 nm, an averaging time of 0.5 s, and medium PMT voltage at 25 °C. A reference solution of **BTA** in DMSO (2 µM) was used to calculate G-factors. A G-Factor voltage of 425 V was used, and a polarisation/anisotropy voltage of 740 V was used. Anisotropy experiments detecting **BTA** used λ_{ex} = 360 nm, and measured λ_{em}= 433 – 453 nm.

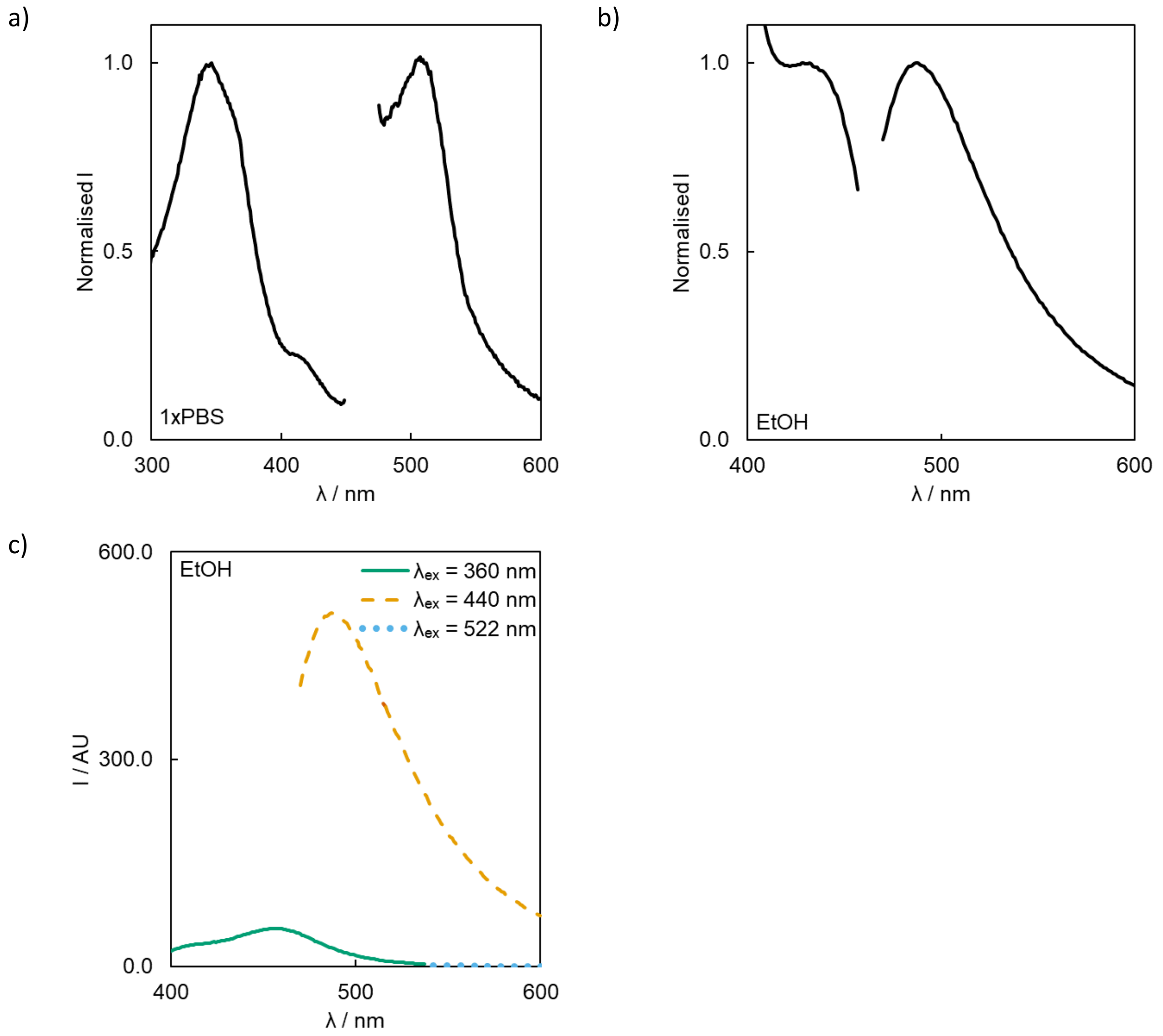

Figure S33. Fluorescence spectra of ThT in (a) 1xPBS (pH 7.4, 2.0 μM, λ_{ex} = 440 nm, λ_{em} = 485 nm, 298 K); (b) EtOH (50 μM, λ_{ex} = 440 nm, λ_{em} = 487 nm, 298 K); (c) EtOH (50 μM) upon excitation at the wavelengths of the other fluorescence ligands used at 298 K.

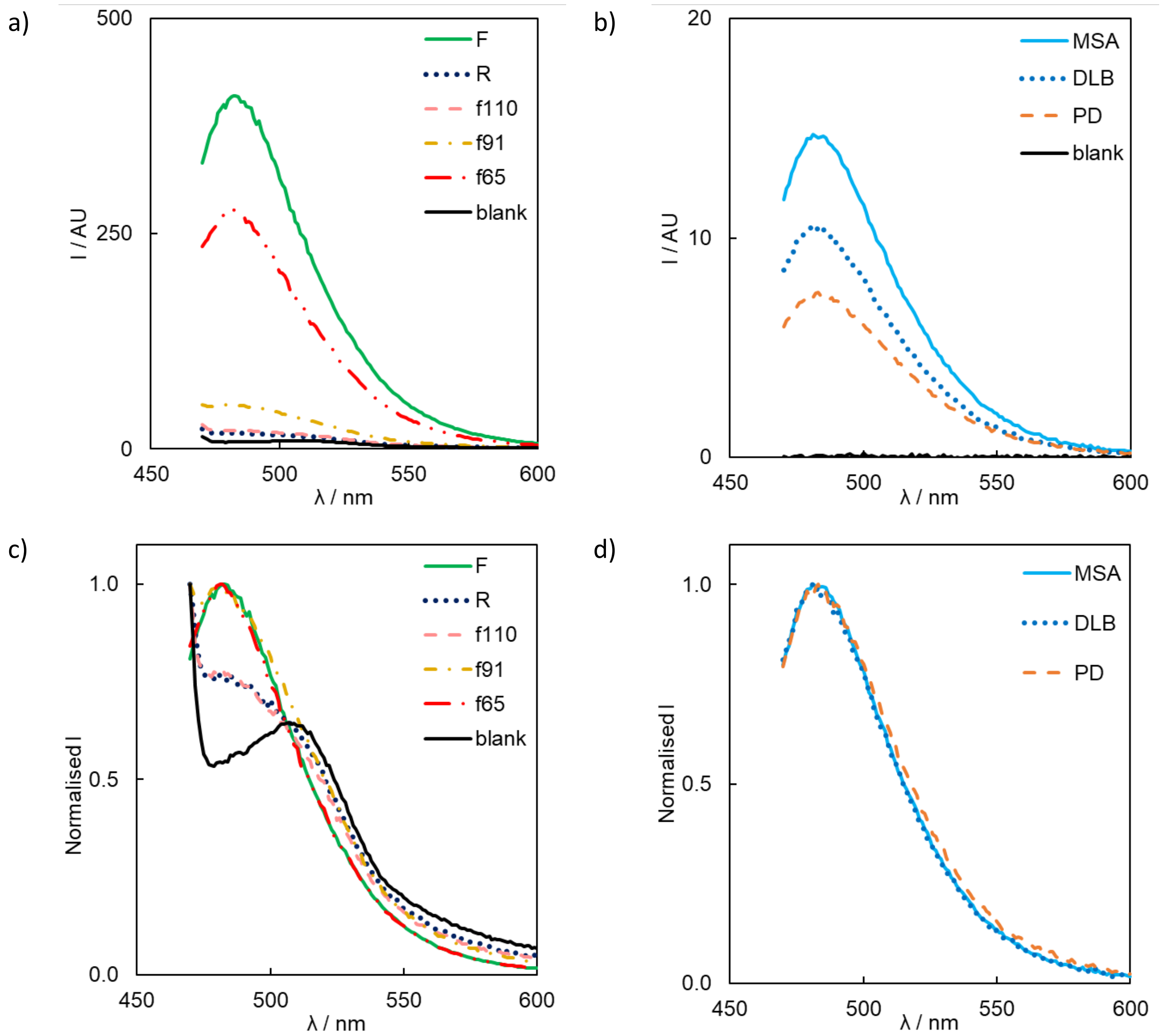

Figure S34. Additional fluorescence emission spectra (λ_{ex} = 440 nm, 298 K) of ThT (1.0 μM) binding to (a) F, R, f110, f91, and f65 αSyn fibrils (500 nM), and (b) MSA, DLB, and PD fibrils (500 nM) in 1xPBS (pH 7.4). The corresponding normalised spectra are shown in panels (c) and (d). Blank spectra were recorded of the free ligand in the absence of any αSyn fibrils.

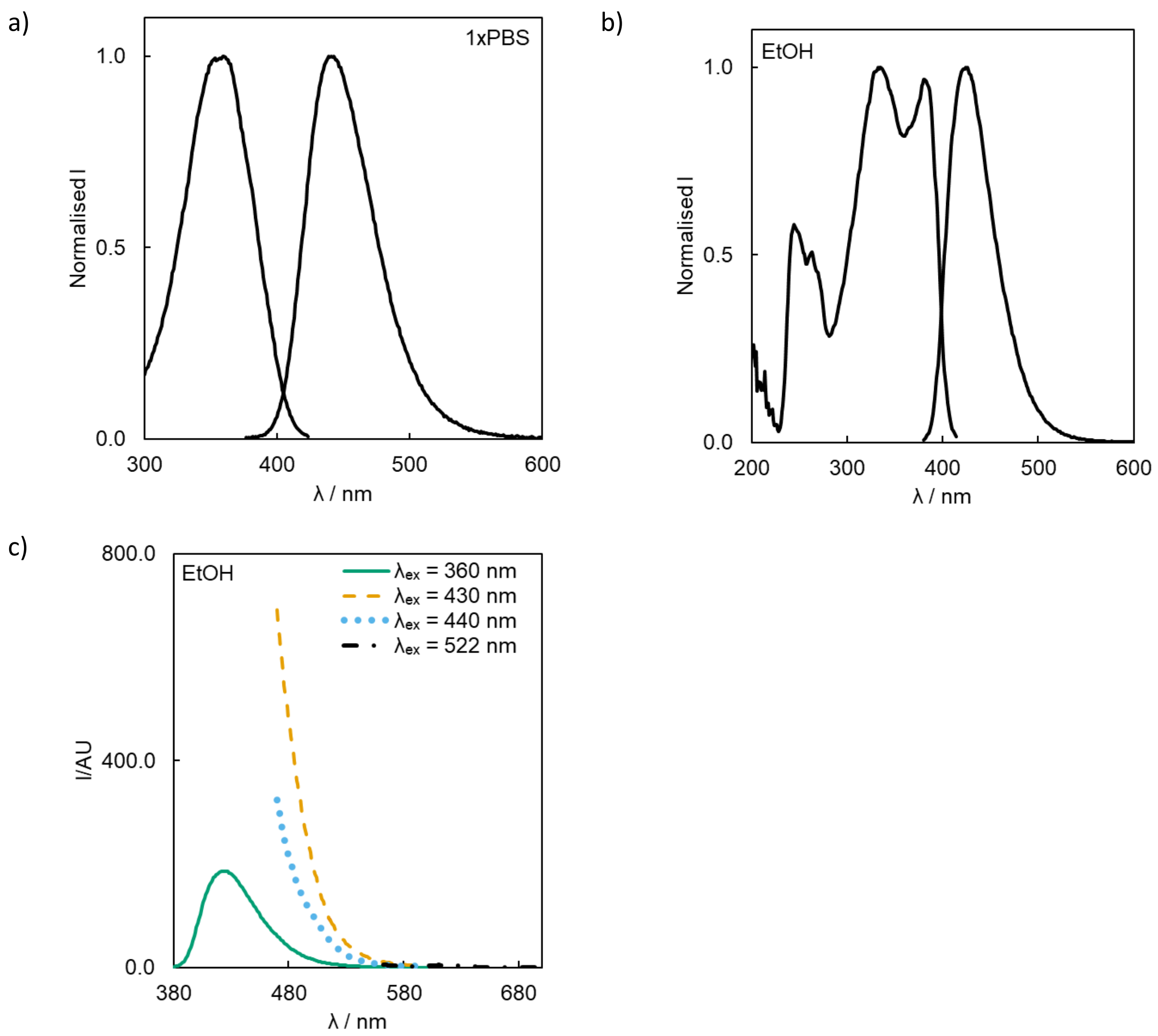

Figure S35. Fluorescence spectra of BTA in (a) 1xPBS (pH 7.4, 2.0 µM, λ_{ex} = 357 nm, λ_{em} = 443 nm, 298 K); (b) EtOH (50 µM, λ_{ex} = 360 nm, λ_{em} = 424 nm, 298 K); (c) EtOH (50 µM) upon excitation at the wavelengths of the other fluorescence ligands used at 298 K.

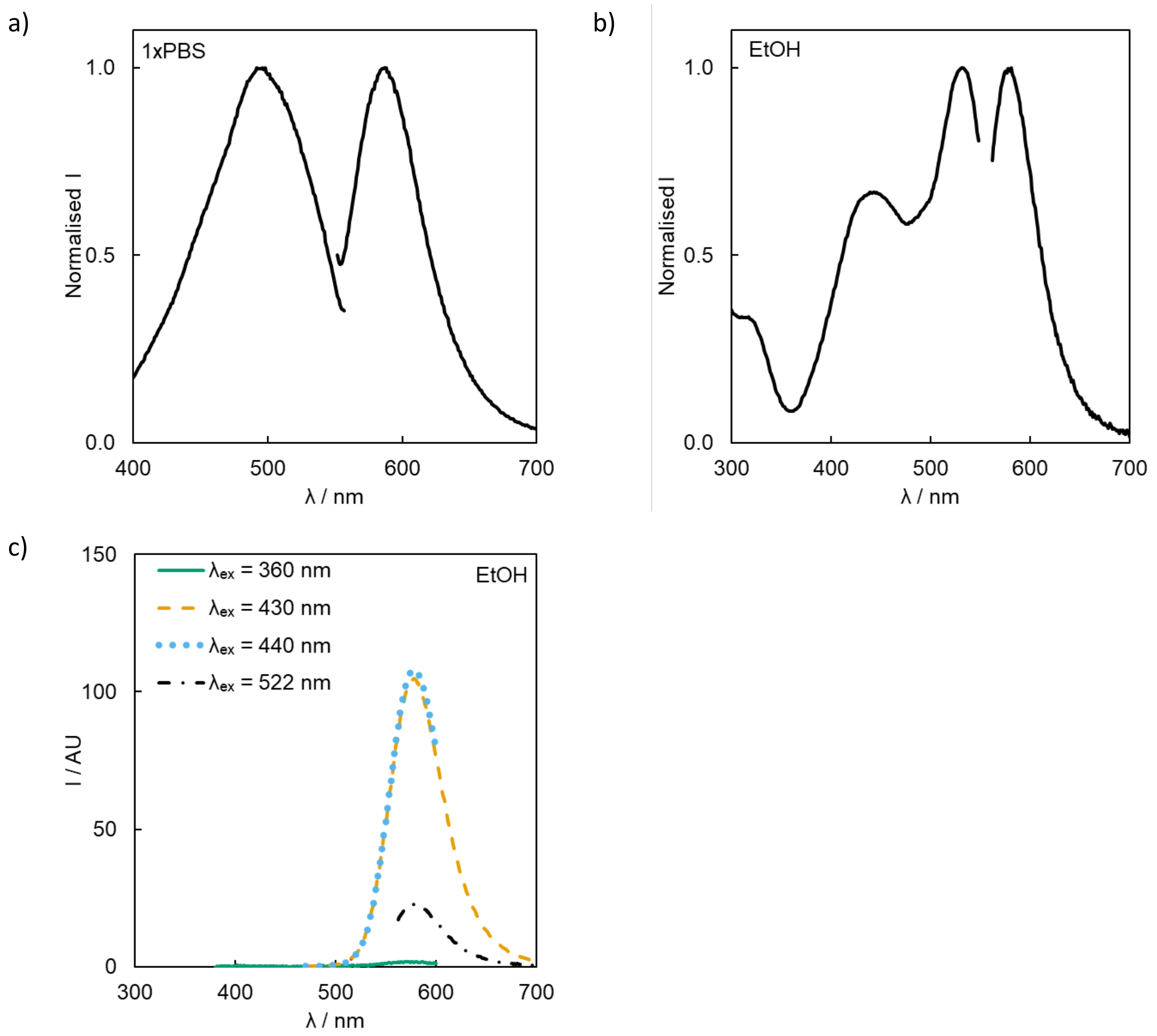

Figure S36. Fluorescence spectra of AAR in (a) 1xPBS (pH 7.4, 2.0 µM, λ_{ex} = 522 nm, λ_{em} = 587 nm, 298 K); (b) EtOH (50 µM, λ_{ex} = 522 nm, λ_{em} = 578 nm, 298 K); (c) EtOH (50 µM) upon excitation at the wavelengths of the other fluorescence ligands used at 298 K.

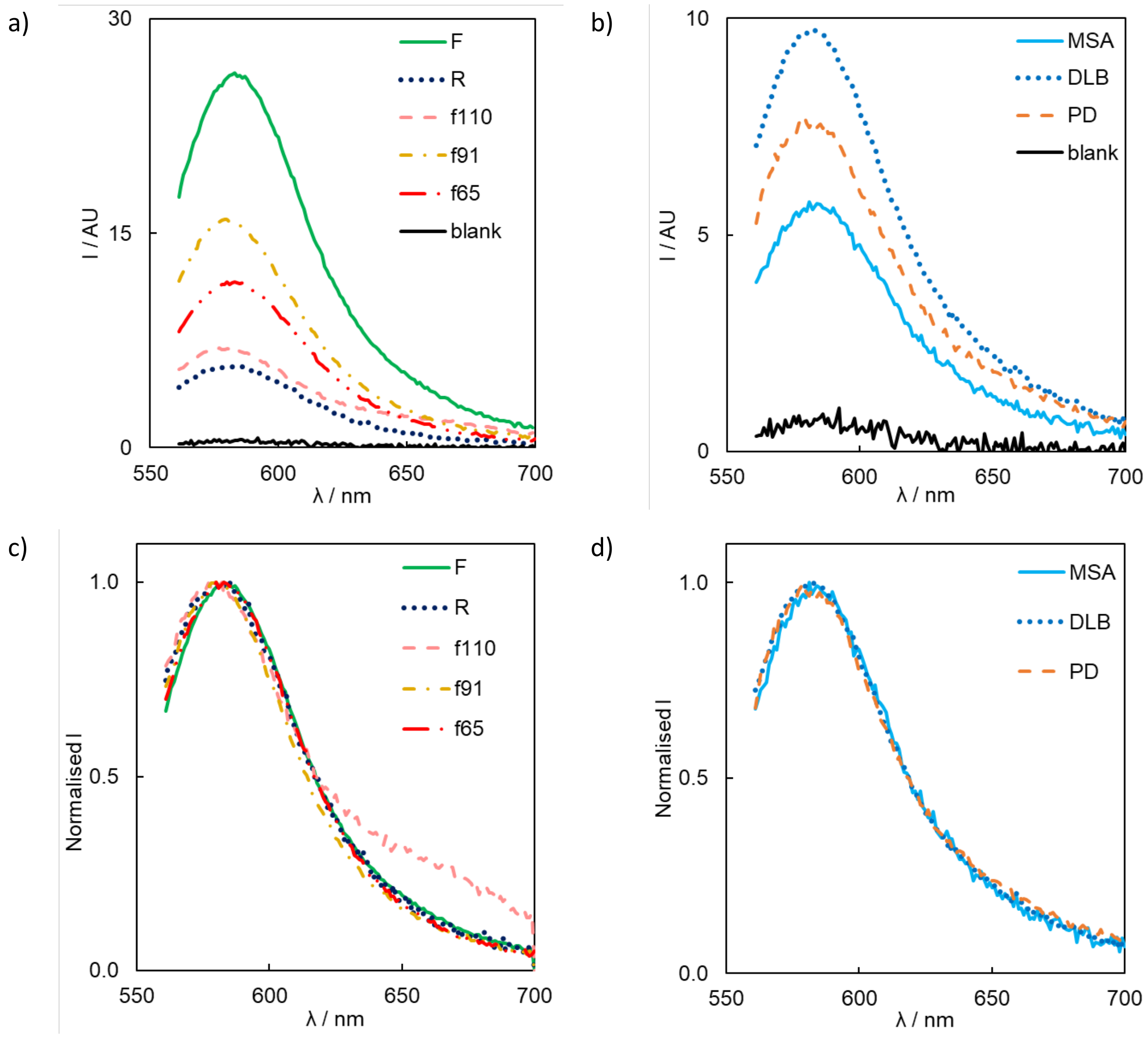

Figure S37. Fluorescence emission spectra (λ_{ex} = 522 nm, 298 K) of AAR (5.0 µM) binding to (a) F, R, f110, f91, and f65 αSyn fibrils (500 nM), and (b) MSA, DLB, and PD fibrils (250 nM) in 1xPBS (pH 7.4). The corresponding normalised spectra are shown in panels (c) and (d). Blank spectra were recorded of the free ligand in the absence of any αSyn fibrils.

Table S2. Emission and excitation maxima for ThT, BTA, and AAR in 1xPBS or EtOH at 298 K.

	$\lambda_{ex,\ 1xPBS}$ / nm	$\lambda_{em,\ 1xPBS}$ / nm	$\lambda_{ex,\ EtOH}$ / nm	$\lambda_{em,\ EtOH}$ / nm
ThT	346	506	430	487
BTA	353	441	334	425
AAR	500	587	532	575

Dilution Series

Dilution series of ligands were performed according to the general methods. Dilution series of ThT, OXI, S5H, BTA, and ThR have been previously reported.[1]

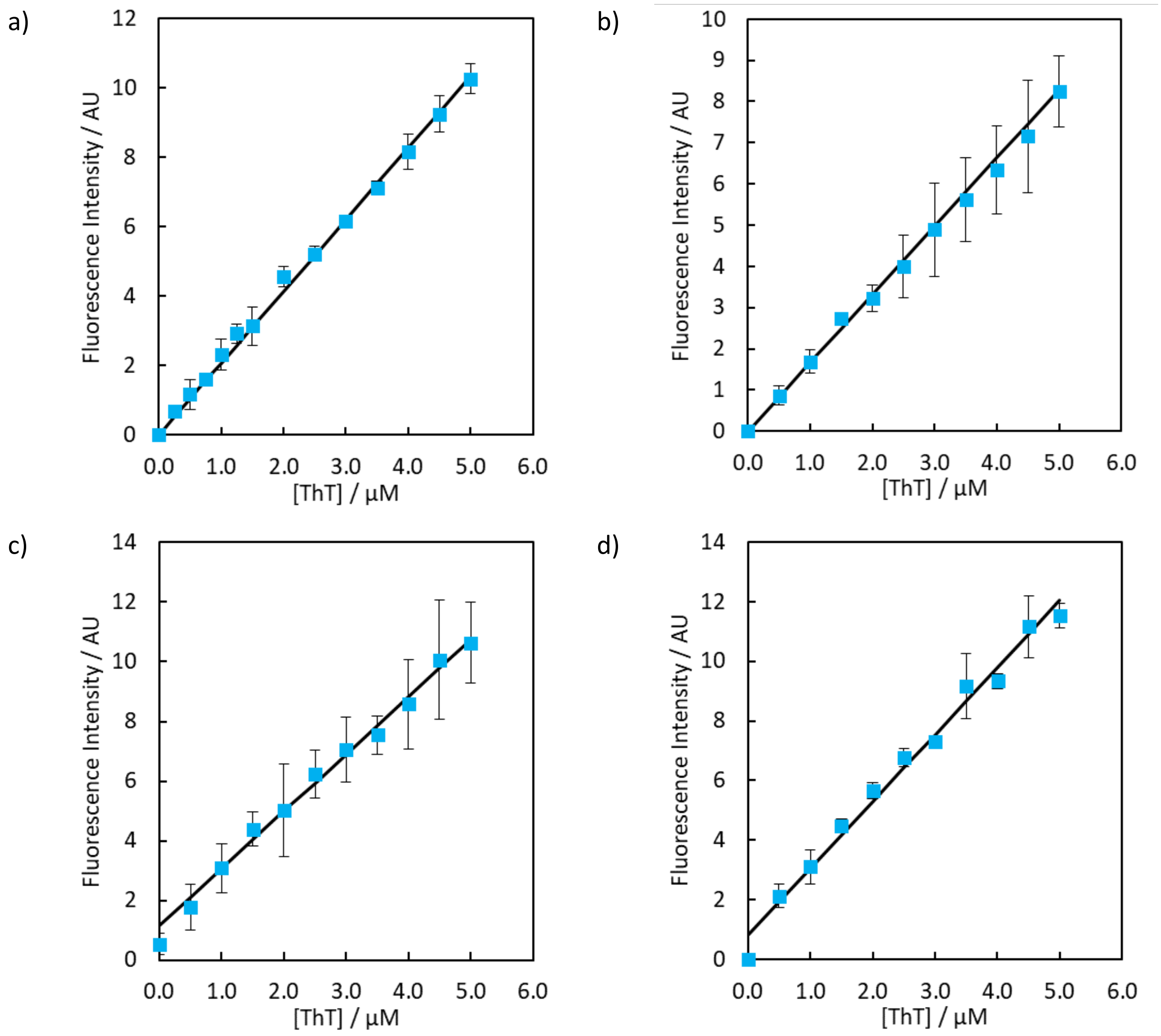

Figure S38. Dilution series of ThT into (a) 1xPBS (pH 7.4, 25 °C), showing a line of best fit with a slope of $(\mathbf{1.9} \pm \mathbf{0.2}) \times \mathbf{10^6}\ \boldsymbol{M^{-1}}$ and a y-intercept of 0; (b) ThR (2.0 µM) in 1xPBS (pH 7.4, 25 °C), showing a line of best fit with a slope of $(\mathbf{1.7} \pm \mathbf{0.2}) \times \mathbf{10^6}\ \boldsymbol{M^{-1}}$ and a y-intercept of 0; (c) OXI (2.0 µM) in 1xPBS (pH 7.4, 25 °C), showing a line of best fit with a slope of $(\mathbf{1.9} \pm \mathbf{0.4}) \times \mathbf{10^6}\ \boldsymbol{M^{-1}}$ and a y-intercept of 1.4 ± 0.6; and (d) S5H (2.0 µM) in 1xPBS (pH 7.4, 25 °C), showing a line of best fit with a slope of $(\mathbf{2.2} \pm \mathbf{0.1}) \times \mathbf{10^6}\ \boldsymbol{M^{-1}}$ and a y-intercept of 0.8 ± 0.3. Spectra were recorded using λ_{ex} = 440 nm and monitoring emission at λ_{em} = 483 nm. The experimental measurements are shown as points (error bars represent the 95% confidence interval calculated from at least three independent experiments).

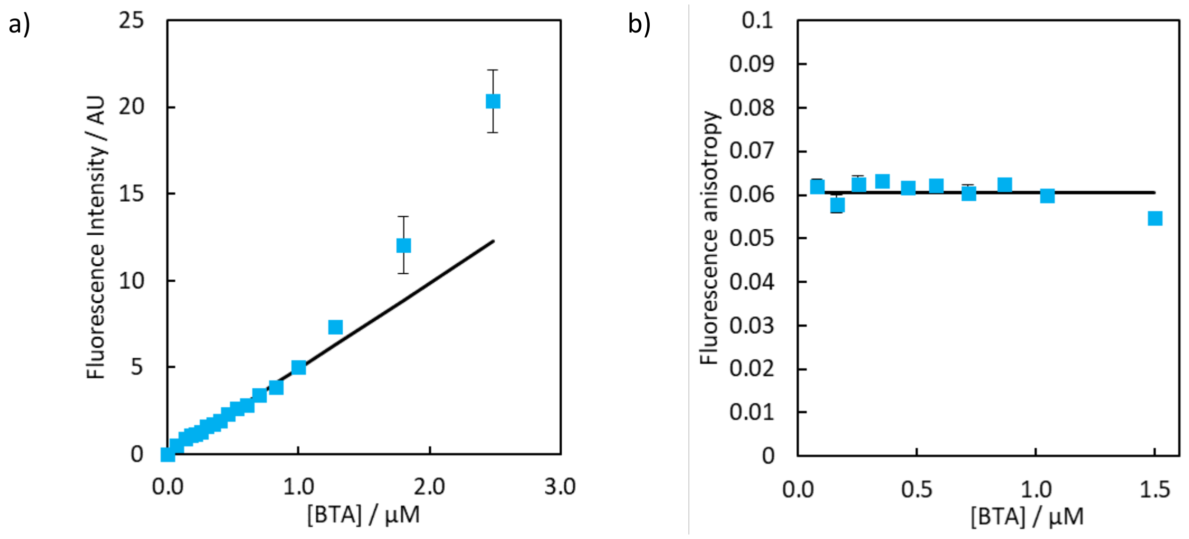

Figure S39. (a) Dilution series of BTA into 1xPBS (pH 7.4, 25 °C), showing a line of best fit with a slope of $(\mathbf{4.9} \pm \mathbf{0.2}) \times \mathbf{10^6}\ \boldsymbol{M^{-1}}$ and a y-intercept of 0. Spectra were recorded using λ_{ex} = 360 nm and monitoring emission at λ_{em} = 443 nm. (b) Fluorescence anisotropy dilution series of BTA into 1xPBS (pH 7.4, 25 °C). Fluorescence anisotropy measurements were recorded using λ_{ex} = 360 nm and monitoring emission at λ_{em} = 443 nm. The average anisotropy value of the free ligand was 0.061 ± 0.001. The experimental measurements are shown as points (error bars represent the 95% confidence interval calculated from at least three independent experiments).

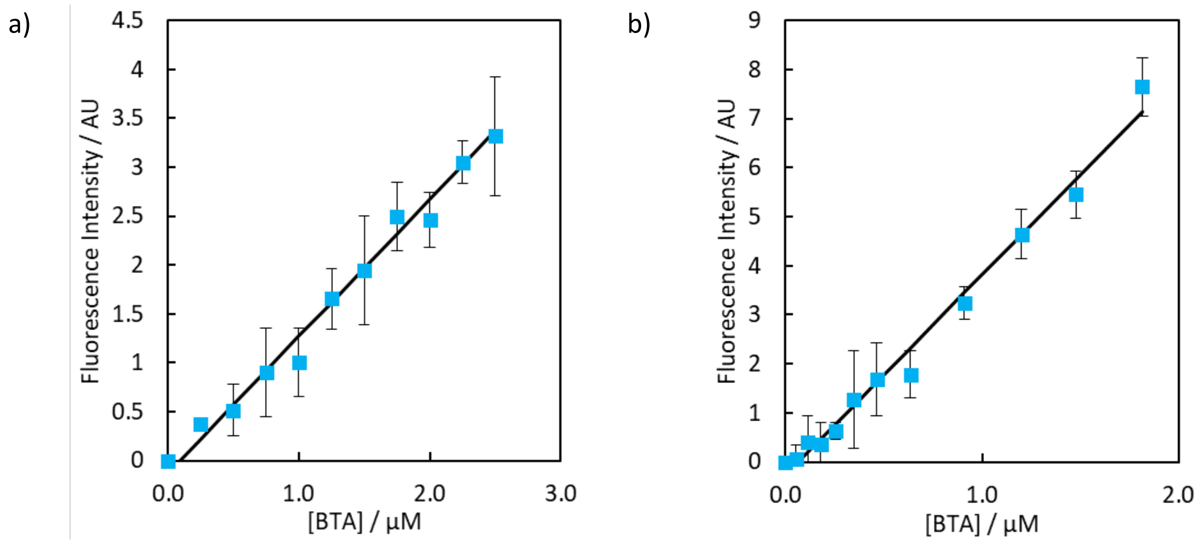

Figure S40. Dilution series of BTA into (a) ThT (1.0 µM) in 1xPBS (pH 7.4, 25 °C), showing a line of best fit with a slope of $(\mathbf{1.4} \pm \mathbf{0.1}) \times \mathbf{10^6}\ \boldsymbol{M^{-1}}$ and a y-intercept of -0.1 ± 0.2; (b) OXI (2.38 µM) and ThT (1.0 µM) in 1xPBS (pH 7.4, 25 °C), showing a line of best fit with a slope of $(\mathbf{4.1} \pm \mathbf{0.3}) \times \mathbf{10^6}\ \boldsymbol{M^{-1}}$ and a y-intercept of -0.3 ± 0.4. Spectra were recorded using λ_{ex} = 440 nm and monitoring emission at λ_{em} = 483 nm. The experimental measurements are shown as points (error bars represent the 95% confidence interval calculated from at least three independent experiments).

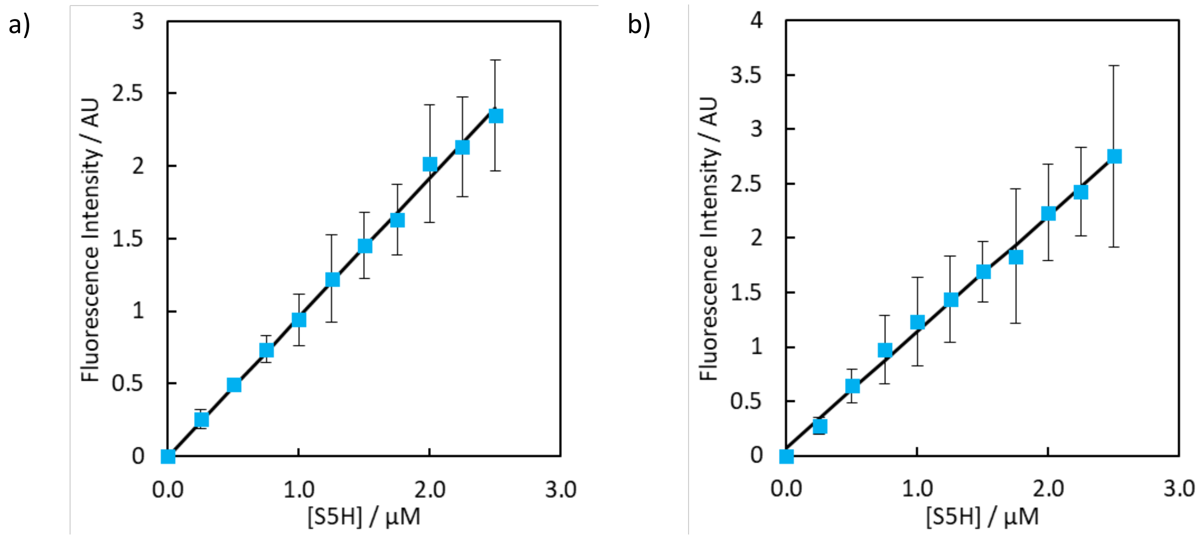

Figure S41. Dilution series of S5H into (a) 1xPBS (pH 7.4, 25 °C), showing a line of best fit with a slope of $(\mathbf{9} \pm \mathbf{2}) \times \mathbf{10^5}\ \boldsymbol{M^{-1}}$ and a y-intercept of 0; (b) ThT (1.0 µM) in 1xPBS (pH 7.4, 25 °C), showing a line of best fit with a slope of $(\mathbf{1.1} \pm \mathbf{0.3}) \times \mathbf{10^6}\ \boldsymbol{M^{-1}}$ and a y-intercept of 2.4 ± 0.3. Spectra were recorded using λ_{ex} = 440 nm and monitoring emission at λ_{em} = 483 nm. The experimental measurements are shown as points (error bars represent the 95% confidence interval calculated from at least three independent experiments).

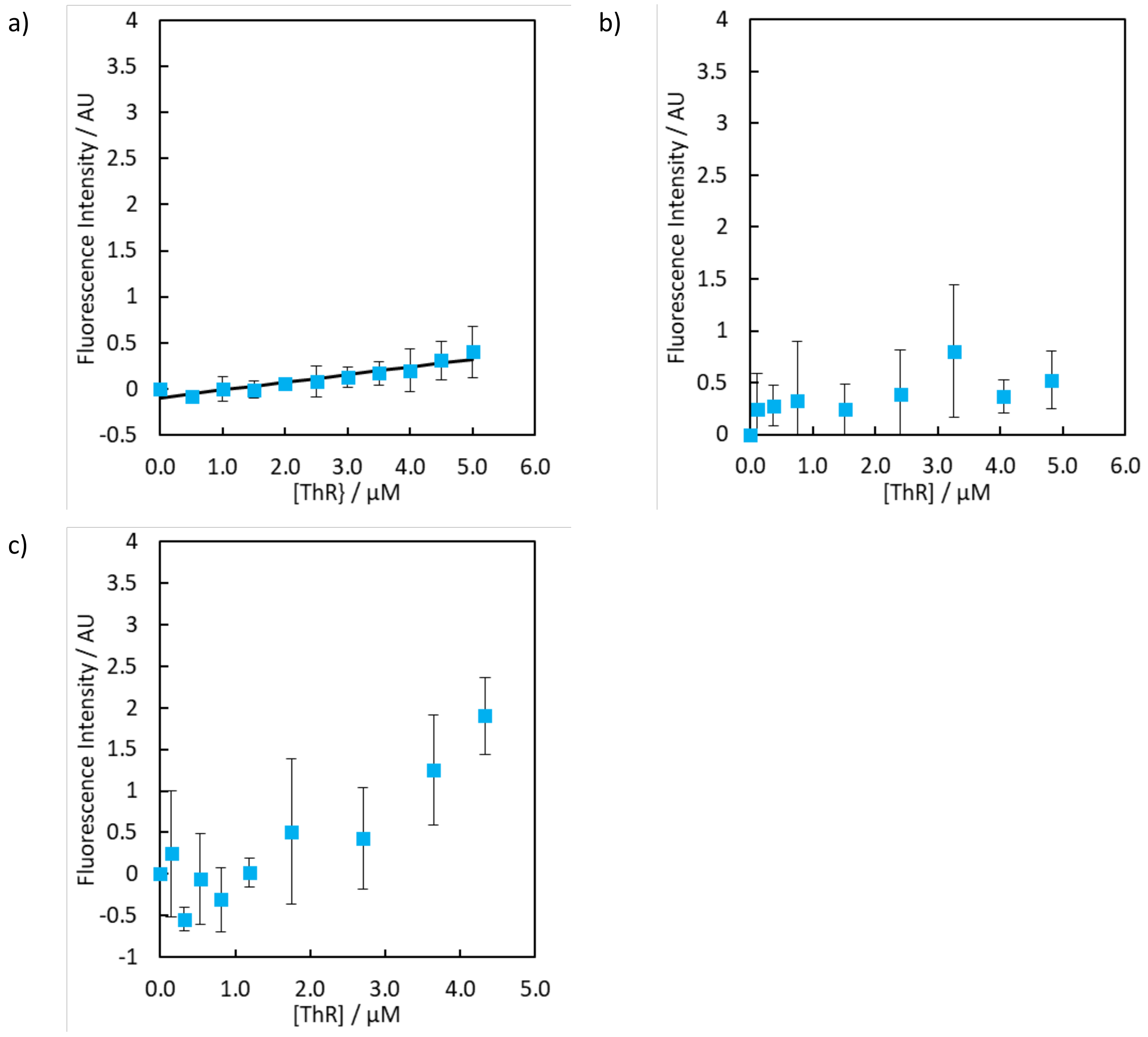

Figure S42. Dilution series of ThR into (a) ThT (1.0 µM) in 1xPBS (pH 7.4, 25 °C), showing a line of best fit with a slope of $(\mathbf{8} \pm \mathbf{4}) \times \mathbf{10^4}\ \boldsymbol{M^{-1}}$ and a y-intercept of -0.1 ± 0.1; (b) ThT (1.0 µM) and OXI (2.0 µM) in 1xPBS (pH 7.4, 25 °C); (c) ThT (1.0 µM) and S5H (2.0 µM) in 1xPBS (pH 7.4, 25 °C). Spectra were recorded using λ_{ex} = 440 nm and monitoring emission at λ_{em} = 483 nm. The experimental measurements are shown as points (error bars represent the 95% confidence interval calculated from at least three independent experiments).

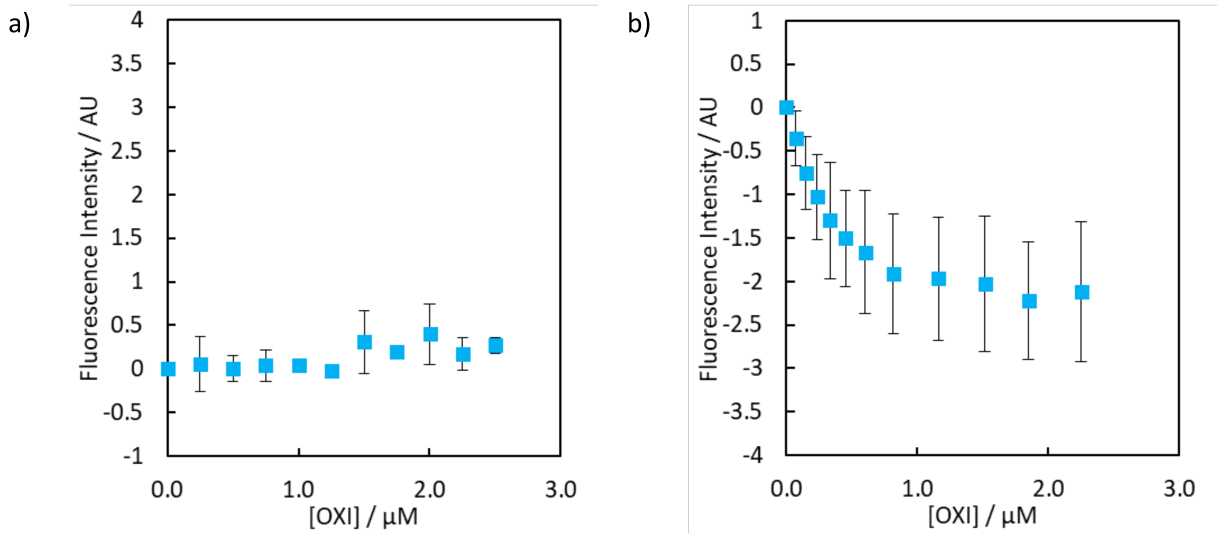

Figure S43. Dilution series of OXI into (a) ThT (1.0 µM) in 1xPBS (pH 7.4, 25 °C); (b) into ThT (1.0 µM) and S5H (2.0 µM) in 1xPBS (pH 7.4, 25 °C). Spectra were recorded using λ_{ex} = 440 nm and monitoring emission at λ_{em} = 483 nm. The experimental measurements are shown as points (error bars represent the 95% confidence interval calculated from at least three independent experiments).

General Procedures for Binding Assays

Fluorescence Titrations

Fluorescence spectra were measured using the general procedure described above for fluorescence characterisation. Stock solutions of ligand in DMSO at concentrations of 1.0 mM were prepared. Stock solutions of αSyn fibrils or Aβ(1-42) fibrils in 1xPBS (pH 7.4, 10 μM) were prepared. Titration solutions were prepared by diluting ligands (1.0 mM in DMSO) and amyloid fibrils (10 μM in 1xPBS) in 1xPBS (pH 7.4) to the desired concentration. All titrations were performed in 1xPBS (pH 7.4) at 25°C. All titrations were performed alongside a corresponding dilution series in the absence of any amyloid fibrils. Titrations were repeated with at least three independent replicates. Each replicate was performed using titration solutions freshly prepared from different stock solutions. Spectral experiments detecting **ThT** used λ_{ex} = 440 nm, and measured emissions from λ_{em}= 470 – 600 nm. Spectral experiments detecting **BTA** used λ_{ex} = 360 nm, and measured λ_{em}= 380 – 600 nm. Spectral experiments detecting **AAR** used λ_{ex} = 522 nm, and measured λ_{em}= 562 – 700 nm

Saturation Binding Assays

For saturation binding assays, solutions of ligand (10 μM) and amyloid fibril (500 nM) in 1xPBS (pH 7.4) were titrated into a solution of amyloid fibril (500 nM) in 1xPBS (pH 7.4) at 298 K.

Fluorescence Anisotropy Binding Assays

For fluorescence anisotropy assays, solutions of BTA (10 μM) and amyloid fibrils (500 nM) in 1xPBS (pH 7.4) were titrated into a solution of amyloid fibrils (500 nM) in 1xPBS (pH 7.4) at 298 K.

One-Step Competition Binding Assays

Solutions of competing ligand L1 (10-50 μM), ThT (1.0 μM), and amyloid fibrils (500 nM) were titrated into a solution of ThT (1.0 μM) and amyloid fibrils (500 nM) in 1xPBS (pH 7.4) at 298 K.

Two-Step Competition Binding Assays

A standard competition binding assay was first performed as above. Then, a solution of competing ligand L2 (10-50 μM), ligand L1 (S5H: 1.38 μM, OXI: 2.38 μM), ThT (1.0 μM), and αSyn fibrils (500 nM) were titrated into a solution of ligand L1 (S5H: 1.38 μM, OXI: 2.38 μM), ThT (1.0 μM) and αSyn fibrils (500 nM) in 1xPBS (pH 7.4) at 298 K. Fluorescence emission spectra were taken for ThT at λ_{ex} = 440 nm (λ_{em} = 470-600 nm).

Binding Assays Performed

Table S3. A list of the direct binding assays performed on a fluorimeter.

L0	*De novo* fibrils	*PMCA* fibrils
ThT	✓	✓
AAR	✓	✓
BTA	✓	✓

Table S4. A list of the competition binding assays performed on a fluorimeter.

L0	L1	*De novo* fibrils	*PMCA* fibrils
ThT	OXI	✓	✓
ThT	ThR	✓	✓
ThT	BTA	✓	✓
ThT	S5H	✓	✓
AAR	ThT	×	✓

Data Fitting

Fluorescence spectra were analysed using a custom Python script written by Daniil Soloviev.

In all cases 1:1 binding models were used. Briefly, for a ligand L and binding site S, the intensity of the fluorescence emission (I) is given by Equation 1,

$$I = \epsilon_f \Phi_f [\mathrm{L}] + \epsilon_b \Phi_b [\mathrm{L} \cdot \mathrm{S}], \qquad \text{Eq. 1}$$

where $\epsilon_f \phi_f$ and $\epsilon_b \phi_b$ are the product of the UV-vis absorption extinction coefficient and the fluorescence quantum yield for free and bound L respectively, [L] is the concentration of free L, and $[\mathrm{L} \cdot \mathrm{S}]$ is the concentration of L bound to S. The quantity $\epsilon_f \Phi_f$ was measured using dilution series in the absence of host.

Equation 2 is used to fit anisotropy data,

$$r = r_f \frac{[\mathrm{L}]}{[L_{tot}]} + r_b \frac{[\mathrm{L \cdot S}]}{[L_{tot}]}, \qquad \text{Eq. 2}$$

where r is the measured anisotropy of the system, r_f is the anisotropy of the free ligand, and r_b is the anisotropy of the bound ligand.

The concentration of L bound to S is then given by Equation 3,

$$K_d [\mathrm{L} \cdot \mathrm{S}] = [\mathrm{L}][\mathrm{S}], \qquad \text{Eq. 3}$$

where [S] is the concentration of unbound site S, and K_d is the dissociation constant of L binding to S. The total concentration of L, $[\mathrm{L_{tot}}]$, is then given by Equation 4,

$$[\mathrm{L_{tot}}] = [\mathrm{L} \cdot \mathrm{S}] + [\mathrm{L}]. \qquad \text{Eq. 4}$$

Multiple ligands and binding sites can be considered, which is required for competition binding assays. The total concentration of binding site, $[\mathrm{L_{tot}}]$, is also optimised to avoid assumptions about the stoichiometry of binding sites to protein concentration. For competition binding assays the reporting ligand (typically ThT) was assumed to bind to two binding sites with an identical dissociation constant to form complexes with an identical optical brightness. The competing ligand being titrated was assumed to target only one of these sites.

Saturation Binding Assays

Direct saturation binding assays were performed according to general methods.

ThT (L0)

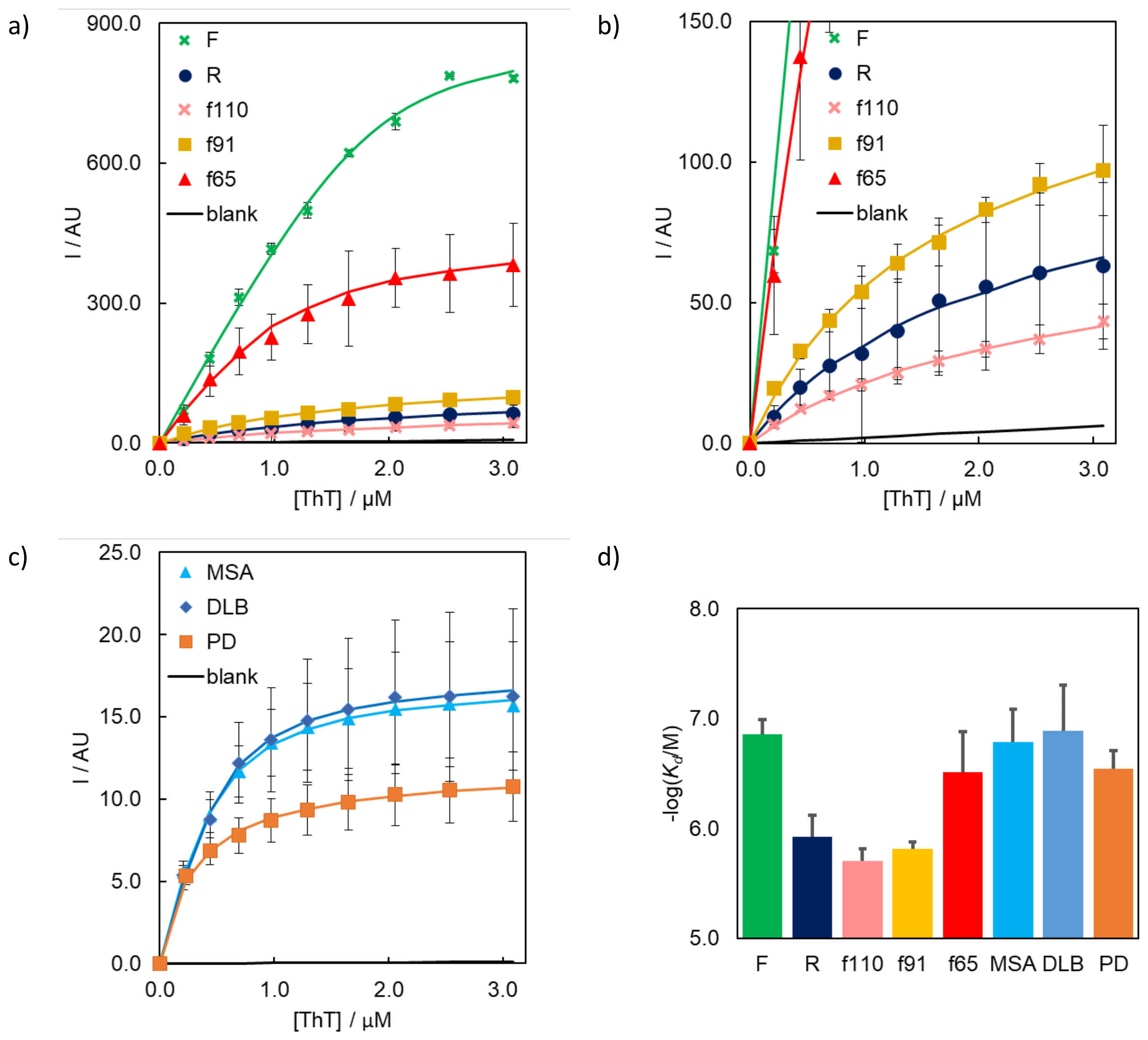

Figure S44. Data from fluorescence titrations of ThT (λ_{ex} = 440 nm, λ_{em} = 483 nm) into (a) *de novo* αSyn fibrils (500 nM), (b) an expanded view of subfigure (a), and (c) *PMCA* αSyn fibrils (500 nM), in aqueous 1xPBS (pH 7.4, 25 °C). (d) Comparison of the binding constants fitted from 1:1 binding isotherms. Datapoints are the average of at least three experimental measurements with 95% confidence intervals shown, and lines are the best fit to a 1:1 binding isotherm.

AAR (L0)

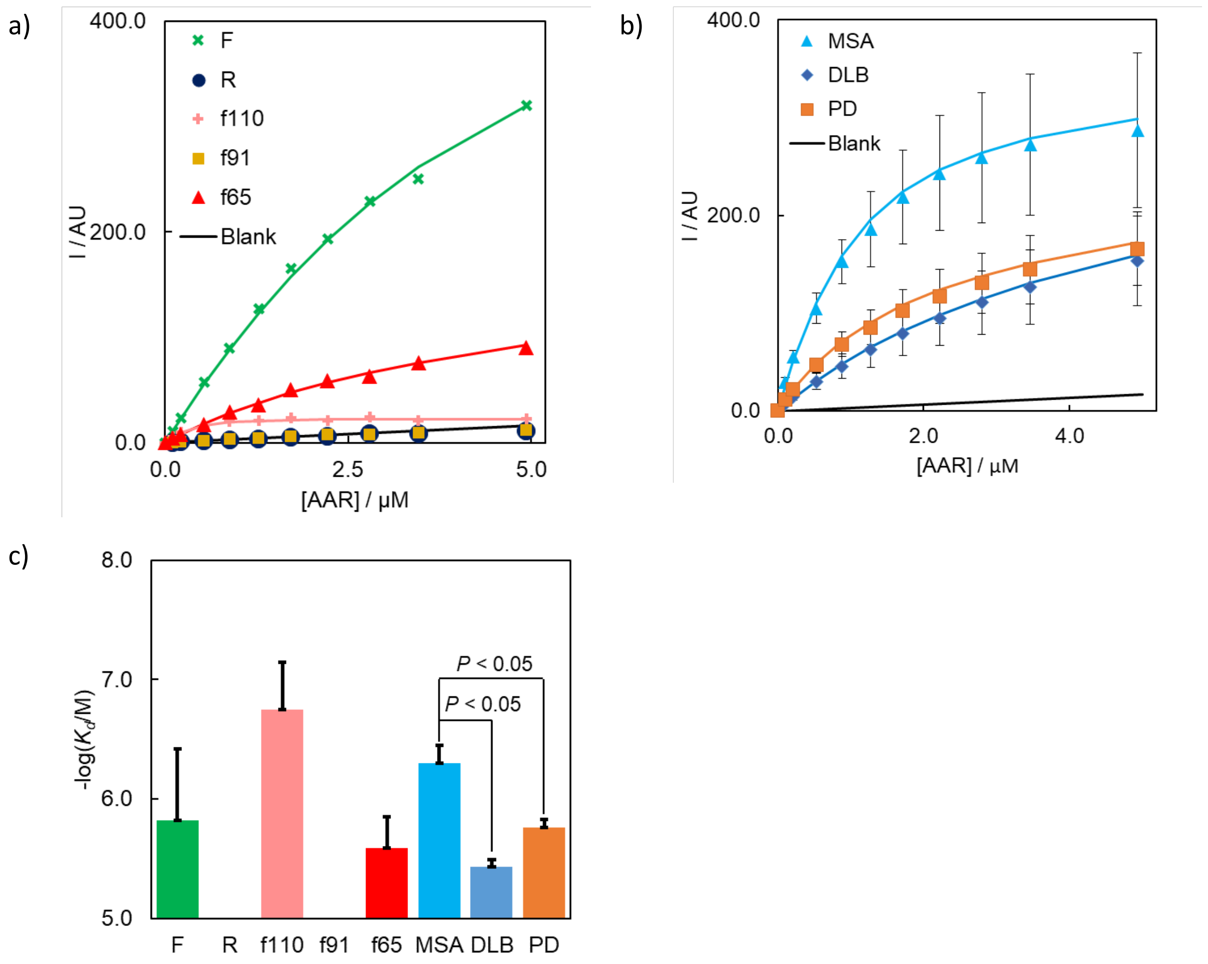

Figure S45. Data from fluorescence titrations of AAR (λ_{ex} = 522 nm, λ_{em} = 573 nm) into (a) *de novo* αSyn fibrils (500 nM), and (b) *PMCA* αSyn fibrils, in aqueous 1xPBS (pH 7.4, 25 °C). Lines are the best fit to a 1:1 binding isotherm. c) Comparison of the binding constants fitted from 1:1 binding isotherms. Datapoints are the average of at least three experimental measurements with 95% confidence intervals shown, and *P* values were calculated using a two-sided paired *t*-test.

BTA (L0)

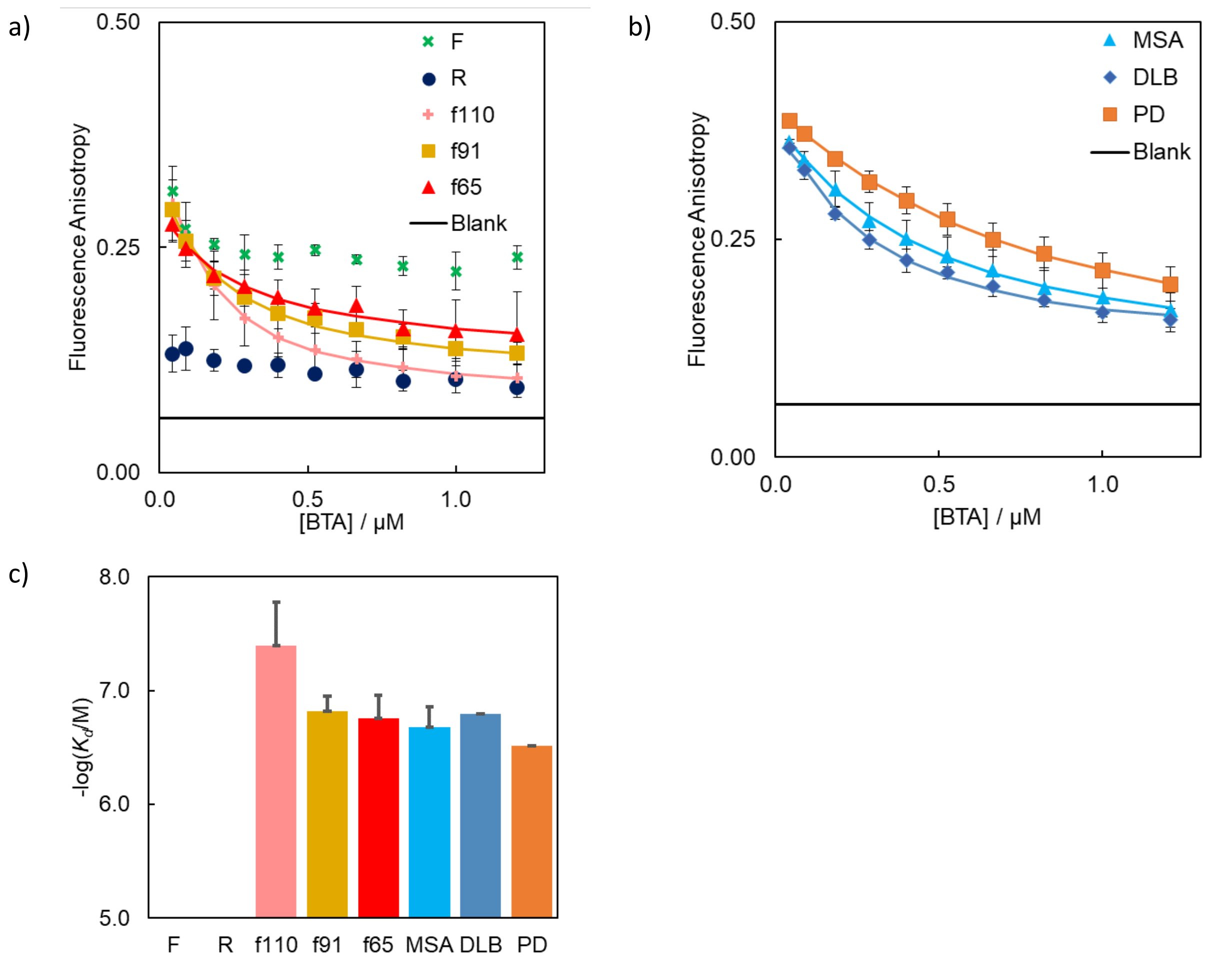

Figure S46. a) Data from fluorescence anisotropy titrations of BTA (λ_{ex} = 360 nm, λ_{em} = 443 nm) into (a) *de novo* αSyn fibrils (500 nM), and (b) PMCA αSyn fibrils, in aqueous 1xPBS (pH 7.4, 25 °C). Datapoints are the average of at least three experimental measurements with 95% confidence intervals shown, and lines are the best fit to a 1:1 binding isotherm.

Dissociation Constants

Table S5. Dissociation constants from direct binding assays.[a]

Ligand	-log(K_d/M) for target fibril							
	F	R	f110	f91	f65	MSA	DLB	PD
ThT	6.9 ± 0.1	5.7 ± 0.1	6.5 ± 0.4	5.8 ± 0.1	5.9 ± 0.2	6.5 ± 0.2	6.9 ± 0.4	6.5 ± 0.2
AAR	6.4 ± 0.6	-	n.d.	-	5.6 ± 0.3	6.3 ± 0.1	5.4 ± 0.1	5.8 ± 0.1
BTA (FA)	-	-	7.4 ± 0.3	6.8 ± 0.1	6.8 ± 0.4	6.7 ± 0.1	6.8 ± 0.2	6.5 ± 0.2

[a] Errors represent a 95% confidence interval calculated from at least three independent experiments. "-": no binding observed. "n.d.": the dissociation constant could not be determined from the collected data. "FA": fluorescence anisotropy.

One-Step Competition Binding Assays

One-step competition binding assays were performed according to general methods.

BTA (L1) into ThT (L0)

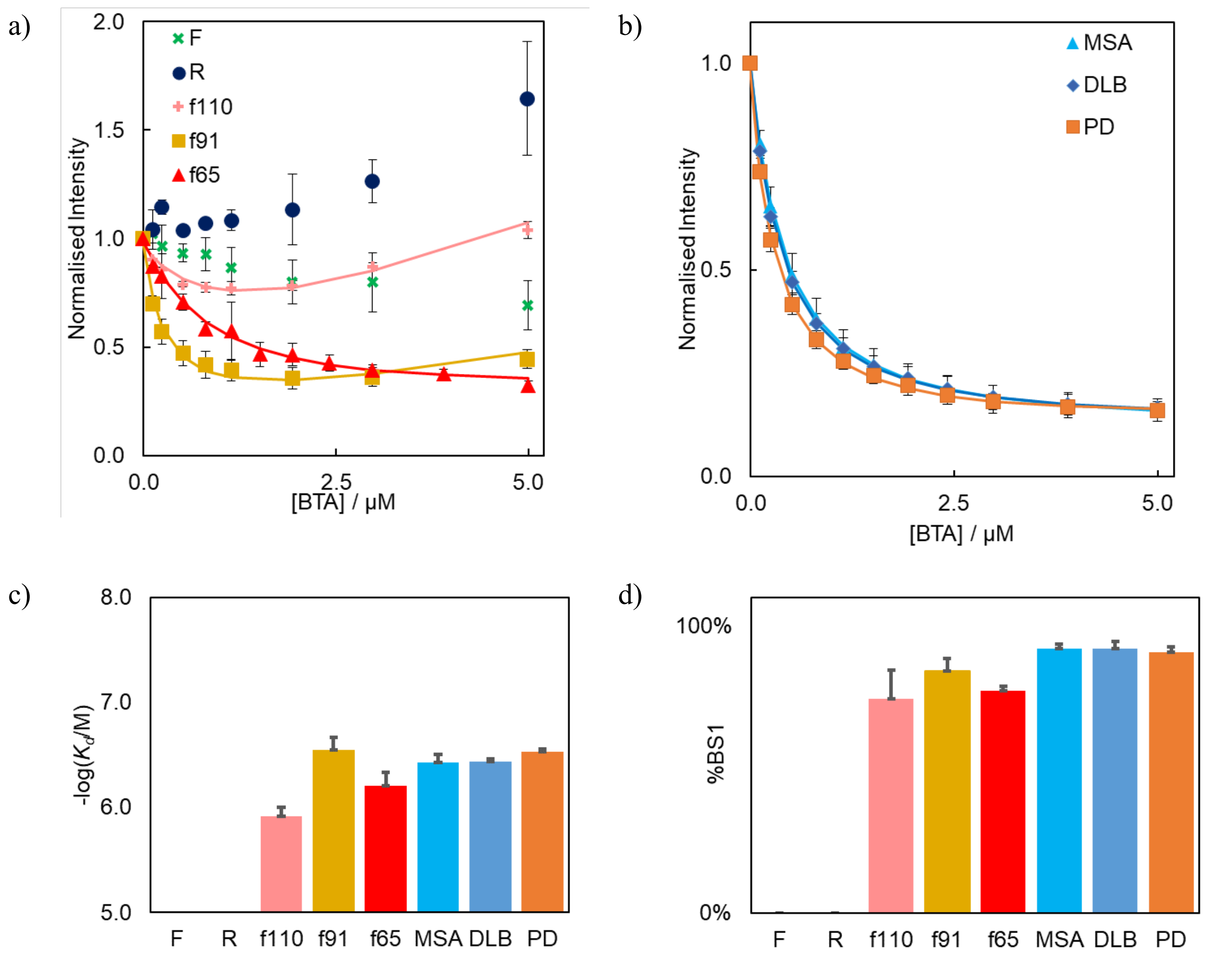

Figure S47. Data from fluorescence competition assays (λ_{ex} = 440 nm, λ_{em} = 483 nm) for titrating BTA (L1) into a mixture of (a) *de novo* αSyn fibrils (500 nM), or (b) PMCA αSyn fibrils (500 nM), and ThT (L0, 1.0 μM) in aqueous 1xPBS (pH 7.4, 25 °C). (c) Comparison of the binding constants of BTA to the studied fibrils, and (d) comparison of the percentage of ThT binding sites accessible to BTA (%BS1) on each studied fibril. Datapoints are the average of at least three experimental measurements with 95% confidence intervals shown, and lines are the best fit to a 1:1 binding isotherm.

OXI (L1) into ThT (L0)

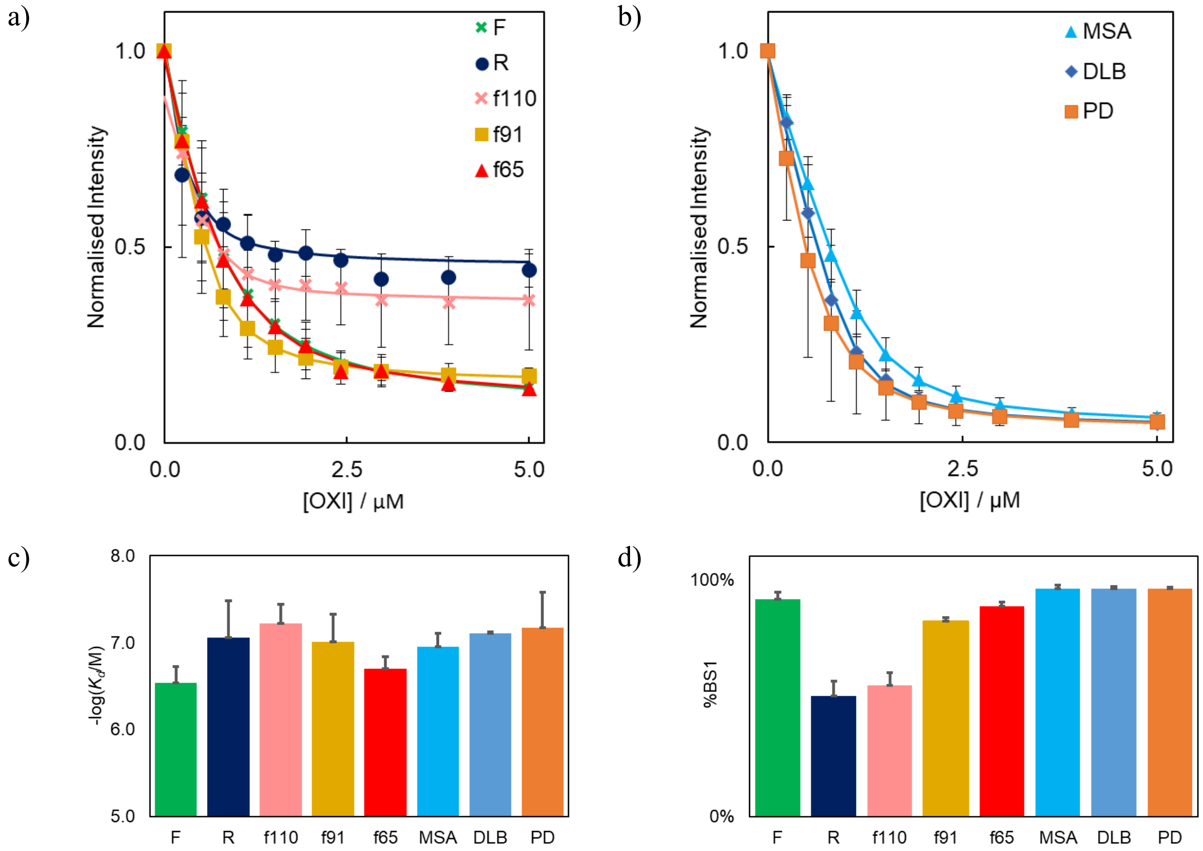

Figure S48. Data from fluorescence competition assays (λ_{ex} = 440 nm, λ_{em} = 483 nm) for titrating OXI (L1) into a mixture of (a) *de novo* αSyn fibrils (500 nM), or (b) PMCA αSyn fibrils (500 nM), and ThT (L0, 1.0 µM) in aqueous 1xPBS (pH 7.4, 25 °C). (c) Comparison of the binding constants of OXI to the studied fibrils, and (d) comparison of the percentage of ThT binding sites accessible to OXI (%BS1) on each studied fibril. Datapoints are the average of at least three experimental measurements with 95% confidence intervals shown, and lines are the best fit to a 1:1 binding isotherm.

ThR (L1) into ThT (L0)

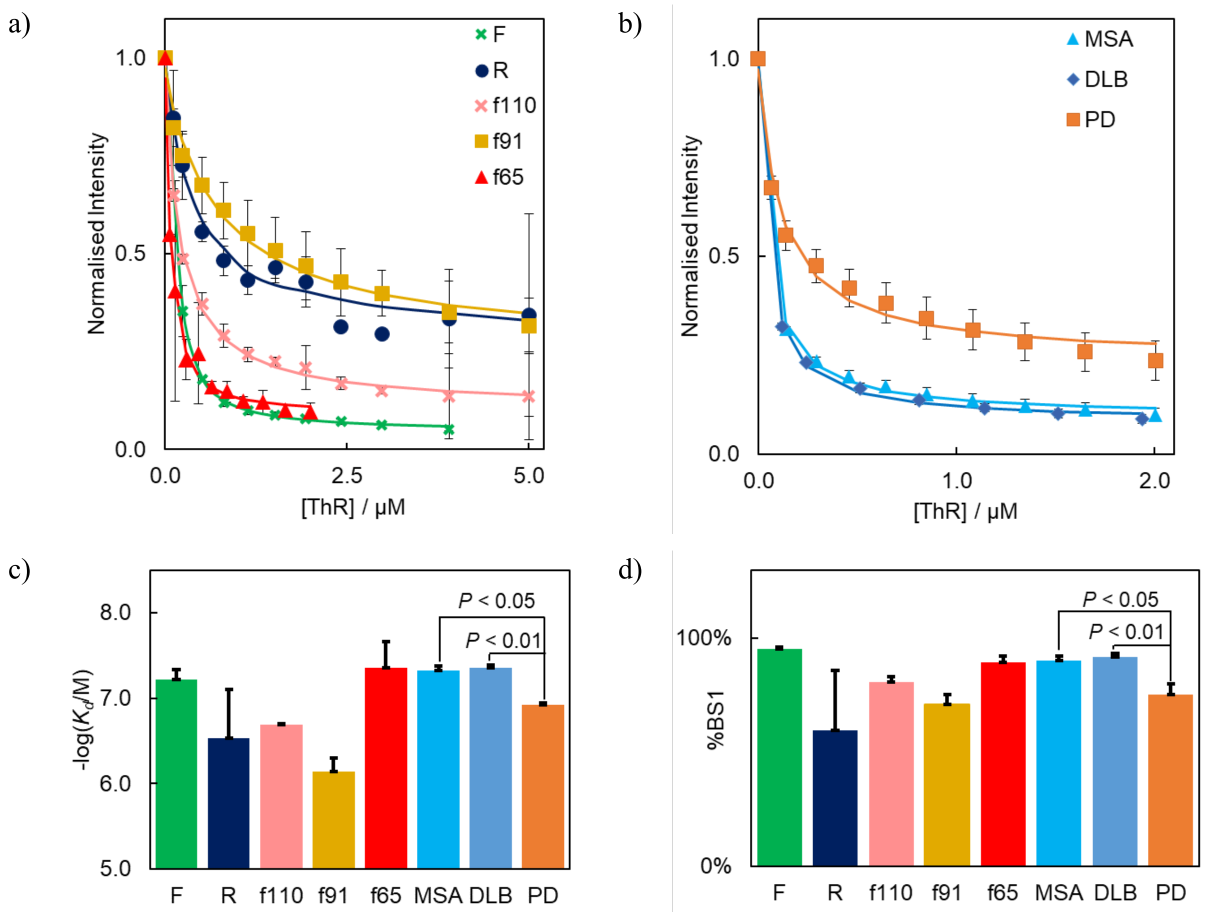

Figure S49. Data from fluorescence competition assays (λ_{ex} = 440 nm, λ_{em} = 483 nm) for titrating ThR (L1) into a mixture of (a) *de novo* αSyn fibrils (500 nM), or (b) PMCA αSyn fibrils (500 nM), and ThT (L0, 1.0 μM) in aqueous 1xPBS (pH 7.4, 25 °C). (c) Comparison of the binding constants of ThR to the studied fibrils, and (d) comparison of the percentage of ThT binding sites accessible to ThR (%BS1) on each studied fibril. Datapoints are the average of at least three experimental measurements with 95% confidence intervals shown, and lines are the best fit to a 1:1 binding isotherm. *P* values were calculated using a two-sided paired *t*-test.

S5H (L1) into ThT (L0)

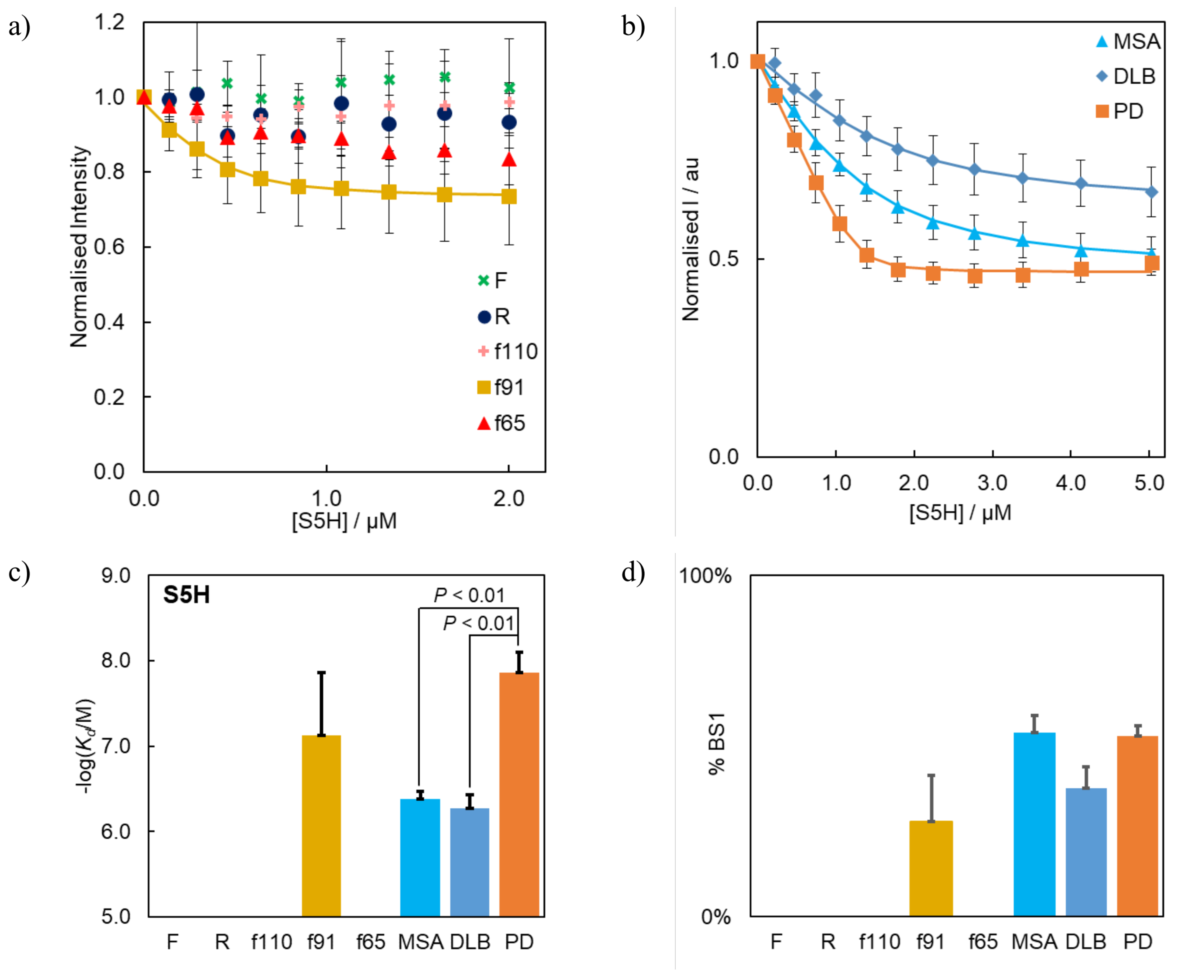

Figure S50. Data from fluorescence competition assays (λ_{ex} = 440 nm, λ_{em} = 483 nm) for titrating S5H (L1) into a mixture of (a) *de novo* αSyn fibrils (500 nM), or (b) PMCA αSyn fibrils (500 nM), and ThT (L0, 1.0 μM) in aqueous 1xPBS (pH 7.4, 25 °C). (c) Comparison of the binding constants of S5H to the studied fibrils, and (d) comparison of the percentage of ThT binding sites accessible to S5H (%BS1) on each studied fibril. Datapoints are the average of at least three experimental measurements with 95% confidence intervals shown, and lines are the best fit to a 1:1 binding isotherm. *P* values were calculated using a two-sided paired *t*-test.

S5H (L1) into ThT (L0), then ThR (L2)

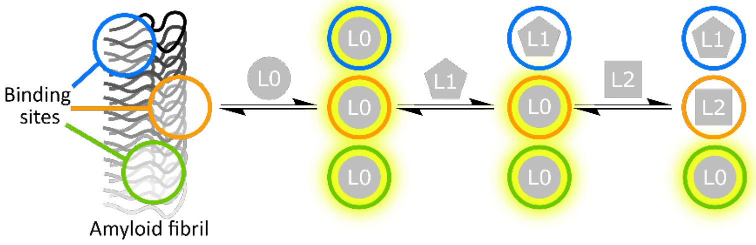

Figure S51. A two-step competition binding assay showing the binding of three ligands (L0, L1, L2) to fibril binding sites (blue, orange, and green circles). First, the solvatochromic ligand L0 is added to a sample of fibril. L0 binds to all three sites, producing an enhancement in fluorescence emission. A competition assay is then performed by titrating in the competing ligand L1 which displaces L0 from one binding site (blue), producing a change in fluorescence. A second competition binding assay is then performed using another competing ligand L2 which displaces L0 from a second site (orange).

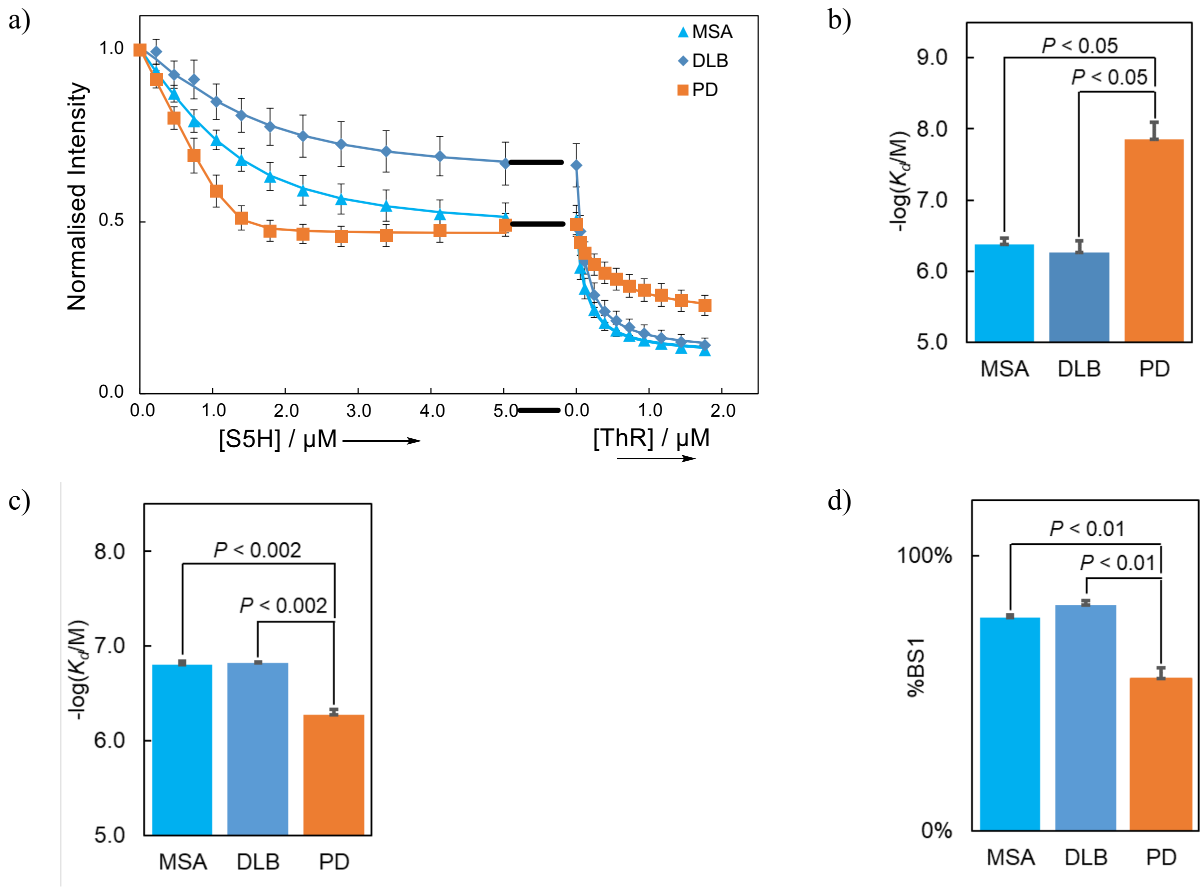

Figure S52. (a) Two-step competition assay for the sequential titration of S5H (L1) then ThR (L2) into PMCA αSyn fibrils (500 nM) and ThT (L0, 1.0 μM, λ_{ex} = 440 nm, λ_{em} = 483 nm) in aqueous 1xPBS (pH 7.4, 25 °C). The second phase of the two-step competition assay is plotted continuously after the first phase, and the horizontal dotted lines indicate a change in ligand. Lines are the best fit to 1:1 binding isotherms. (b) Comparison of the binding constants from the first S5H titration. (c) Comparison

of the binding constants from the subsequent ThR titration. (c) Comparison of the percentage of ThT binding sites accessible to ThR (%BS1) in the second phase of the assay. Data shown is the mean of three independent measurements. Error bars denote a 95% confidence interval, and *P* values were calculated using a two-sided paired *t*-test.

ThT (L1) into AAR (L0)

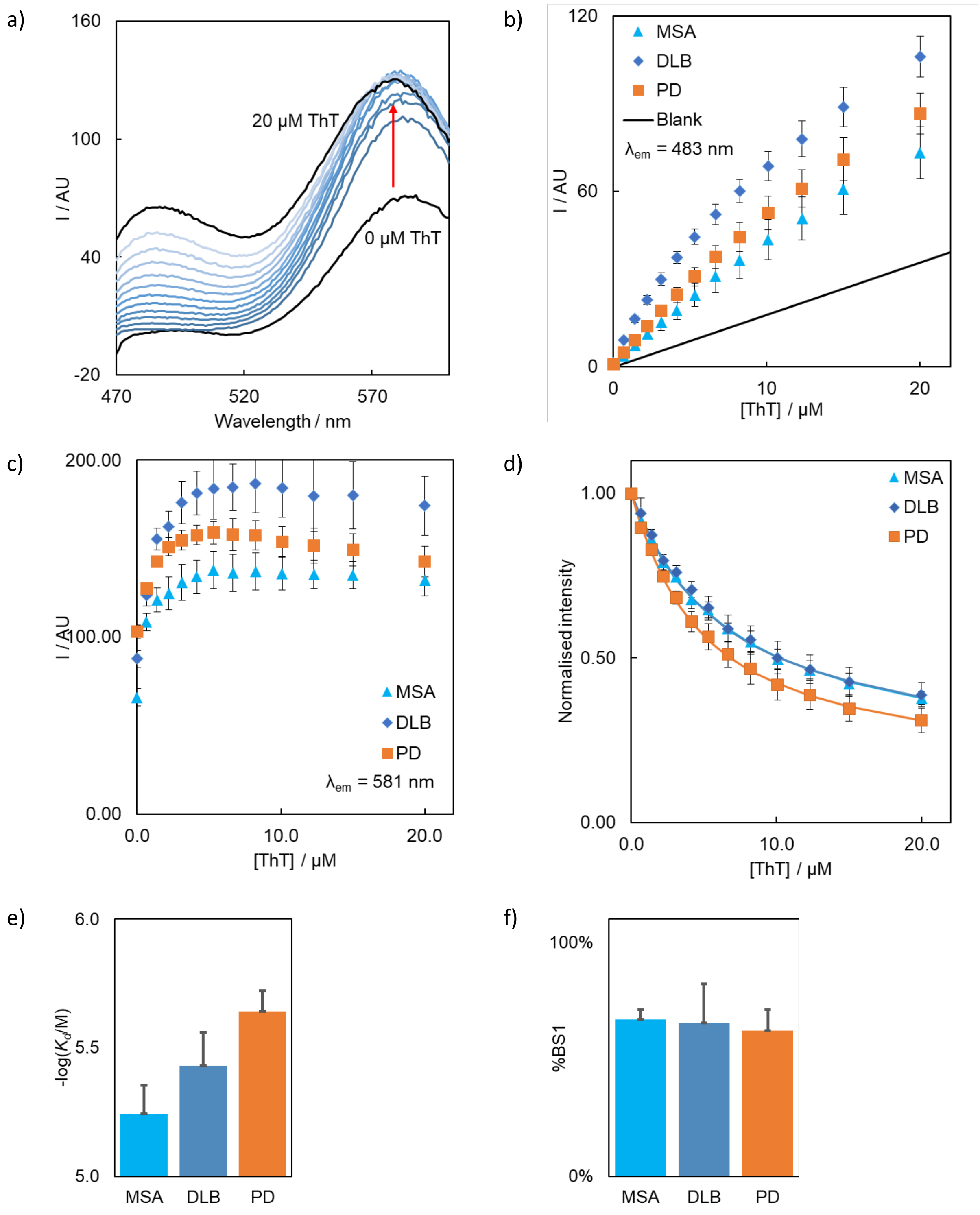

Figure S53. Fluorescence competition assays were performed by titrating ThT into a mixture of PMCA αSyn fibrils (250 nM) and AAR (5.0 µM) in aqueous 1xPBS (pH 7.4, 25 °C). (a) The spectra of an exemplar titration monitored at λ_{ex} = 440 nm, showing an enhanced emission at 581 nm. The change in fluorescence intensity of this assay was measured by exciting the mixture at λ_{ex} = 440 nm and monitoring the fluorescence emission at (b) 483 nm, and (c) 581 nm (no significant emission was measured for the blank dilution series), or (d) by exciting the mixture at λ_{ex} = 522 nm and monitoring

the fluorescence emission at 581 nm. The data from excitation at 522 nm was fitted to a 1:1 binding isotherm, allowing for (e) a comparison of the binding constants of ThT to the studied fibrils, and (f) a comparison of the percentage of AAR binding sites accessible to ThT (%BS1) on each studied fibril. Datapoints are the average of at least three experimental measurements with 95% confidence intervals shown, and lines are the best fit to a 1:1 binding isotherm.

Dissociation Constants and %BS1

Table S6. Dissociation constants from competition binding assays.[a]

Competing ligand	-log(K_d/M) for target fibril							
	F	R	f110	f91	f65	MSA	DLB	PD
OXI	6.5 ± 0.2	7.1 ± 0.4	7.2 ± 0.2	7.0 ± 0.3	6.7 ± 0.1	7.0 ± 0.2	7.1 ± 0.1	7.2 ± 0.4
BTA	-	-	5.9 ± 0.1	6.5 ± 0.1	6.2 ± 0.1	6.4 ± 0.1	6.4 ± 0.1	6.5 ± 0.1
S5H	-	-	-	7.1 ± 0.7	-	6.4 ± 0.1	6.3 ± 0.2	7.9 ± 0.2
ThR	7.2 ± 0.1	6.5 ± 0.6	6.7 ± 0.1	6.1 ± 0.2	7.4 ± 0.3	7.3 ± 0.1	7.4 ± 0.1	6.9 ± 0.1
ThR, after S5H	n.p.	n.p.	n.p.	n.p.	n.p.	6.8 ± 0.1	6.8 ± 0.1	6.3 ± 0.1

[a] Errors represent a 95% confidence interval calculated from at least three independent experiments. "-": no binding observed. "n.p.": the binding assay was not performed.

Table S7. The percentage of total ThT binding sites accessible to the competing ligand (%BS1) calculated from competition binding assays.[a]

Competing ligand	%BS1							
	F	R	f110	f91	f65	MSA	DLB	PD
OXI	92 ± 3	51 ± 6	56 ± 5	83 ± 1	89 ± 2	96 ± 2	96 ± 1	96 ± 1
BTA	-	-	73 ± 6	84 ± 4	77 ± 2	92 ± 2	92 ± 3	91 ± 2
S5H	-	-	-	30 ± 10	-	54 ± 5	38 ± 6	53 ± 3
ThR	85 ± 1	60 ± 30	81 ± 2	71 ± 4	89 ± 3	90 ± 2	92 ± 2	75 ± 5
ThR, after S5H	n.p.	n.p.	n.p.	n.p.	n.p.	77 ± 1	82 ± 2	55 ± 4

[a] Errors represent a 95% confidence interval calculated from at least three independent experiments. "-": no binding observed. "n.p.": the binding assay was not performed.

Table S8. Fitted dissociation constants from competition binding assays involving two fluorescence ligands. These dissociation constants are all from monitoring the emission of AAR (λ_{ex} = 522 nm) and fitting the data to a 1:1 binding isotherm.[a]

Titration	-log(K_d/M) for target fibril		
	MSA	DLB	PD
ThT into AAR	5.2 ± 0.1	5.4 ± 0.1	5.6 ± 0.1

[a] Errors represent a 95% confidence interval calculated from at least three independent experiments.

Table S9. The proportion of AAR binding sites occupied by a competing ligand (%BS1) calculated during the fitting procedure. These values are derived from monitoring the emission of AAR (λ_{ex} = 522 nm) and fitting the data to a 1:1 binding isotherm.

Titration	%BS1		
	MSA	DLB	PD
ThT into AAR	67 ± 4	70 ± 20	62 ± 9

[a] Errors represent a 95% confidence interval calculated from at least three independent experiments.

One-Step Competition Binding Assays with Two Fluorescent Ligands

ThT (L1) into AAR (L0)

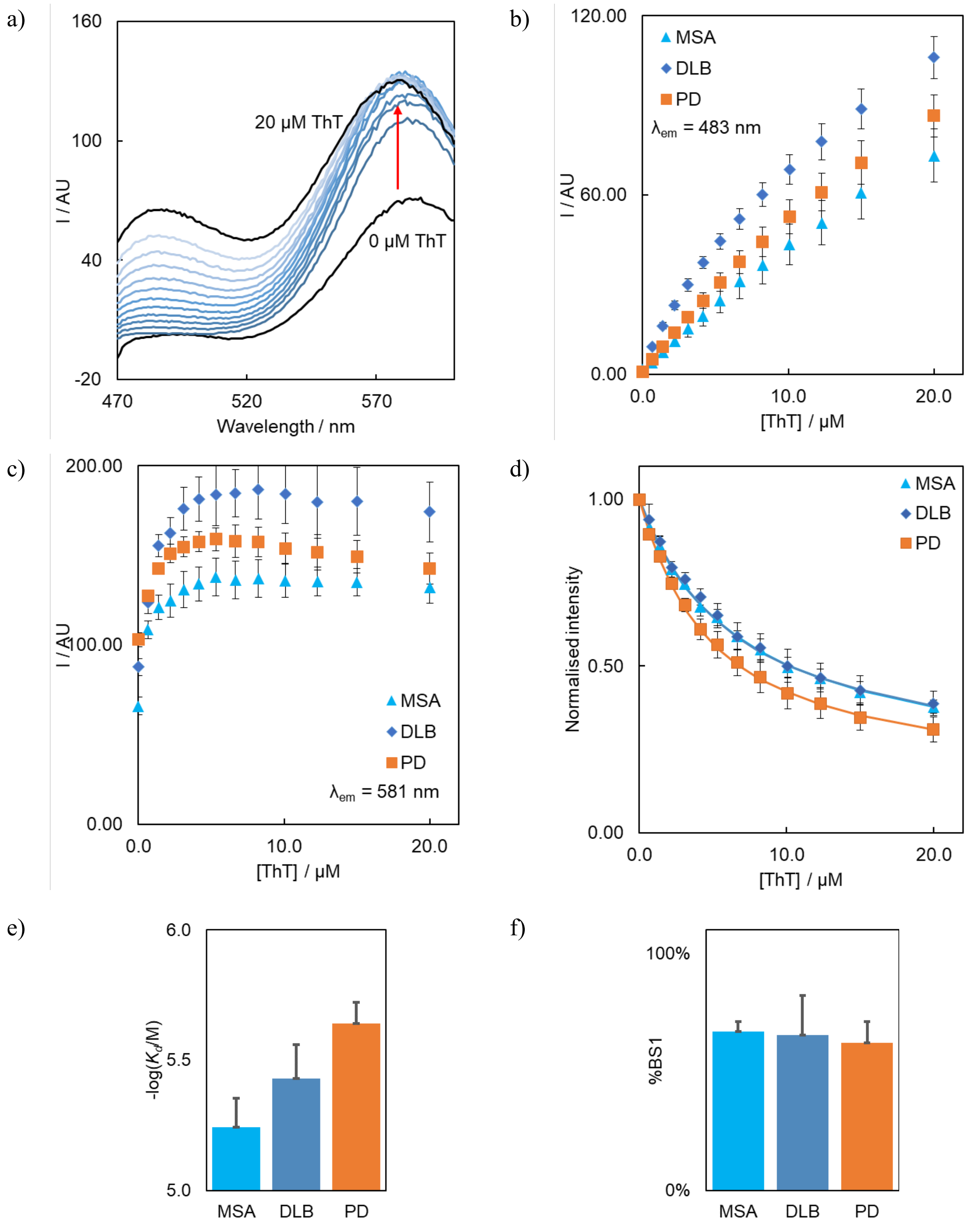

Figure S54. Fluorescence competition assays were performed by titrating ThT into a mixture of PMCA αSyn fibrils and AAR (5.0 μM) in aqueous 1xPBS (pH 7.4, 25 °C). (a) The spectra of an exemplar titration monitored at λ_{ex} = 440 nm, showing an enhanced emission at 581 nm. The change in fluorescence intensity of this assay was measured by exciting the mixture at λ_{ex} = 440 nm and

monitoring the fluorescence emission at (b) 483 nm, and (c) 581 nm, or (d) by exciting the mixture at λ_{ex} = 522 nm and monitoring the fluorescence emission at 581 nm. The data from excitation at 522 nm was fitted to a 1:1 binding isotherm, allowing for (a) a comparison of the binding constants of ThT to the studied fibrils, and (b) a comparison of the percentage of AAR binding sites accessible to ThT (%BS1) on each studied fibril. Datapoints are the average of at least three experimental measurements with 95% confidence intervals shown, and lines are the best fit to a 1:1 binding isotherm.

Dissociation Constants and %BS1

Table S10. Fitted dissociation constants from competition binding assays involving two fluorescence ligands. These dissociation constants are all from monitoring the emission of AAR (λ_{ex} = 522 nm) and fitting the data to a 1:1 binding isotherm.[a]

Titration	-log(K_d/M) for target fibril		
	MSA	DLB	PD
ThT into AAR	5.2 ± 0.1	5.4 ± 0.1	5.6 ± 0.1

[a] Errors represent a 95% confidence interval calculated from at least three independent experiments.

Table S11. The proportion of AAR binding sites occupied by a competing ligand (%BS1) calculated during the fitting procedure. These values are derived from monitoring the emission of AAR (λ_{ex} = 522 nm) and fitting the data to a 1:1 binding isotherm.[a]

Titration	%BS1		
	MSA	DLB	PD
ThT into AAR	67 ± 4	70 ± 20	62 ± 9

[a] Errors represent a 95% confidence interval calculated from at least three independent experiments.

References

(1) Chisholm, T. S.; Hunter, C. A. Ligand Profiling to Characterise Different Polymorphic Forms of α-Synuclein Aggregates. *J. Am. Chem. Soc.* **2023**, *145* (49), 27030–27037.

(2) Chu, W.; Zhou, D.; Gaba, V.; Liu, J.; Li, S.; Peng, X.; Xu, J.; Dhavale, D.; Bagchi, D. P.; D'Avignon, A.; Shakerdge, N. B.; Bacskai, B. J.; Tu, Z.; Kotzbauer, P. T.; Mach, R. H. Design, Synthesis, and Characterization of 3-(Benzylidene)Indolin-2-One Derivatives as Ligands for α-Synuclein Fibrils. *J. Med. Chem.* **2015**, *58* (15), 6002–6017.

(3) Lee, Y. H.; Denton, E. H.; Morandi, B. Modular Cyclopentenone Synthesis through the Catalytic Molecular Shuffling of Unsaturated Acid Chlorides and Alkynes. *J. Am. Chem. Soc.* **2020**, *142* (50), 20948–20955.

(4) Hsieh, C. J.; Xu, K.; Lee, I.; Graham, T. J. A.; Tu, Z.; Dhavale, D.; Kotzbauer, P.; Mach, R. H. Chalcones and Five-Membered Heterocyclic Isosteres Bind to Alpha Synuclein Fibrils in Vitro. *ACS Omega* **2018**, *3* (4), 4486–4493.

(5) Qin, L.; Vastl, J.; Gao, J. Highly Sensitive Amyloid Detection Enabled by Thioflavin T Dimers. *Mol. Biosyst.* **2010**, *6* (10), 1791–1795.

(6) Ferrie, J. J.; Lengyel-Zhand, Z.; Janssen, B.; Lougee, M. G.; Giannakoulias, S.; Hsieh, C.-J.; Pagar, V. V.; Weng, C.-C.; Xu, H.; Graham, T. J. A.; Lee, V. M.-Y.; Mach, R. H.; Petersson, E. J. Identification of a Nanomolar Affinity α-Synuclein Fibril Imaging Probe by Ultra-High Throughput in Silico Screening. *Chem. Sci.* **2020**, *11* (7), 12746–12754.

(7) Cui, M.; Ono, M.; Watanabe, H.; Kimura, H.; Liu, B.; Saji, H. Smart Near-Infrared Fluorescence Probes with Donor-Acceptor Structure for in Vivo Detection of β-Amyloid Deposits. *J. Am. Chem. Soc.* **2014**, *136* (9), 3388–3394.

(8) Ghee, M.; Melki, R.; Michot, N.; Mallet, J. PA700, the Regulatory Complex of the 26S Proteasome, Interferes with Alpha-Synuclein Assembly. *FEBS J.* **2005**, *272* (16), 4023–4033.

(9) Bousset, L.; Pieri, L.; Ruiz-Arlandis, G.; Gath, J.; Jensen, P. H.; Habenstein, B.; Madiona, K.; Olieric, V.; Böckmann, A.; Meier, B. H.; Melki, R. Structural and Functional Characterization of Two Alpha-Synuclein Strains. *Nat. Commun.* **2013**, *4* (1), 1–13.

(10) Makky, A.; Bousset, L.; Polesel-Maris, J.; Melki, R. Nanomechanical Properties of Distinct Fibrillar Polymorphs of the Protein α-Synuclein. *Sci. Rep.* **2016**, *6* (1), 37970.

(11) Shrivastava, A. N.; Bousset, L.; Renner, M.; Redeker, V.; Savistchenko, J.; Triller, A.; Melki, R. Differential Membrane Binding and Seeding of Distinct α-Synuclein Fibrillar Polymorphs. *Biophys. J.* **2020**, *118* (6), 1301–1320.

(12) Van der Perren, A.; Gelders, G.; Fenyi, A.; Bousset, L.; Brito, F.; Peelaerts, W.; Van den Haute, C.; Gentleman, S.; Melki, R.; Baekelandt, V. The Structural Differences between Patient-Derived α-Synuclein Strains Dictate Characteristics of Parkinson's Disease, Multiple

System Atrophy and Dementia with Lewy Bodies. *Acta Neuropathol. (Berl.)* **2020**, *139* (6), 977–1000.
